# A systems approach to target discovery identifies the role of lncRNA-*SPANXA2-OT1* in macrophage chemotaxis

**DOI:** 10.1172/jci.insight.191274

**Published:** 2025-10-09

**Authors:** Prabhash K. Jha, Sarvesh Chelvanambi, Yuto Nakamura, Lucas Y.U. Itto, Aatira Vijay, Adrien Lupieri, Miguel C. Barbeiro, Thanh-Dat Le, Caio B. Nascimento, Taku Kasai, Mary Whelan, Daiki Hosokawa, Dakota Becker-Greene, Sasha A. Singh, Elena Aikawa, Shizuka Uchida, Masanori Aikawa

**Affiliations:** 1 Center for Excellence in Vascular Biology and; 2 Center for Interdisciplinary Cardiovascular Sciences, Brigham and Women’s Hospital, Harvard Medical School, Boston, Massachusetts, USA.; 3 Cardiovascular Research Center, Massachusetts General Hospital, Harvard Medical School, Boston, Massachusetts, USA.; 4 Center for RNA Medicine, Department of Clinical Medicine, Aalborg University, Copenhagen, Denmark.; 5 Channing Division of Network Medicine, Brigham and Women’s Hospital, Harvard Medical School, Boston, Massachusetts, USA.

**Keywords:** Cell biology, Inflammation, Vascular biology, Macrophages, Noncoding RNAs

## Abstract

Coronary artery disease (CAD) is the leading cause of mortality worldwide, with macrophages playing a central role in shaping the inflammatory environment through cytokines, chemokines, and other mediators. Long noncoding RNAs (lncRNAs) are emerging as key regulators of cellular processes owing to their interactions with DNA, RNA, microRNAs, and proteins, which positions them to be promising therapeutic targets. Through integrative transcriptomic analysis, we identified *SPANXA2-OT1* as a primate-specific lncRNA with a potential role in macrophage-mediated inflammation in CAD. Functional studies in primary human macrophages demonstrated that *SPANXA2-OT1* was induced by inflammatory stimulation, localized to the cytoplasm, and exerted regulatory effects on chemokine expression and macrophage chemotaxis. Mechanistically, *SPANXA2-OT1* acted as a molecular sponge for microRNA-338, thereby influencing the expression of *IL-8*, a critical mediator of monocyte recruitment and inflammatory signaling. Collectively, these findings establish *SPANXA2-OT1* as a human-specific regulator of inflammatory pathways in CAD and highlight its translational potential as both a biomarker and therapeutic target.

## Abstract 2

Human-specific RNA SPANXA2-OT1 regulates macrophage-driven inflammation in coronary artery disease, offering promise as a biomarker and therapeutic target to reduce cardiovascular risk.

## Graphical abstract

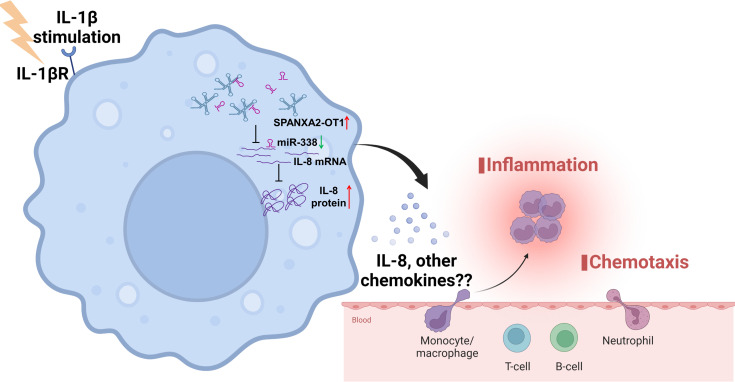

## Introduction

Noncoding RNAs, a class of functional RNAs, regulate gene expression at multiple levels. Although lncRNAs are traditionally defined as non-protein-coding, recent studies have shown that a subset can associate with ribosomes and, in some cases, produce functional micropeptides (1, 2). Long noncoding RNAs (lncRNAs) are transcribed RNA molecules longer than 200 nucleotides. These lncRNAs are frequently transcribed and expressed at various pathological events, serving as biomarkers for many diseases (3, 4), including coronary artery disease (CAD) (5, 6). Despite the availability of potent drugs that modulate traditional risk factors, CAD remains the leading cause of mortality worldwide. Evidence suggests that inflammation has a role in the development and clinical complications of atherosclerosis (7–9). LncRNAs exert a variety of regulatory functions by binding to DNA, coding or noncoding RNAs, and proteins. Similarly, microRNAs (miRNAs) regulate gene expression via post-transcriptional inhibition of target mRNAs. Multiple lines of evidence associated altered expression of lncRNAs and miRNAs with diseases. miRNAs in particular has been studied for its role in macrophage and atherosclerosis (10, 11). Previous studies characterized the roles of miRNAs in cardiovascular diseases, e.g., *miRNA-145* in venous thrombosis (12); *miRNA-33*, *miR-133b*, and *miR-21* as biomarkers for early prediction of CAD (13, 14); and circulating lncRNAs *IFNG-AS1* and *CoroMarker* as biomarkers for CAD (6, 15). Accumulating evidence established the potential role of lncRNAs acting as miRNA sponges by sequestering miRNA molecules, which in turn suppress miRNA activity, resulting in consequent increases in the levels of miRNA targets (16–18). This classical phenomenon introduces a dimension for therapeutic manipulation of miRNAs and noncoding RNAs.

Macrophage chemotaxis plays a crucial role in the pathophysiology of CAD. Circulating monocytes are recruited to sites of vascular injury or atherosclerotic plaques through chemotactic signals, primarily mediated by chemokines such as *CCL2* and its receptor *CCR2*, where they differentiate into macrophages and contribute to local inflammation and plaque progression (19). These macrophages contribute to the inflammatory response by engulfing lipids and releasing proinflammatory cytokines, which exacerbate plaque formation and instability (20). This process underscores the importance of targeting macrophage chemotaxis in therapeutic strategies for CAD.

Weighted gene coexpression network analysis (WGCNA) is a pairwise correlation analysis method that identifies key players within gene modules. WGCNA is widely used in systems biology and describes the correlation patterns among genes across microarray samples. This algorithm is used for unsupervised hierarchical clustering to understand functions of lncRNAs based on the coexpression network comprising coding and noncoding RNAs. In the present study, we performed a meta-analysis of the gene expression profiles from public databases to identify differentially expressed lncRNA (DEL), miRNA, and mRNA, independently, in CAD, followed by pathway enrichment analysis. WGCNA provided key genes followed by coregulatory network construction of lncRNA-miRNA-mRNA triads. The present study generated a comprehensive regulatory interaction network of noncoding RNAs and their targets and further showed a multilevel regulation via lncRNAs in CAD. The results provide regulatory coding-noncoding RNA triads. Our study associated the increased levels of the lncRNA, *SPANXA2-OT1*, with the sponging of *miR-338* in human primary macrophages. Furthermore, we provided the mechanistic evidence that *IL-8* and *SPANXA2-OT1* are direct targets for *miR-338* via binding at the 3′-UTR of *IL-8*. The sponging of *miR-338* by *SPANXA2-OT1* resulted in the increased expression of *IL-8*, which may favor proinflammatory mechanisms for CAD. Global transcriptomics and proteomics on *SPANXA2-OT1–*silenced human primary macrophages demonstrated altered chemokine/chemotaxis signatures. CRISPR/Cas9-mediated deletion of the *SPANXA2-OT1* functional domain resulted in decreased macrophage chemotaxis.

## Results

### Eligible microarray datasets for regulatory triad analysis in CAD.

**Figure 1:**
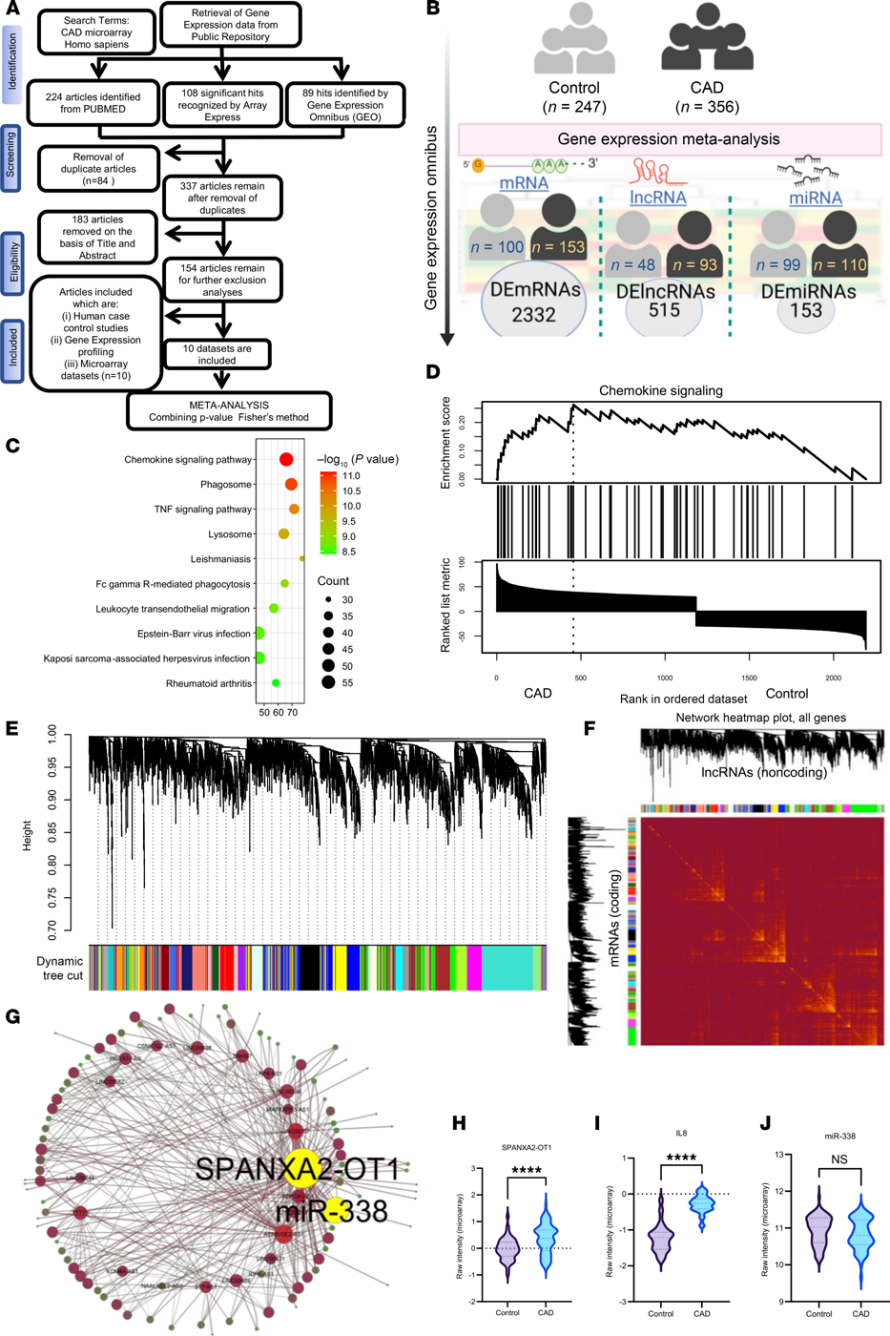
Gene expression meta-analysis and WGCNA for target prediction in CAD. (**A**) Selection process for eligible mRNA microarray datasets for meta-analysis of the gene signatures in CAD, according to PRISMA flow diagram. (**B**) Schematic of gene expression meta-analysis representing the number of human samples included and differentially expressed genes (DEGs). We integrated 10 datasets and a total of *n* = 247 samples from individuals acting as healthy controls and *n* = 356 samples from patients with CAD for the meta-analysis. (**C**) Bubble plot of KEGG pathway enrichment analysis of DEGs; *x* axis represents enrichment score. Count indicates the number of DEGs enriched in pathway; *P* values were corrected by Benjamini-Hochberg method. (**D**) Gene set enrichment analysis representation of chemokine signaling between CAD and control groups. (**E**) WGCNA-based clustering dendrogram of genes, with dissimilarity based on topological overlap, together with assigned module colors. (**F**) Visualizing the gene network using a heatmap plot, which depicts the topological overlap matrix (TOM) among all genes in the analysis. Light color represents low overlap, and progressively darker red color represents higher overlap. Blocks of darker colors along the diagonal are the modules. (**G**) Interaction network between lncRNAs and miRNAs involved in CAD; yellow highlighted nodes show interaction between *SPANXA2-OT1* and *miR-338*. (**H** and **I**) Violin plot representation of raw intensity expression of *SPANXA2-OT1* (**H**) and *IL-8* (**I**) from dataset GSE113079. The plot includes expression data from *n* = 48 individuals acting as controls and *n* = 93 patients with CAD. (**J**) Violin plot representation of raw intensity expression of miRNA-338 across GSE59421 and GSE105449. The plot includes expression data from *n* = 79 individuals acting as controls and *n* = 71 patients with CAD. Statistical analysis was performed using Welch’s *t* test. ****P < 0.0001.

To screen differentially expressed genes (DEGs) in patients with CAD, we searched for high-throughput transcriptomics data (Figure 1A). After an extensive search, we selected 8 studies (accession GSE56885, GSE98583, GSE42148, GSE34822, GSE90074, GSE974, GSE113079, and GSE28829) for mRNA, 2 studies (accession GSE59421 and GSE105449) for miRNA meta-analysis, and 1 study for lncRNA (GSE113079). All the datasets were generated using common microarray platforms, i.e., Agilent and Affymetrix series. The study, GSE113079, was used for WGCNA coexpression analysis between two different types of RNAs, mRNA and lncRNA. One of the major advantages of gene expression meta-analysis is increased statistical power, which can lead to more reliable gene signatures. Our strategy resulted in comparing *n* = 247 human samples from individuals acting as controls and *n* = 356 samples from patients with CAD for target discovery (Figure 1B).

### Differentially expressed mRNAs related to CAD are involved in immune response and chemokine signaling.

From a microarray-based meta-analysis of mRNA, we identified 2,332 DEGs based on the combined T-statistic values and meta-analysis combined *P* value threshold of < 0.05 (Supplemental Data File 1; supplemental material available online with this article; https://doi.org/10.1172/jci.insight.191274DS1). Among all DEGs, expression levels of 1,235 genes were significantly higher and expression levels of 1,097 genes were lower in CAD samples compared with control samples, respectively. As demonstrated in Supplemental Table 2, C-X-C motif chemokine ligand 8 (*CXCL8*), a major mediator of inflammatory responses by monocyte chemotaxis, more commonly referred to as *IL-8*, was the most increased gene, along with serum/glucocorticoid regulated kinase 1 (*SGK1*) and G0/G1 switch 2 (*G0S2*). *IL-8* has become a component of our candidate regulatory triad in the present study, as demonstrated later. The KEGG enrichment of DEGs from our analysis resulted in chemokine signaling being the top enriched pathway (Figure 1C). GSEA of chemokine signaling demonstrated higher expression of the chemokine signature in patients with CAD compared with individuals acting as controls (Figure 1D). Supplemental Figure 1A shows a heatmap of expression profiles for the top 40 differentially expressed mRNAs obtained from meta-analysis. The contribution of DEGs from each dataset to the DEGs coming from meta-analysis is represented by a chord diagram (Supplemental Figure 1B). The enrichment network of shared DEGs based on biological processes resulted in immune related pathway cluster (Supplemental Figure 1, C and D).

### Differentially expressed miRNAs are involved in lipid metabolism and macrophage activation.

To identify miRNA signatures in the CAD condition, we selected two microarray studies for meta-analysis. We separately normalized the raw intensities and identified differentially expressed miRNAs (DEMs) in the 2 selected datasets from the Gene Expression Omnibus (GEO). We found 468 DEMs in GSE59421 and 461 DEMs in GSE105449. Then, we performed a meta-analysis of these 2 datasets using INMEX (https://www.networkanalyst.ca/) by combining the adjusted *P* values (Fisher’s method), which resulted in 159 DEMs (Supplemental Data File 2). Top DEMs from meta-analysis are represented in the heatmap (Supplemental Figure 2B). Supplemental Figure 2, C and D, shows the DEMs identified by the individual datasets and meta-DEMs. *hsa-miR-1246* (combined T-statistic = 55.0 and combined *P* = 1.88 × 10^–5^), *hsa-miR-598* (combined T-statistic = 55.0 and combined *P* = 1.88 × 10^–5^), and *hsa-miR-199a-5p* (combined T-statistic = 128.6 and combined *P* = 0) were the most increased miRNAs across the two microarray datasets, while *hsa-miR-376c* (combined T-statistic = 128.6 and combined *P* = 0), *hsa-miR-543* (combined T-statistic = 128.6 and combined *P* = 0), and *hsa-miR-1* (combined T-statistic = –52.6 and combined *P* = 3.82 × 10^–5^) were the most decreased miRNAs, as shown in Supplemental Table 3. In addition, *has-miR-338*, one of the components of the candidate regulatory triad, was also among the top decreased DEMs. We performed overrepresentation analysis of the DEMs using the miRNA enrichment analysis and annotation tool (miEAA; https://ccb-compute2.cs.uni-saarland.de/mieaa/). This analysis resulted in the enrichment of miRNAs to several pathways associated with atherosclerosis development, such as “blood vessel remodeling,” “lipid binding,” “lipid storage,” and “macrophage activation” based on the miRWalk database (Supplemental Table 4).

### Landscape of lncRNAs in CAD and functional enrichment of DELs reveals its association with PDGF and IL signaling pathways.

Using the GSE113079 dataset, we conducted a differential expression analysis of lncRNAs in CAD. This dataset contains transcriptome-wide profiling of lncRNAs in PBMCs derived from a well-characterized human cohort comprising 93 patients with CAD and 48 individuals acting as healthy controls. This dataset enabled us to assess the expression of *SPANXA2-OT1* in a clinically relevant population. Our targeted analysis revealed detectable expression of *SPANXA2-OT1* in immune-enriched PBMCs and identified its disease-associated regulation. These findings support the relevance of *SPANXA2-OT1* in human immune cells and provide a rationale for our subsequent mechanistic studies in macrophages. The dataset had more than 6,000 known lncRNA probes captured. After preprocessing and normalization of the raw data, we identified 515 DELs between control and CAD samples (Supplemental Data File 3). DELs from our analysis fall into different classifications of antisense lncRNAs, pseudogene lncRNAs, and uncharacterized lncRNAs. Among them, 133 and 382 genes were increased or decreased DELs, respectively. Supplemental Figure 3B shows the expression pattern of the top 50 DELs across the samples. Sample size distribution for DELs analysis is shown in Supplemental Figure 3C. Supplemental Figure 3D shows a volcano plot of significant DELs; *SPANXA2-OT1* was highlighted as one of the increased DELs in our analysis. Supplemental Table 5 includes the top 10 annotated lncRNAs from our analysis. *GS1-204I12.1*, a long intergenic noncoding RNA was the most increased (log fold change = 1.65 and adjusted *P* = 1.79 × 10^–18^), while *CTD-2532D12.4*, an uncharacterized lncRNA (log fold change = –1.84 and adjusted *P* = 4.49 × 10^–26^) was the most decreased lncRNA.

Since lncRNAs do not code for proteins analyzing their functions is challenging. Transcription factor enrichment analysis is one way to functionally annotate DELs. Transcriptional regulation is a crucial process for controlling the expression of genes, including lncRNA, in pathophysiological events such as CAD. Using the AnnoLnc tool (https://annolnc.gao-lab.org/), we found the overrepresented list of conserved transcription factor binding sites in the DELs. Pathway analysis of the overrepresented transcription factors associated with DELs revealed significant enrichment of several pathways related to inflammation. These results may indicate the role of CAD-related DELs in the pathophysiology of CAD. Pathways such as PDGF as well as IL and CCKR signaling were significantly enriched with the transcription factors associated with our DELs (Supplemental Figure 4).

### CAD-related lncRNA-mRNA coexpression network analysis resulting in 26 coding-noncoding network modules.

We performed sample clustering based on euclidean distance using WGCNA algorithm to remove the outliers, and it resulted in removal of 5 outlier samples from the GSE113079 dataset (Supplemental Figure 5B). Applying the soft threshold power of 6, we acquired 26 gene modules. A minimum module size of 30 and a medium sensitivity (deep split = 2) were utilized to segment the clusters (Figure 1E). We visualized the gene network using a heatmap plot depicting the topological overlap matrix among all lncRNAs and mRNAs in the analysis (Figure 1F). Our results revealed that the module 1–related network (Supplemental Table 6) consisted of 6 lncRNAs and 325 mRNAs, the module 2–related network consisted of 12 lncRNAs and 296 mRNAs, the module 3–related network consisted of 15 lncRNAs and 252 mRNAs, and the module 4–related network consisted of 5 lncRNAs and 192 mRNAs (Supplemental Table 6). We performed scale independence (Supplemental Figure 5C) and mean connectivity (Supplemental Figure 5D) to create a network that is both statistically sound and biologically interpretable, allowing us to identify key gene modules and potential regulatory mechanisms.

### SPANXA2-OT1 is the hub lncRNA in the blue module of the WGCNA network.

From the 26 coding-noncoding coexpression modules extracted from our WGCNA analysis (Supplemental Table 6), we focused on the blue module, which contained 6 lncRNAs and 325 mRNAs. This module was selected because it included the highest number of significantly DEGs. Within this module, we constructed a network using an edge-weight cutoff to identify hub lncRNAs. *SPANXA2-OT1* emerged as the top hub lncRNA based on its highest intramodular connectivity, i.e., the maximum number of coexpression edges with protein-coding genes. *SPANXA2-OT1* was also the most significantly upregulated lncRNA in the module, with a log_2_ fold change of 0.73967 and an adjusted *P* value of 6.73 × 10^–11^.The coding potential analysis and active protein domain analysis using various tools suggested *SPANXA2-OT1* to be non-coding in nature (Supplemental Table 7). Our analysis of potential ORFs associated with *SPANXA2-OT1* demonstrated that 8 of the 9 ORFs displayed strong noncoding scores, consistent with noncoding RNA. However, ORF5 exhibited a marginally coding score, suggesting a borderline or weak coding potential. This observation prompted further investigation, and we found that ORF5 lacks known protein domains when assessed via Pfam and NCBI BLASTP, indicating no homology to any annotated human protein (Supplemental Table 8). Based on these findings, we selected *SPANXA2-OT1* as the candidate lncRNA for the regulatory triad in CAD. *SPANXA2-OT1* is a lncRNA with located on chromosome X: 141,177,284-141,649,927, with 6 isoforms according to the latest annotation provided by the Ensembl database (Ensembl Gene ID, ENSG00000277215). In this study, we focused on the isoform, ENST00000622372.1, based on the analysis of microarray data, as the probe ID on the microarray platform corresponds to the ENST00000622372.1 (NR_037183) isoform of *SPANXA2-OT1*. We designed the primers for this isoform on NCBI, as it has the validated RNA sequence (NR_037183.2) for this isoform. Supplemental Figures 8 and 9 present CAD-related lncRNA-mRNA coexpression networks and their enrichment analysis.

### LncRNA-SPANXA2-OT1 regulates key CAD gene IL-8 via miRNA sponging.

LncRNAs are known to bind miRNAs via seed sequence that is present on lncRNAs for particular miRNA, leading to sponging. This miRNA sponging by lncRNAs eventually leads to the regulation of mRNAs downstream of the sponged miRNA (16, 21). To search for lncRNAs functioning as miRNA sponges, we conducted putative miRNA analysis to find mature human miRNAs associated with DELs. The results identified 354 interactions between lncRNA and miRNA. These interactions enabled the construction of an lncRNA-miRNA network comprising lncRNAs and miRNAs as two types of nodes and edges as putative interactions (Figure 1G). Using this network, we identified hsa-miR-338 as one of the top interacting miRNAs based on its degree, defined as the number of predicted lncRNA partners in the lncRNA-miRNA interaction network. A higher degree indicates greater connectivity and suggests that *miR-338* may be a central regulatory node potentially targeted by multiple lncRNAs. Notably, *miR-338* was also one of the top decreased miRNAs from our meta-analysis (combined T-statistic = –11.516 and combined *P* = 0.017736). The secondary structure analysis of *SPANXA2-OT1* demonstrated miRNA binding sites with stable thermodynamic binding with minimum free energy = –24.4 kcal/mol (Supplemental Figure 5E). This analysis hints toward the miRNA sponging between *SPANXA2-OT1* and *miR-338*. Target prediction of the *miR-338* and the fact that *IL-8* is the top decreased gene in our meta-analysis resulted in selection of *IL-8* as the final molecule in the regulatory triad. The selection of *IL-8* was supported by the RNA hybrid analysis demonstrating stable binding between *miR-338*-*IL-8*, with minimum free energy = –22.7 kcal/mol. As mentioned, *IL-8* is the most highly expressed DEG from out mRNA meta-analysis, with combined T-statistic = 102.16 and combined *P* = 2.06 × 10^–11^. The raw expression of triad genes, lncRNA-*SPANXA2-OT1* (Figure 1H) and *IL-8* (Figure 1I) were significantly increased in CAD samples, while the *miR-338* (Figure 1J) was decreased but not significantly. Based on similar approaches, we also tested another regulatory triad comprising *LINC00211-miR214-BAG2/FBLN5*, but validation of the expression pattern of this triad in human primary macrophages failed (Supplemental Figure 10).

### SPANXA2-OT1 is not conserved across species.

Results of the conservation analysis demonstrate that *SPANXA2-OT1* is not conserved across species. We used different approaches to demonstrate that *SPANXA2-OT1* is only conserved in primates. (a) qPCR using human *SPANXA2-OT1* primers on RNA extracted from mouse aorta (*n* = 6 mice) did not show any amplification (Supplemental Figure 11A). (b) PhyloP score for conservation analysis of the *SPANXA2-OT1* in comparison to conserved lncRNA-HOTAIR demonstrated that SPANXA2-OT1 is not conserved. PhyloP is a measure of conservation for every base. The chart in Supplemental Figure 11B demonstrates the mean phyloP scores of exons and promoter regions (upstream 1 kb) in mammals. A positive phyloP score indicates conservation, while a negative score indicates fast-evolving sequence. The results demonstrate that *SPANXA2-OT1* is poorly conserved across the species as compared with *HOTAIR*. (c) Multiple BLAST alignment for conservation analysis demonstrated that *SPANXA2-OT1* is only conserved in primates, while, lncRNA-*HOTAIR*, a well-studied lncRNA is highly conserved across species (Supplemental Figure 12). Based on these results, we performed all validation experiments in a human cell culture system rather than in mice.

### SPANXA2-OT1 is expressed in cytoplasm upon IL-1β stimulation of human primary macrophages.

**Figure 2:**
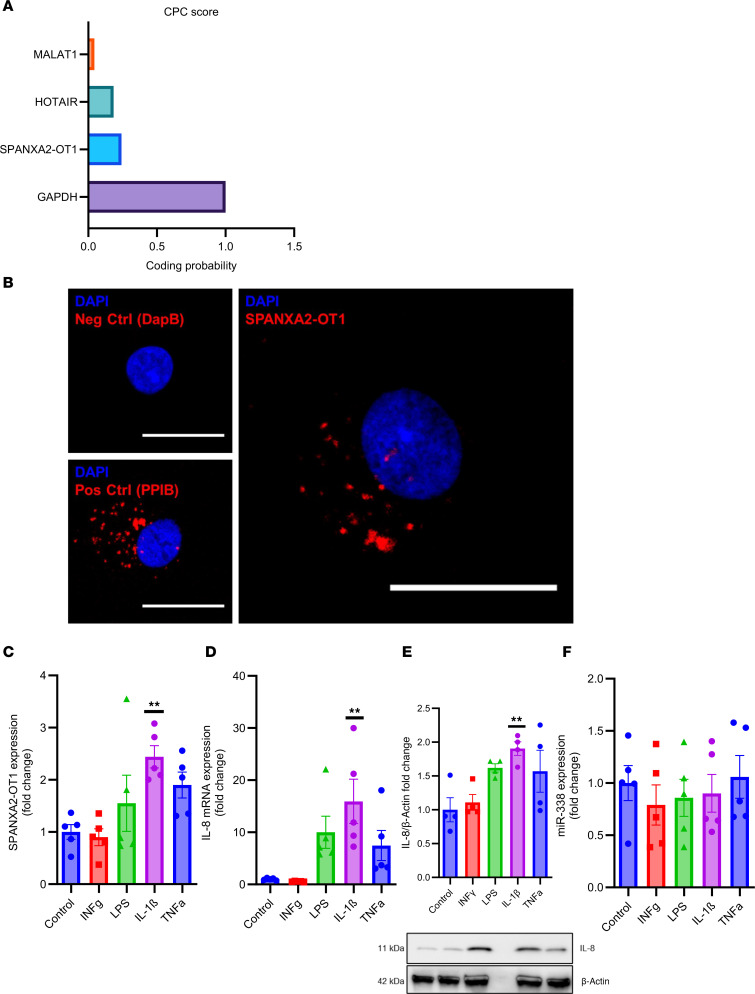
Characterization of lncRNA *SPANXA2-OT1* and its target gene in human primary macrophage cells. (**A**) Coding potential assessing tool predicts very low coding potential for lncRNA-*SPANXA2-OT1*. (**B**) RNA FISH on primary human macrophages demonstrates higher expression of *SPANXA2-OT1* in cytoplasm. Scale bar: 25 μm. (**C**) *SPANXA2-OT1* mRNA expression measured by RT-PCR after inducing with inflammatory stimulus. *n* = 5 PBMC donors. (**D**) *IL-8* mRNA expression measured by RT-PCR after inducing with inflammatory stimulus. *n* = 5 PBMC donors. (**E**) IL-8 protein expression measured by Western blot after inducing with inflammatory stimulus. *n* = 4 PBMC donors. (**F**) *miR-338* expression measured by RT-PCR after inducing with inflammatory stimulus. *n* = 5 PBMC donors. Statistical analysis was performed using 1-way ANOVA with Tukey’s multiple comparisons test. **P < 0.001.

Next, we performed functional characterization of *SPANXA2-OT1*. Coding potential analysis of *SPANAX2-OT1* demonstrated that it has low coding potential, confirming it as a noncoding RNA (Figure 2A). RNA in situ hybridization of *SPANXA2-OT1* demonstrated that it is highly expressed in the cytoplasm of primary macrophages, a localization important for *miR-338* sponging (Figure 2B). To further substantiate the candidate regulatory triad of *SPANXA2-OT1*-*miR-338*-*IL-8*, we stimulated human primary macrophages, derived from PBMCs, with proinflammatory molecules, including TNF-α, INF-γ, LPS, and IL-1β. Among several proinflammatory stimuli tested, only IL-1β significantly increased the mRNA expression of *SPANXA2-OT1* (Figure 2C). The stimulation with IL-1β produced statistically significant increases in the expression of *IL-8* in human primary macrophages both at the mRNA level (Figure 2D) and the protein level (Figure 2E). However, the expression of *miR-338* did not change (Figure 2F), which can be attributed to the fact that lncRNAs act as sponges by binding to miRNAs and preventing them from interacting with their target mRNAs. This sequestration does not necessarily change the miRNA levels but rather affects their availability and activity. Absolute quantification using qPCR revealed that *SPANXA2-OT1*, although expressed at lower basal levels than *miR-338*, exhibited a significant increase in copy number upon IL-1β stimulation in human primary macrophages (Supplemental Figure 13). Despite its moderate expression, the IL-1β–inducible upregulation of *SPANXA2-OT1* supports its context-specific regulatory potential. Prior studies suggest that competing endogenous RNA function depends not solely on transcript abundance, but also on dynamic factors such as miRNA binding affinity, subcellular localization, and competition within localized RNA pools (22, 23). The observed increase in *SPANXA2-OT1* copy number under inflammatory conditions strengthens the hypothesis that it may act as a conditional *miR-338* sponge, potentially modulating downstream targets like *IL-8*. Clinical evidence has linked IL-1β with atherosclerosis through activation of macrophages (24). Here, we identified *SPANXA2-OT1*-*miR-338*-*IL-8* from the microarray datasets generated on PBMCs. Therefore, we selected an in vitro system involving primary human macrophages with IL-1β activation as our model for mechanistic/validation experiments. We quantified the expression of *SPANXA2-OT1* in other cell types including THP-1 macrophages, THP-1 monocytes, Jurkat (T cells), and BLaER1 (B cell) cells; *SPANXA2-OT1* was only significantly (*P* = 0.0266) increased in THP-1–derived macrophages after IL-1β stimulation (Supplemental Figure 14).

### SPANXA2-OT1 and IL-8 have binding sites for miR-338, and SPANXA2-OT1 regulates IL-8 expression by miR-338 sponging.

**Figure 3:**
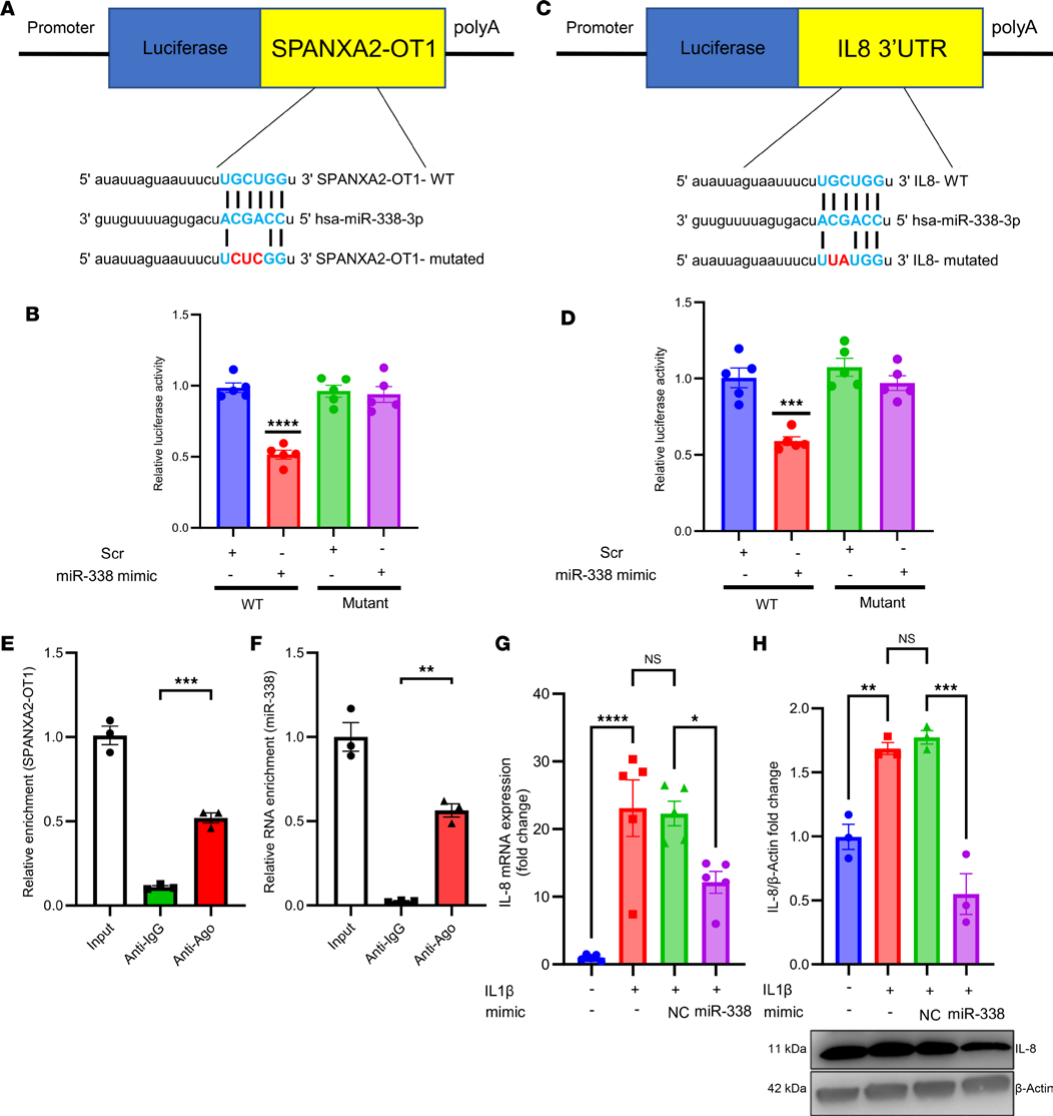
*IL-8* is a target of *miR-338* and *SPANXA2-OT1* indirectly regulates *IL-8* expression by sponging *miR-338*. (**A**) WT and mutant construct sequence for binding site of *miR-338* on *SPANXA2-OT1*. (**B**) Results from dual-luciferase reporter assay. The relative luciferase activity significantly reduced in the presence of *miR-338* mimic in WT construct but remained constant in mutant construct, confirming the binding between *SPANXA2-OT1* and *miR-338*. *n* = 5 replicates. (**C**) WT and mutant construct sequence for binding site of *miR-338* on *IL-8*. (**D**) Results from dual-luciferase reporter assay. The relative luciferase activity was significantly reduced in the presence of *miR-338* mimic in WT construct but remained constant in mutant construct confirming the binding between *IL-8* and *miR-338*. *n* = 5 replicates/group. (**E** and **F**) Ago2-RIP assay shows the enrichment of *SPANXA2-OT1* (**E**) and *miR-338* (**F**). *n* = 3/group. (**G** and **H**) mRNA (**G**) and protein (**H**) levels of IL-8 were significantly reduced in the presence of *miR-338* mimic. Statistical analysis was performed using 1-way ANOVA with Tukey’s multiple comparisons test. ****P < 0.0001; ***P < 0.0005; **P < 0.001; *P < 0.05.

In silico analysis using the RNAhybrid tool (http://bibiserv.techfak.uni-bielefeld.de/rnahybrid) revealed putative binding sites by which *miR-338*-*SPANXA2-OT1* and *miR-338*-*IL-8* have abilities to associate each other. We designed WT and mutant constructs on the *miR-338* binding sites for *SPANXA2-OT1* and *IL-8* (Figure 3, A and C) to confirm the binding in vitro using THP-1–derived macrophages. Dual-luciferase reporter assay analysis showed that *miR-338* mimic reduced the luciferase activities of the *SPANXA2-OT1*-WT and *IL-8*-WT reporter vector but not mutant reporter vectors (Figure 3, B and D), indicating the interaction between *miR-338*-*SPANXA2-OT1* and *miR-338*-*IL-8* requires the putative binding sites identified in silico. Furthermore, an RNA immunoprecipitation (RIP) assay demonstrated a significant enrichment of *SPANXA2-OT1* (Figure 3E) and *miR-338* (Figure 3F) in association with AGO2 (argonaute-2 protein; important for miRNA function), compared with the control (anti-IgG antibody). The RIP-PCR results further verify the binding and eventual sponge formation between *miR-338* and *SPANXA2-OT1*, resulting in elevated *IL-8* expression. To substantiate that *SPANXA2-OT1* colocalizes with AGO2 and P-body marker DCP1a, we performed RNA-protein colocalization in THP-1–derived macrophages using the RNAscope platform (https://acdbio.com/). Merged images revealed partial colocalization of SPANXA2-OT1 with AGO2, a core RISC component, and DCP1a, a marker of P-bodies (Supplemental Figure 15). These findings suggest that *SPANXA2-OT1* may associate with miRNA machinery and RNA degradation foci, consistent with a potential role in posttranscriptional gene regulation. Although single-molecule resolution was not achieved, the observed spatial overlap supports the presence of *SPANXA2-OT1* within RNA-processing compartments.

### miR-338 mimic decreases IL-8 expression in IL-1β–stimulated human primary macrophages.

To further address the potential biological roles *miR-338* in regulation of *IL-8* expression, we delivered *miR-338* mimic in IL-1β–stimulated human primary macrophages. *miR-338* mimic added to human primary macrophages 24 hours before 10 ng/ml IL-1β treatment decreased *IL-8* expression in mimic group as compared with the scramble mimic both at the mRNA level (Figure 3G) and protein level (Figure 3H). These results substantiate that *miR-338*, which binds to *IL-8* (as demonstrated by luciferase assay), also regulates its expression.

### Global transcriptomics and proteomics of SPANXA2-OT1–silenced human primary macrophages resulted in enrichment of chemotaxis signatures.

**Figure 4:**
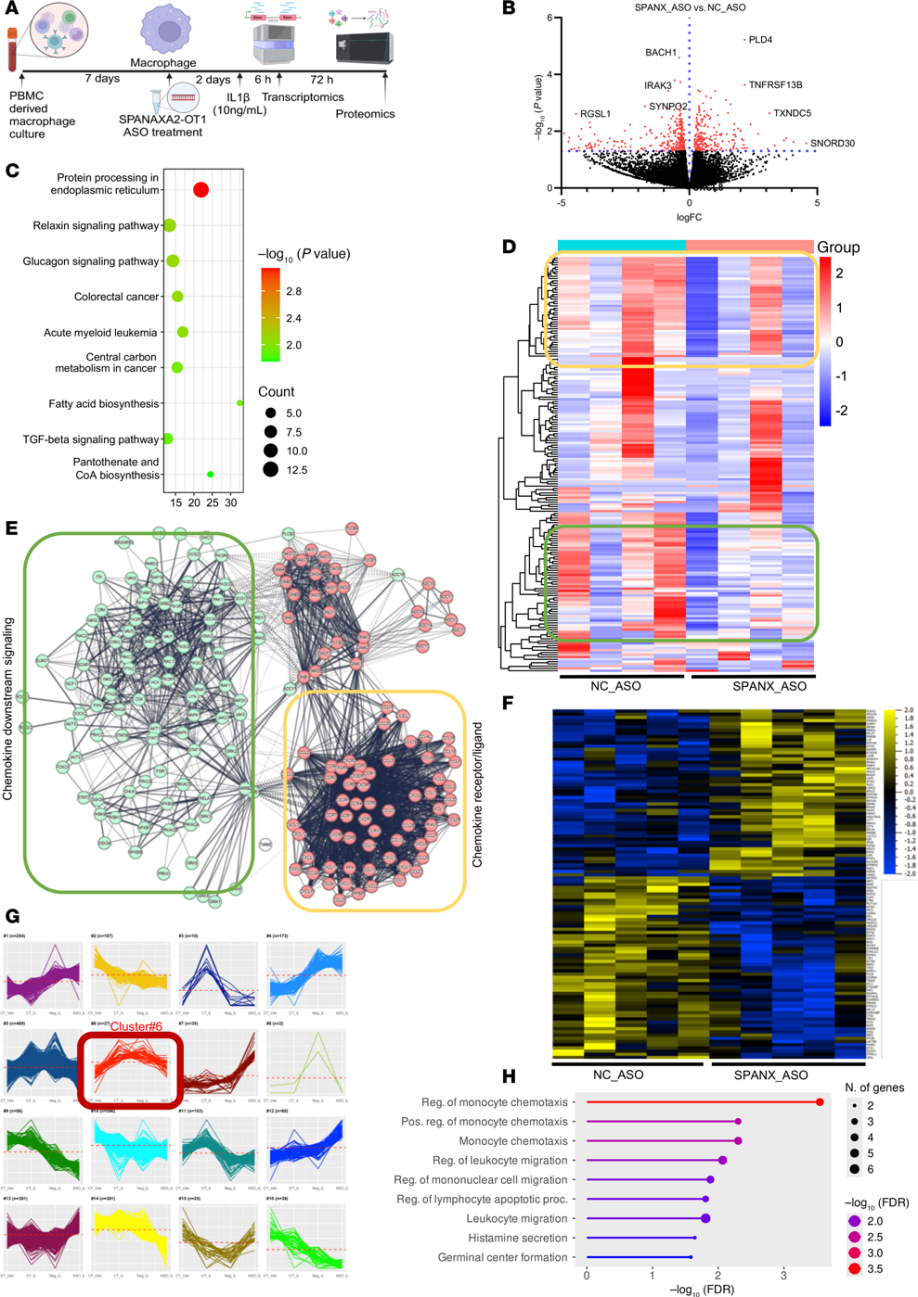
Global transcriptomics and proteomics of *SPANXA2-OT1*–silenced human primary macrophages found in enrichment of chemotaxis signatures. (**A**) Schematics of global omics on *SPANXA2-OT1–*silenced human primary macrophages. (**B**–**E**) RNA-Seq data. *n* = 4 PBMC donors. (**B**) Volcano plot, in which red dots denote the statistically significant overexpressed and underexpressed mRNAs (adjusted *P* value and log fold change), while the gray dots denote statistically nonsignificant mRNAs. Scr_ASO (scrambled) and SPAN_ASO (SPANXA2-OT1). (**C**) Bubble plot of KEGG pathway enrichment analysis of DEGs; *x* axis represents enrichment score. Count indicates the number of DEGs enriched in pathway; *P* values were corrected by Benjamini-Hochberg method. (**D**) Heatmap representation of DEGs. Clustering analysis resulted in 2 important clusters that have decreased gene expression in *SPANXA2-OT1–*silenced group (yellow and green boxes). (**E**) The clusters were used for STRING database protein-protein interaction (PPI) and pathway analysis and resulted in enrichment of chemokine receptor/ligand (yellow cluster) and chemokine downstream signaling (green cluster), both of which were decreased after *SPANXA2-OT1* silencing in human primary macrophages. (**F**–**H**) Proteomics data. *n* = 5 PBMC donors. (**F**) Heatmap representing the unbiased global proteomics changes after silencing *SPANXA2-OT1* in human primary macrophages. Groups included negative control anti-sense oligo– (NC_ASO–) and *SPANXA2-OT1* anti-sense oligo–treated (SPAN_ASO–treated) macrophages. (**G**) Protein abundance clusters showing sum-normalized quantified data on the *y* axis and macrophage treatment groups on the *x* axis (average of 5 donors/group). Cluster#6 (*n* = 37 proteins, highlighted in red) was selected as the important cluster based on the pattern of expression, in which the expression of proteins increased with IL-1β treatment and was maintained in IL-1β+NC ASO, while the expression decreased in IL-1β+*SPANXA2-OT1* ASO. (**H**) Gene ontology biological process analysis of cluster#6 resulted in enrichment of pathways related to monocyte chemotaxis.

Figure 4A shows the workflow of multi-omic experiments. Silencing of *SPANXA2-OT1* in human primary macrophages using antisense oligonucleotides (ASOs) resulted in a significant reduction in *SPANXA2-OT1* expression (*n* = 5 PBMC donors), without affecting the expression of its overlapping gene *SPANXA2* or the nearby gene *SPANXN1* (Supplemental Figure 16). To confirm that our targeting strategy did not inadvertently alter the expression of adjacent protein-coding genes, we performed qPCR assays on a panel of neighboring and overlapping genes within the *SPANX* gene cluster — including *SPANXA2*, *SPANXN1*, and the housekeeping gene *GAPDH* as a control. No off-target transcriptional effects were observed, supporting the specificity of *SPANXA2-OT1* knockdown. We performed global RNA-Seq on *SPANXA2-OT1–*silenced human primary macrophages, and it resulted in 540 DEGs (*P* < 0.05). Among all DEGs, 239 genes were significantly increased, and 301 genes were significantly decreased in the *SPANXA2-OT1*–silenced group compared with the negative control group. The volcano plot in Figure 4B shows the top 5 increased and decreased DEGs from our analysis. KEGG pathway enrichment analysis of the significantly expressed DEGs resulted in enrichment of pathways related to lipid and protein metabolic pathways (Figure 4C). Clustering analysis of DEGs resulted in identification of 2 important clusters that have decreased gene expression in the *SPANXA2-OT1–*silenced group (yellow and green boxes, Figure 4D). When these clusters were used for STRING database protein-protein interaction (PPI) and pathway analysis, and it resulted in enrichment of chemokine receptor/ligand (yellow cluster) and chemokine downstream signaling (green cluster), both of which were decreased after *SPANXA2-OT1* silencing in human primary macrophages (Figure 4E).

Global proteomics of the human primary macrophages after IL-1β and antisense oligo treatment resulted in differential enrichment of 768 proteins (*P* < 0.05) in 4 groups (untreated, IL-1β, IL-1β+NC ASO, and IL-1β+*SPANXA2-OT1* ASO). When we calculated the DE proteins between the IL-1β+NC ASO and IL-1β+*SPANXA2-OT1* ASO groups, we found 109 DE proteins, as shown in heatmap (Figure 4F). Clustering the protein abundances using sum-normalized quantified data across the 4 groups resulted in selection of cluster#6 based on the pattern of expression, where the expression of proteins increases with IL-1β treatment and is maintained in IL-1β+NC ASO while the expression decreases in IL-1β+*SPANXA2-OT1* ASO (Figure 4G). Enrichment analysis of protein cluster#6 resulted in chemokine signaling and monocyte chemotaxis (Figure 4H), which has proteins that decreased in *SPANXA2-OT1–*silenced group. Our global omics data reinforced the decreased chemokine signatures after *SPANXA2-OT1* silencing, suggesting its role in macrophage chemotaxis.

### RNA interference of SPANXA2-OT1 expression decreases IL-8 expression in IL-1β–stimulated human primary macrophages.

**Figure 5:**
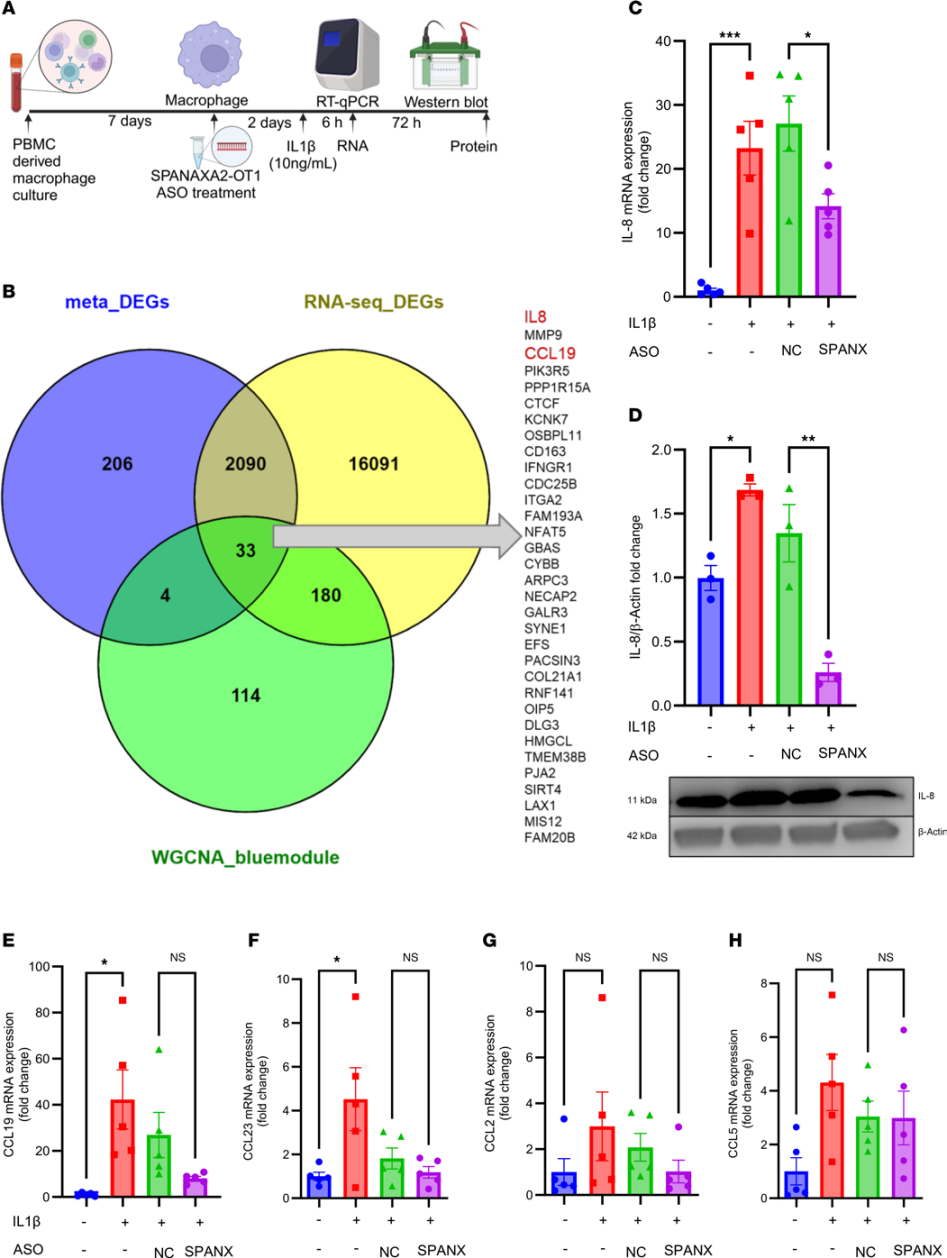
*SPANXA2-OT1* silencing resulted in decreased expression of *IL-8*. (**A**) Schematics of RNA and protein expression in *SPANXA2-OT1–*silenced human primary macrophages. (**B**) Venn diagram showing DEGs from meta-analysis, RNA-Seq on *SPANXA2-OT1–*silenced macrophages, and the blue module from WGCNA analysis, with 33 DEGs being shared, of which *IL-8* and *CCL19* were identified as important chemokines. Silencing of *SPANXA2-OT1* in human primary macrophages resulted in decreased expression of IL-8 at the level of both mRNA (**C**) and protein (**D**). *n* = 5 PBMC donors. (**E**–**H**) mRNA expression of other chemokine signature genes (*CCL19*, *CCL23*, *CCL2*, and *CCL5*) did not change after *SPANXA2-OT1* silencing. *n* = 5 PBMC donors. Statistical analysis was performed using 1-way ANOVA with Tukey’s multiple comparisons test. ***P < 0.0001, **P < 0.001, *P < 0.05.

From our computational analysis, we found 33 DEGs shared among the meta-analysis DEGs, RNA-Seq DEGs from *SPANXA2-OT1*–silenced primary human macrophages, and blue module from WGCNA analysis. The schematic workflow for gene expression studies in *SPANXA2-OT1*–silenced macrophages is presented in Figure 5A. Of these 33 DEGs, *IL-8* and *CCL19* were identified as important chemokines (Figure 5B). To further address the potential biological roles of *SPANXA2-OT1* in regulation of *IL-8* expression, we subsequently used ASO against *SPANXA2-OT1* in IL-1β–stimulated human primary macrophages. Silencing of *SPANXA2-OT1* in human primary macrophages resulted in decreased expression of *IL-8* at both the mRNA level (Figure 5C) and the protein level (Figure 5D). However, the mRNA expression of *CCL19*, another candidate chemokine signature gene, did not change after *SPANXA2-OT1* silencing (Figure 5E). We also quantified the mRNA expression of other chemokines (*CCL23*, *CCL2*, and *CCL5*) known to have role in macrophage chemotaxis, but their expression did not change after silencing *SPANXA2-OT1* (Figure 5, F–H).

### CRISPR/Cas9 deletion of SPANXA2-OT1 functional domain changes macrophage chemokine profile.

**Figure 6:**
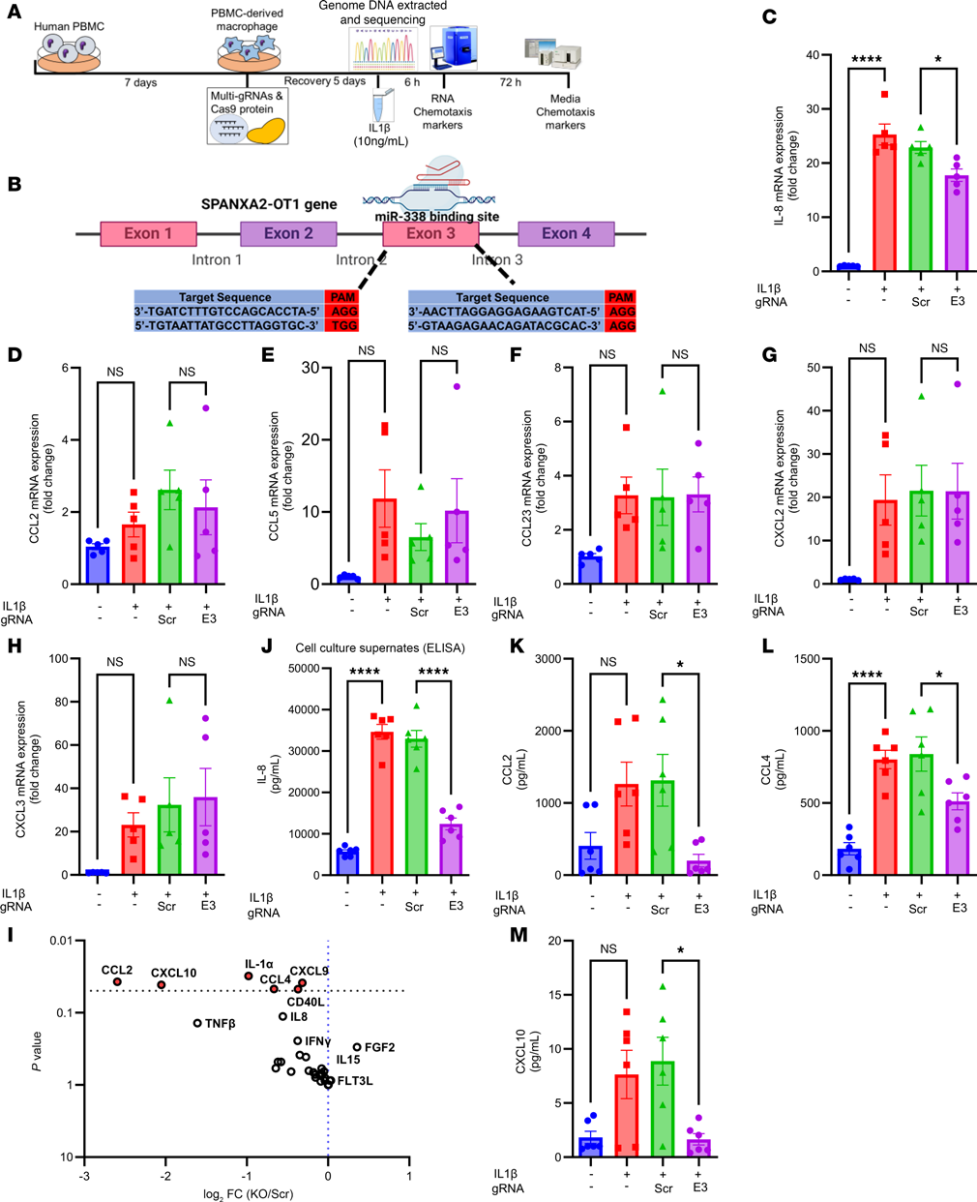
CRISPR/Cas9 deletion of *SPANXA2-OT1* functional domain changes macrophage chemokine profile. (**A**) Schematic of the CRISPR/Cas9 deletion of *SPANXA2-OT1* functional domain (exon 3) experiment. (**B**) Representation of the *SPANXA2-OT1* functional domain, including exon 3, which harbors the binding site for *miR-338*. Using multiple guide RNA, exon 3 was deleted from *SPANXA2-OT1* in human primary macrophages. (**C**) mRNA expression of *IL-8* was significantly reduced after exon 3 deletion. *n* = 5 PBMC donors. (**D**–**H**) mRNA expression of other important chemokines, including *CCL2*, *CCL5*, *CCL23*, *CXCL2*, and *CXCL3*, did not change after exon 3 deletion. *n* = 5 PBMC donors. (**I**–**M**) Chemokine protein profile of media supernatant from *SPANXA2-OT1*–exon 3–deleted human primary macrophages. *n* = 5 PBMC donors. (**I**) Volcano plot representation of significantly reduced chemokines after exon 3 deletion. (**J**) IL-8 ELISA quantification in media supernatant from SPANXA2-OT1–exon 3–deleted human primary macrophages. *n* = 5 PBMC donors analysis revealed a statistically significant reduction in IL-8 protein levels following exon 3 deletion of SPANXA2-OT1. (**K**–**M**) Protein levels of CCL2 (**K**), CCL4 (**L**), and CXCL10 (**M**) were measured by multiplex assay and were significantly reduced after exon 3 deletion. Statistical analysis was performed using 1-way ANOVA with Tukey’s multiple comparisons test. *P < 0.05; ****P < 0.0001.

Methodological details are depicted in the schematic of Figure 6A. Figure 6B depicts the target sequence and guide RNA sequence for deletion of exon 3 on *SPANXA2-OT1* gene. The CRISPR/Cas9-mediated deletion of the *SPANXA2-OT1* functional domain (exon 3) substantially altered the chemokine profile of human primary macrophages. The deletion was achieved using multiple guide RNAs targeting exon 3, which harbors the binding site for *miR-338*. This resulted in a significant reduction in mRNA expression of *IL-8* (Figure 6C). However, the mRNA levels of other key chemokines, including *CCL2*, *CCL5*, *CCL23*, *CXCL2*, and *CXCL3*, remained unchanged (Figure 6, D–H). Analysis of the chemokine protein profile in the media supernatant from the *SPANXA2-OT1* exon 3–deleted macrophages revealed a marked decrease in the protein levels of CCL2, CCL4, and CXCL10, as depicted in the volcano plot (Figure 6I) and protein quantification data (Figure 6, K–M). We performed a targeted ELISA to quantify IL-8 secretion in human primary macrophages following *SPANXA2-OT1* exon 3 deletion. IL-8 protein levels were significantly reduced compared with control macrophages (*n* = 5 donors), indicating that *SPANXA2-OT1* knockdown functionally suppresses IL-8 secretion. This result reinforces our proposed miRNA sponge mechanism and addresses the sensitivity limitations of the initial multiplex assay (Figure 6J). These findings underscore the specific effect of *SPANXA2-OT1* exon 3 deletion on the chemokine secretion profile of macrophages, highlighting its potential role in modulating immune responses.

### SPANXA2-OT1 plays a role in macrophage chemotaxis.

**Figure 7:**
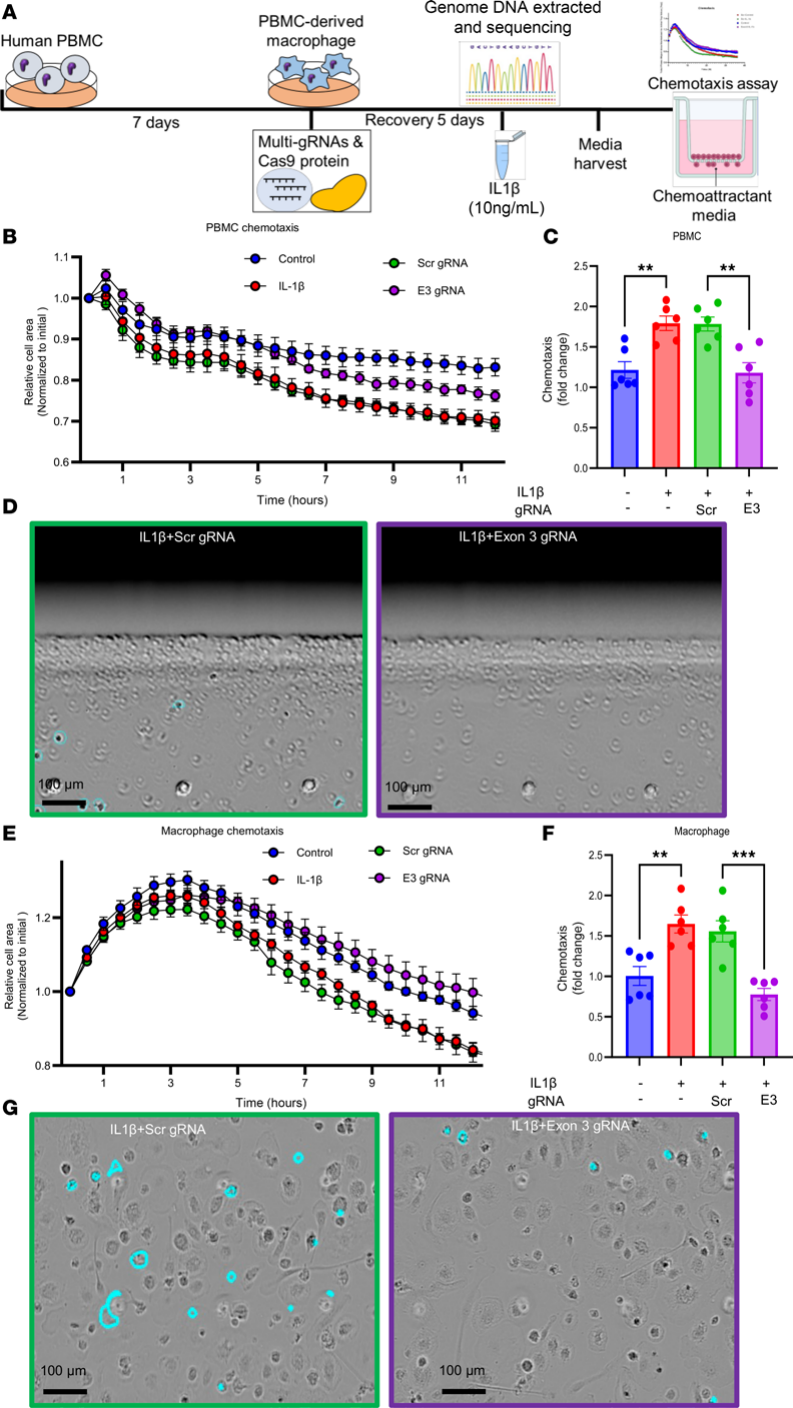
*SPANXA2-OT1* plays a role in macrophage chemotaxis. Chemotaxis assay of human PBMC and macrophages with *SPANXA2-OT1* (Exon 3) knockout macrophage supernatant media as chemoattractant. (**A**) Schematic of CRISPR/Cas9 editing of *SPANXA2-OT1*–exon 3 and chemotaxis experiment. (**B** and **C**) Live-cell imaging of PBMC chemotaxis for 12 hours. PBMC chemotaxis was significantly reduced in exon 3–deleted group. *n* = 6 PBMC donors. (**D**) Image representation of PBMC chemotaxis after 6 hours. The cells marked with turquoise are the cells that moved to bottom of the matrix gel and were counted as chemotaxis cells. Scale bar: 100 μm. (**E** and **F**) Live-cell imaging of primary macrophage chemotaxis for 12 hours. Macrophage chemotaxis significantly reduced in exon 3–deleted group. *n* = 6 PBMC donors. (**G**) Image representation of macrophage chemotaxis after 6 hours. The cells marked with turquoise are the cells that moved to bottom of the matrix gel and were counted as chemotaxis cells. Statistical analysis was performed using 1-way ANOVA with Tukey’s multiple comparisons test. ***P < 0.0001, **P < 0.001. Scale bar: 100 μm.

We investigated the role of *SPANXA2-OT1* in macrophage chemotaxis using CRISPR/Cas9-mediated deletion of exon 3 of *SPANXA2-OT1* in primary human macrophages. Schematics for chemotaxis assay followed by CRISPR/Cas9 deletion of SPANXA2-OT1–exon 3 are presented in Figure 7A. The chemotactic behavior of PBMCs and macrophages was evaluated using a live-cell imaging system over 12 hours, with supernatant media from exon 3–deleted macrophages serving as the chemoattractant. Our results show a significant reduction in PBMC migration in the *SPANXA2-OT1* exon 3–knockout group compared with the control group (Figure 7, B and C), as quantified by the number of cells traversing to the bottom of the matrix gel (Figure 7D). Similarly, primary macrophage chemotaxis was significantly impaired in the exon 3–deleted group, as observed in both live-cell imaging (Figure 7, E and F) and the 6-hour migration endpoint (Figure 7G), where turquoise-marked cells indicated successful migration. These findings highlight a critical role for *SPANXA2-OT1* in promoting macrophage and PBMC chemotaxis, potentially providing insight into its broader role in immune cell recruitment. We investigated chemotaxis of other immune cells (THP-1 macrophages, Jurkat/T cells, and BLaER1/B cells) using the same experimental setup, but we did not find any significant changes in the chemotaxis of these immune cells in media supernatant of *SPANXA2-OT1* exon 3–deleted macrophages serving as the chemoattractant (Supplemental Figure 17). Our data on IL-8 neutralization demonstrated reduced macrophage chemotaxis induced by *SPANXA2-OT1*. As shown in Supplemental Figure 18A, fewer macrophages migrated in response to IL-8–neutralized conditioned media compared with isotype control, as indicated by a reduced number of cells at the gel bottom (white arrows). Supplemental Figure 18B illustrates a time-resolved reduction in chemotaxis over 12 hours in both IL-8–neutralized and *SPANXA2-OT1*–deleted conditioned media. Quantification (Supplemental Figure 18C) revealed a dose-dependent reduction in chemotaxis with IL-8 antibody treatment, reaching statistical significance at 1 μg/mL (*P* < 0.0001). These data demonstrate that IL-8 is a principal chemotactic factor downstream of *SPANXA2-OT1*, and its neutralization phenocopies the impaired migration observed upon *SPANXA2-OT1* exon 3 deletion.

## Discussion

**Figure 8:**
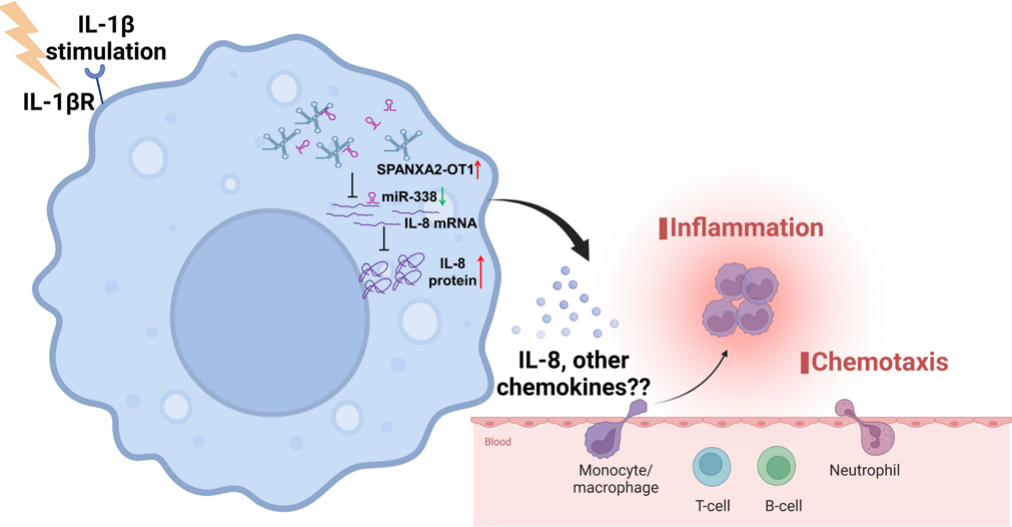
*SPANXA2-OT1* regulates macrophage chemotaxis. Graphical representation of lncRNA-*SPANXA2-OT1* regulating human primary macrophage chemotaxis in part via *SPANXA2-OT1*’s binding to *miR-338* and its regulation of expression of *IL-8* and other chemokines.

In this study, we investigated lncRNA-mediated inflammation in CAD using gene expression meta-analysis and subsequent validation of the candidate in human primary macrophages. The key findings include the following: (a) gene expression meta-analysis of the mRNA datasets in CAD revealed the enrichment of inflammatory pathways; (b) gene expression meta-analysis of the miRNA datasets in CAD indicated that DEMs are involved in lipid metabolism and macrophage activation; (c) unbiased global DEL analysis of the GSE113079 dataset identified a landscape of lncRNA in CAD with 515 DELs; (d) construction of CAD-related lncRNA-mRNA coexpression network resulted in 26 coding-noncoding network modules, with *SPANXA2-OT1* as the top candidate lncRNA; (e) knockdown of *SPANXA2-OT1* and induction of *miR-338* mimic led to the decreased expression of *IL-8* in IL-1β–induced primary macrophages, confirming the role of lncRNA-*SPANXA2-OT1* in inflammation via regulation of *IL-8* expression; (f) global transcriptomics and proteomics of *SPANXA2-OT1–*silenced human primary macrophages resulted in enrichment of chemotaxis signatures; and (g) CRISPR/Cas9 deletion of the *SPANXA2-OT1* functional domain changed the macrophage chemokine profile and decreased macrophage chemotaxis. Based on these findings, we propose a hypothesis regarding the role of *SPANXA2-OT1* in regulation of macrophage chemotaxis; one such mechanism is via the *SPANXA2-OT1*-*miR-338*-*IL-8* regulatory triad in CAD (Figure 8).

CAD is a multifactorial disease with complex pathophysiology. From being considered a cholesterol storage disease to understanding the role of vascular remodeling, CAD research has came a long way in deducing the molecular and cellular events behind this disease. Evidence suggests that inflammation plays a key role in CAD via multiple interrelated immune mechanisms that interact with genetic and metabolic risk factors to initiate, promote, and activate lesions in the coronary arteries (25–27). It is thus important to identify early biomarkers predicting inflammatory mechanisms in CAD for the prevention and treatment of the disease. LncRNAs are important regulatory RNAs because of their roles in several checkpoints of gene expression by interacting with DNA, coding RNA, miRNA, and proteins. LncRNAs are emerging as important regulators of cellular function, and their dysregulation can contribute to human disease (28–30). Little is known, however, about the miRNA sponging role of lncRNAs in the inflammatory mechanisms of CAD. We hypothesized that a lncRNA could interact with a miRNA and subsequently with a mRNA, thus having an indirect effect on protein expression and disease evolution. Our study elucidated the involvement of the *SPANXA2-OT1*-*miR-338*-*IL-8* axis in CAD, specially, its role in inflammation, suggesting that *SPANXA2-OT1* may act as a therapeutic target for aberrant macrophage functions.

By jointly analyzing 8 published microarray gene expression datasets of CAD, we identified 2,332 DEGs, including 1,235 overexpressed and 1,097 underexpressed genes across the datasets, which provided us a robust list of CAD-associated genes by employing the gene expression meta-analysis approach (31). *IL-8*, which eventually became a component of our candidate regulatory triad, was the most increased gene. *IL-8* is an important chemokine released by the proinflammatory macrophages, and it has role in recruitment of immune cells leading to inflammation (32). Evidence suggests the role of *IL-8* in atherosclerosis (33). Our study provides a mechanism of how *IL-8* expression is regulated by the interplay between noncoding RNAs (*SPANXA2-OT1* and *miR-338*) globally in CAD and precisely in human primary macrophages. In addition to the *IL-8* gene, there were several other DE genes in our analysis that contribute to inflammation, as evident from the results of pathway analysis, which shows the enrichment of several pathways, including “chemokine signaling pathway,” “CCKR signaling,” and “Toll-like receptors cascades.” All these pathways are part of the inflammatory bandwagon in CAD (9, 34, 35). A major limitation in meta-analyses of public transcriptomic datasets lies in the inconsistency of sample annotations and potential batch effects across studies. These issues can confound downstream coexpression and differential expression analyses. To mitigate this, we applied ComBat for batch correction, used a uniform annotation reference, and filtered for robustly expressed and consistently annotated genes across datasets. While these steps help standardize the analysis, we acknowledge that residual variability may persist, and careful interpretation is warranted. We recommend future studies incorporate sample-level metadata harmonization and standardized preprocessing pipelines to further improve reproducibility in cross-cohort omics integration.

Accumulating recent evidence suggests that lncRNAs and miRNAs play pivotal regulatory roles in the pathobiology of atherosclerosis (36, 37). LncRNAs can function as miRNA sponges and compete for miRNA binding to protein-coding transcripts (21). Limited studies on the miRNA sponging mechanisms have established efferocytosis of macrophages and proliferation of vascular smooth muscle cells (18, 38). Our study provides a blend of global in silico screening using gene expression datasets and in vitro validation of the candidate miRNA sponge in primary human macrophages to establish the inflammatory mechanism in CAD. *miR-338* was among the top decreased miRNAs from our miRNA gene expression meta-analysis. While a previous study showed the role of *miR-338* in endothelial cell injury through targeting *BAMBI* and activating the TGF-β/Smad pathway (39), our study has established its role in macrophage activation via lncRNA regulation. *SPANXA2-OT1*, a lncRNA and the final component of our candidate regulatory triad, is less studied. Its only known role is in the occurrence and development of epithelial-mesenchymal transition in calcium oxalate crystal-induced kidney injury by adsorbing *miR-204* and upregulating *SMAD5* (40). Our study is the first to our knowledge to find the role of *SPANXA2-OT1* in CAD.

LncRNAs are the RNAs that do not translate into protein, and therefore it is challenging to functionally characterize them. One way to overcome this challenge is the use of coexpression network analysis of lncRNAs and protein-coding genes to understand functional or regulatory relationships (41). We therefore integrated DE mRNAs from the mRNA meta-analysis and DE lncRNAs using WGCNA to determine the correlated modules comprising of lncRNAs and its coding gene partners (42). We selected *SPANXA2-OT1* as our candidate lncRNA based on this analysis, as it was the top module with maximum number of interactions with the coding genes in the module. The constructed coding-noncoding network modules shows that the coding genes associated with DE lncRNAs in CAD are involved in inflammatory signaling and lipid metabolism pathways.

Integration of the findings using a systems approach, employing in silico tools and network biology, has become important to explore the regulatory triad for validation in experimental set up. Our study provides a framework for this complex analysis, in which we seek how lncRNA (*SPANXA2-OT1*) regulates miRNA (*miR-338*) function by acting as endogenous sponges and thus regulates gene expression of its target coding gene (*IL-8*). This study revealed that *IL-8* is a target of *miR-338-5p* using the bioinformatics analysis and dual-luciferase assay, and overexpression of *miR-338-5p* resulted in the decreased expression of *IL-8* at RNA and protein levels. In addition, we observed that *miR-338-5p* was negatively regulated by *SPANXA2-OT1*, while *IL-8* was positively regulated by it, and *SPANXA2-OT1* served as a sponge of *miR-338* to regulate *IL-8* expression in human primary macrophages.

Our investigation into the role of *SPANXA2-OT1* in macrophage chemotaxis using CRISPR/Cas9-mediated deletion of exon 3 has provided important insights into immune cell behavior. The deletion of exon 3 in *SPANXA2-OT1* led to a marked reduction in the chemotactic behavior of both PBMCs and primary macrophages. This was evidenced by a significant decrease in cell migration in live-cell imaging assays and endpoint migration measurements. These findings underscore the critical role of *SPANXA2-OT1* in promoting macrophage and PBMC chemotaxis, suggesting its broader involvement in immune cell recruitment. Supporting literature emphasizes the importance of chemotaxis in immune responses. Chemokines and their receptors are pivotal in directing the movement of immune cells to sites of inflammation or injury (43). For instance, studies have shown that the disruption of specific chemokine pathways can significantly impair immune cell migration and function (44). The observed impairment in macrophage and PBMC chemotaxis upon *SPANXA2-OT1* deletion aligns with previous research highlighting the role of lncRNAs in regulating immune cell behavior (45). These results provide valuable insights into the functional role of *SPANXA2-OT1* in immune cell recruitment and its potential implications for therapeutic strategies targeting immune responses in CAD.

The strengths of our study include (a) the use of the publicly available microarray datasets to prioritize the candidate regulatory triad in CAD (this gene expression meta-analysis approach gives the substantial advantage of having a large number of human samples for better statistical power); (b) detailed analysis workflow that can be used in future studies for other diseases; (c) a comprehensive list of lncRNAs, miRNAs, and mRNAs in CAD that can be further explored in future as possible mechanisms in CAD; (d) the report of 26 lncRNA-mRNA modules in CAD using WGCNA that can be explored in future studies; (e) unbiased global omics of *SPANXA2-OT1–*knockdown macrophages and independent validation of its role in chemokine signature; (f) independent validation of the candidate *SPANXA2-OT1*-*miR-338*-*IL-8* in human primary macrophage system lending additional support to the study’s comprehensive in silico analysis outcome; and (g) CRISPR/Cas9 deletion of *SPANXA2-OT1* functional domain and extensive chemotaxis assay on different cell types to show the role of *SPANXA2-OT1* in macrophage chemotaxis.

Our study addresses a critical gap in understanding the functional roles of primate-specific lncRNAs in immune regulation by presenting a mechanistically defined and experimentally validated example of how a lncRNA modulates macrophage behavior in CAD. Functional annotation of lncRNAs remains inherently challenging; however, we overcame these limitations by integrating large-scale human gene expression meta-analyses, coexpression network biology, and in vitro loss- and gain-of-function studies in primary human macrophages. Through this approach, we identified and characterized a mechanistic axis — *SPANXA2-OT1–miR-338–IL-8*, linking a primate-specific lncRNA to chemokine signaling and macrophage chemotaxis, both key processes in atherogenesis. CRISPR/Cas9-mediated deletion of the *SPANXA2-OT1*’s *miR-338* binding domain and chemotaxis assays in macrophages and PBMCs provided causal evidence for the immunoregulatory role of *SPANXA2-OT1*. In addition, our comprehensive gene expression meta-analysis of differentially expressed mRNAs, miRNAs, and lncRNAs offers a robust catalog of regulatory RNAs implicated in CAD, revealing interactions among lncRNAs, coding genes, miRNAs, and transcription factors that define the disease signature. Specifically, we propose that *SPANXA2-OT1* modulates the chemokine profile of macrophages by acting as a competing endogenous RNA for *miR-338*, thereby relieving its suppressive effect on *IL-8* and promoting *IL-8* expression, which, in turn, may enhance macrophage chemotaxis and inflammatory activation in CAD. These findings provide mechanistic insight into lncRNA-mediated inflammatory regulation and nominate *SPANXA2-OT1* as a potential biomarker and therapeutic target for CAD, warranting further experimental and clinical investigation. Future investigations should extend the current stoichiometric analysis to delineate the precise molecular mechanisms underlying these findings.

## Methods

### Sex as a biological variable.

Sex was not considered as a biological variable.

For detailed experimental methods, please see the Supplemental Methods.

### Statistics.

Differential expression analysis of individual microarray datasets was done by using LIMMA after variance stabilization normalization followed by quantile normalization on INMEX. For gene expression meta-analysis, the differential expression across CAD and control samples was assessed by combining the *P* value (Fisher’s method) using INMEX. For the functional enrichment analysis, significantly enriched terms were corrected using the Bonferroni/Benjamini & Hochberg false discovery rate correction. Data are shown as mean ± SD of at least 3 independent experiments performed with samples from at least 3 different donors and were processed using GraphPad Software version 9.1.0. Differences between groups were evaluated using unpaired 2-tailed Student’s *t* test and 1-way ANOVA followed by the Tukey’s test. A false discovery rate–adjusted *P* value of less than 0.05 was considered statistically significant.

### Study approval.

Animal experiments were approved by the Brigham and Women’s Hospital’s Animal Welfare Assurance (protocol 2016N000219).

### Data availability.

The mass spectrometry proteomics data have been deposited to the ProteomeXchange Consortium via the PRIDE partner repository with the dataset identifier PXD059474. The RNA-Seq data are accessible at GSE307322. Datasets used for gene expression meta-analysis were downloaded from the NCBI GEO public repository, as detailed in Supplemental Table 1.

## Author contributions

PKJ and MA conceived, coordinated, and designed the study. PKJ and AV retrieved the datasets and did the analysis. PKJ, LYUI, AL, SC, YN, TDL, CBN, MW, DH, and DBG performed the experiments. PKJ wrote the manuscript. SAS, TK, and MBC assisted in global proteomics. MA, SU, and EA critically reviewed and edited the manuscript. SU provided technical advices on the study. All authors have reviewed and approved the final manuscript before submission.

## Funding support

This work is the result of NIH funding, in whole or in part, and is subject to the NIH Public Access Policy. Through acceptance of this federal funding, the NIH has been given a right to make the work publicly available in PubMed Central.

- National Heart, Lung, and Blood Institute research grants, R01HL126901 and R01HL149302 to MA and R01HL174066 to MA and EA.

## Supplementary Material

Supplemental data

Supplemental data set 1

Supplemental data set 2

Supplemental data set 3

Unedited blot and gel images

Supporting data values

## Version 1 10/09/2025

In-Press Preview

## Version 2 11/10/2025

Electronic publication
