# Supplementary material for: A systems approach to target discovery identifies the role of lncRNA-SPANXA2-OT1 in macrophage chemotaxis
Source: JCI Insight. 2025 Oct 9;10(21):e191274. doi: 10.1172/jci.insight.191274 (PMC12643516; doi:10.1172/jci.insight.191274)
Supplement: Supplemental data [file jciinsight-10-191274-s231.pdf]

## Supplementary Information

# A systems approach to target discovery identifies the role of lncRNA-SPANXA2-OT1 in macrophage chemotaxis.

Prabhash Kumar Jha[1], Sarvesh Chelvanambi[2], Yuto Nakamura[2], Lucas Yuji Umesaki Itto[1], Aatira Vijay[3], Adrien Lupieri[1], Miguel Cantadori Barbeiro[1], Thanh-Dat Le[1], Caio Borges Nascimento[1], Taku Kasai[2], Mary C Whelan[2], Daiki Hosokawa[2], Dakota Becker-Greene[1], Sasha A. Singh[2], Elena Aikawa[1,2], Shizuka Uchida[4], Masanori Aikawa[1,2,5,*]

[1]Center for Excellence in Vascular Biology, Brigham and Women's Hospital, Harvard Medical School, Boston, USA; [2]Center for Interdisciplinary Cardiovascular Sciences, Brigham and Women's Hospital, Harvard Medical School, Boston, USA; [3]Cardiovascular Research Center, Massachusetts General Hospital, Harvard Medical School, Boston, USA; [4]Center for RNA Medicine, Department of Clinical Medicine, Aalborg University, Copenhagen SV, Denmark; [5]Channing Division of Network Medicine, Brigham and Women's Hospital, Harvard Medical School, Boston, USA

[*]**Correspondence:**

Masanori Aikawa, MD, PhD

Center for Excellence in Vascular Biology

Brigham and Women's Hospital, Harvard Medical School

77 Avenue Louis Pasteur, NRB741, Boston, MA 02115, USA

Email: maikawa@bwh.harvard.edu

## Methods

***Selection of datasets for gene expression meta-analysis***

Public repositories were mined for gene expression datasets related to CAD. We searched for datasets related to mRNA, miRNA and lncRNAs on human samples in PubMed, gene expression omnibus (GEO) and ArrayExpress. We included several inclusion criteria for selection of datasets comprising microarray datasets, human case control study, CAD disease, and untreated samples. We extracted study details for each dataset included in our analysis and detailed it in **Supplementary Table S1**. We conducted this meta-analysis in accordance with the guidelines provided in the Preferred Reporting Items for Systematic Reviews and Meta-Analysis (PRISMA) guidelines (1) that was applied for selection of eligible datasets as described in **Figure 1A**.

***Gene expression meta-analysis of differentially expressed mRNAs and miRNAs and functional enrichment***

We retrieved eight eligible mRNA datasets based on the inclusion criteria detailed in **Figure 1A** for conducting gene expression meta-analysis in CAD. We performed preprocessing, ID conversion, normalization and differential expression analysis of all the datasets individually. For meta-analysis we converted gene probes from all the datasets into common Entrez ID to be comparable during the meta-analysis using a web platform, Integrative Meta-Analysis of Expression Data (INMEX) (2). Gene expression meta-analysis on INMEX was conducted by combining p-values using Fisher's method to identify the most consistent differentially expressed genes across all the eight datasets. One issue associated with meta-analysis is the batch effect generated due to the use of different datasets. Thus, we performed batch effect correction using the ComBat method (3). The list of differentially expressed genes (DEGs) based on combined T-statistic and combined p-value $<0.05$ was identified. We used EnrichR platform to further perform functional enrichment analysis of these DEGs to identify significantly enriched pathways and

biological processes (4). Gene set enrichment analysis was performed to understand the distribution of DEGs in chemokine signaling pathway. For miRNA meta-analysis, we downloaded the raw data of the two selected datasets (GSE59421 and GSE105449) from GEO. We applied the similar analysis approach described in our previous publication (5). The list of differentially expressed miRNAs (DEMs) resulted from our analysis was subjected to over-representation analysis using the miEAA tool (6). miRNA enrichment analysis was conducted to understand the functional association of the DEMs with different biology/pathways. **Supplementary Figure S2A** details the method pipeline for miRNA meta-analysis.

***Differentially expressed lncRNA analysis in CAD and classification pipeline***

Using GSE113079 dataset, we identified differently expressed lncRNAs (DELs) applying the limma package (7). The DELs with adjusted p-value (false discovery rate, FDR) less than 0.05 were selected. In this work, a pipeline was utilized to identify lncRNA expression patterns in CAD, which was described in **Supplementary Figure S3A**. The DELs were matched for known lncRNAs using the NONCODE database (8) and were selected based on the cutoff of adj p-value <0.05 and logFC ≥0.5.

***WGCNA analysis for coding-noncoding network analysis and module detection***

We conducted WGCNA(9) analysis using GSE113079 to understand the potential functions and mechanisms of DELs in CAD. In this study, WGCNA was employed to create a co-expression weighted network between coding (mRNA) and noncoding (lncRNA). After identifying the best parameter (soft threshold = 6), we performed the WGCNA analysis according to the study by Langfelder *et al*[23]. We assessed the weighted co-expression relationship among all samples in an adjacency matrix using the pairwise Pearson correlation. Following the identification of weighted correlation and module identification, the network was presented by Visant tool (10). The pipeline for the analysis of GSE113079 for the identification of the long non-coding landscape

in CAD is described in **Supplementary Figure S3A**.The code related to the analysis along with the data files is deposited on GitHub page (https://github.com/Prabhashkjha/CAD_lncRNA_WGCNA). We used AnnoLnc(11) to identify conserved transcription factor binding sites overrepresented in the promoter regions (−2 kb to +1 kb) of differentially expressed lncRNAs from blue module, suggesting shared regulatory mechanisms.

***Predicting putative lncRNA-miRNA interaction, secondary structure, conservation and coding potential analysis of the candidate lncRNAs***

We further extended the functional analysis of candidate lncRNAs selected from the WGCNA module 1 (blue) based on the number of lncRNA and mRNA interactions. Using TargetScan database (12), we predicted miRNA-binding sites on candidate lncRNAs. A list of putative miRNAs interacting with the candidate lncRNAs was generated and an interaction network was created using Cytoscape to identify the miRNA that binds maximum number of candidate lncRNA. Secondary structure analysis of candidate lncRNA was conducted using the ViennaRNA package (13) and RNAhybrid (14) tool to identify the complementary seed-matching sites and minimum free energy (MFE) of miRNA on the lncRNA. Further, we performed coding potential analysis on the candidate lncRNAs using CPC (15) and PFAM (16) to distinguish coding (mRNA) from noncoding (lncRNA) based on the quality of open reading frame (ORF) and presence or absence of protein domains in the lncRNA sequence. We conducted a comprehensive ORF analysis of the full *SPANXA2-OT1* sequence using the NCBI ORF Finder. This analysis revealed nine putative ORFs, which were then individually subjected to coding potential analysis using CPC2 (Coding Potential Calculator 2). We conducted multiple BLAST alignments of *SPANXA2-OT1* and compared it with *HOTAIR*. **Supplementary Figure S5A** details the pipeline for the analysis of the lncRNA-miRNA-mRNA regulatory triad in CAD.

***Human primary macrophages***

Buffy coats derived from de-identified healthy donors were purchased from Research Blood Components, LLC (Boston, MA). The company has a New England Institutional Review Board-approved protocol for the Collection of White Blood for Research Purposes (NEIRB#04-144). Human peripheral blood mononuclear cells (PBMC), isolated by density gradient centrifugation from buffy coats, were cultured in RPMI-1640 (Thermo Fisher Scientific, Waltham, MA) containing 5% human serum and 1% penicillin/streptomycin (P/S; Thermo Fisher Scientific, Rockford, IL, USA) and were incubated in a humidified incubator at 37 °C with 5% CO2 for 10 days to differentiate into macrophages. In stimulation assays, confluent macrophages were rinsed extensively with PBS, starved for 24 hours and treated with inflammation stimulators: IL-1β (10 ng/ml, Sigma-Aldrich), INF-γ (10 ng/ml, Sigma-Aldrich), LPS (10 ng/ml, Sigma-Aldrich) and TNF-α (10 ng/ml, Sigma-Aldrich) for 6 hours.

***Cell lines***

We used several cell lines for this study which included THP-1 monocytes (human monocyte cell line derived from an acute monocytic leukemia patient, ATCC), THP-1-derived macrophages, Jurkat cells (immortalized line of human T lymphocytes, ATCC), and BLaER1 (human B-cell precursor leukemia cells, genetically modified to allow for transdifferentiation into models resembling human monocytes, Sigma-Aldrich) cells. Cells were maintained in RPMI 1640+10%FBS. For THP-1 derived macrophages, cell line was obtained from ATCC, and maintained at 2×10^5 cells/ml in RPMI 1640 medium supplemented with 10% FCS. THP-1 cells (2×10^5/ml) were differentiated with 200 nM phorbol 12-myristate 13-acetate (PMA, Sigma-Aldrich) for 3 days. THP-1 derived macrophages ( ATCC) was used as cell system to perform luciferase reporter assays.

***RNA interference and miRNA mimic***

RNA interference was performed as previously described (17). For knockdown of *SPANXA2-OT1*, we used antisense oligos (ASOs) designed and synthesized by AUM LifeTech (Philadelphia, PA, USA). ASO (5'-TATAACTTTGGGGAGGGCTGC-3') was added into each well at a final concentration of 0.5 mM. Scrambled control (cat# HMC0002) and *miR-338* miRNA (HMI0495) mimics were obtained from Sigma-Aldrich. For transcript-specific knockdown of *SPANXA2-OT1*, we designed ASOs targeting unique exon–exon junctions and intron-retained regions that are exclusive to *SPANXA2-OT1* and do not overlap with any coding exons of nearby genes. This design ensured high specificity to the lncRNA transcript. To confirm that our manipulations did not inadvertently affect nearby protein-coding genes, we performed qPCR assays on a panel of neighboring and overlapping genes in the *SPANX* gene cluster, including *SPANXA2* and *SPANXN1*, as well as control gene *GAPDH.*

***RNA extraction and quantitative real-time PCR***

Total RNA was isolated using Trizol (Invitrogen, USA) followed by DNase I treatment. cDNA was synthesized using EasyQuick RT MasterMix (CWbio, cat# CW2019M). Real-time qRT-PCR was performed using a QuantStudio 5 (ABI, CA, USA) according to the manufacturer's recommendations. The relative quantification of gene expression was performed using the comparative -ΔΔCT method.

***RNA in situ hybridization***

A customized probe for *SPANXA2-OT1* was specifically developed to detect ENSG00000277215 (Advanced Cell Diagnostics). Human primary macrophages were fixed in 4% paraformaldehyde and the in situ hybridization (ISH) protocol for cultured adherent cells was performed as described

by the manufacturer (RNAscope 2.5 HD Reagent Kit-Red; Advanced Cell Diagnostics, 322350). We further performed RNA-Protein Co-localization using RNAscope in THP-1–derived Macrophages. THP-1 cells were differentiated into macrophages using PMA and subjected to RNA–protein colocalization analysis using the RNAscope™ RNA-Protein Co-Detection Kit (Advanced Cell Diagnostics, RNA-Protein Co-detection Ancillary Kit Cat No. 323180). Cells were incubated for immunofluorescence staining with anti-AGO2 (clone AGO2 11A9, Cat# 14-6519-82, Thermo Fisher) and anti-DCP1a (clone JB51-34, Cat# MA5-34669, Thermo Fisher) followed by hybridization with a probe targeting the lncRNA *SPANXA2-OT1*. Nuclei were stained with DAPI.

***Vector construction and luciferase reporter assay***

Custom plasmid vector constructs for wild type and mutant *miR-338* binding site for *SPANXA-2-OT1* and *IL-8* were ordered from Genecopoeia (Rockville, USA). The sequence of vector constructs used in this study is shown in **Supplementary Figure S6**. These vector constructs were cloned in NEB 5-alpha competent *E. coli* and then the constructs were isolated from the bacteria using hispeed plasmid maxi kit (Qiagen, USA). The vector constructs were co-transfected with *miR-338* mimic or miRControl in THP-1 derived macrophages (n=5 replicates/group). Relative luciferase activity was normalized to Renilla luciferase activity 24 hours after transfection and was measured using a Luc-Pair Duo-Luciferase Assay Kit (Genecopoeia, Rockville, USA) according to the manufacturer’s protocol.

***RNA-binding protein immunoprecipitation (RIP) assay***

We used EZ-Magna RIP Kit (Millipore, USA) to perform RIP assays. After the lysis of cells in complete RIP lysis buffer, the cell extracts were incubated with RIP buffer containing magnetic beads conjugated to 5 µg anti-Ago2 antibody (Millipore, USA, cat# MABE56) per

immunoprecipitation. Samples were incubated with proteinase K with shaking to digest the proteins and the immunoprecipitated RNA was isolated. *SPANXA2-OT1* and *miR-338* levels in the precipitates were determined by qRT-PCR analysis.

***Unbiased global proteomics***

Four PBMC-derived macrophage conditions were analyzed: 1) Untreated control, 2) IL1β treated, 3) IL1β+negative control ASO and 4) IL1β+*SPANXA2-OT1* (n=5 PBMC donors). Human primary macrophage cell lysates was prepared using RIPA buffer, and proteolysis was performed using the standard protocol provided by the PreOmics iST Kit (PreOmics GmbH, Germany). Peptides were analyzed on the Orbitrap Fusion Lumos coupled to an Easy-nLC1000 HPLC pump (Thermo Fisher Scientific). The peptides were diluted 5-fold using sample loading buffer and 4 µl injections separated using a dual column set-up: an Acclaim™ PepMap™ 100 C18 HPLC Column, 75 µm X 70 mm (Thermo Fisher Scientific, Cat# 164946); and an EASY-Spray™ HPLC Column, 75 µm X 250 mm (Thermo Fisher Scientific, Cat# ES902). The column was heated at a constant temperature of 45 ˚C. The gradient flow rate was 300 nl/min from 5 to 21% solvent B (0.1% formic acid in acetonitrile) for 75 minutes, 21 to 30% solvent B for 15 minutes, and another 10 minutes of a 95%-5% solvent B sawtooth wash . Solvent A was 0.1% formic acid in mass spectrometry-grade water. The mass spectrometer was set to 120,000 resolution, and the top N precursor ions in a 3 second cycle time (within a scan range of m/z 400-1500; isolation window, 1.6 m/z) were subjected to collision-induced dissociation (CID, collision energy 30%) for peptide sequencing.

The acquired peptide spectra corresponding to 20 raw spectral files (four conditions X 5 donors) were searched with Proteome Discoverer package (PD, Version 2.5) using the SEQUEST-HT search algorithm against the Human UniProt database (downloaded January 18, 2022; n=100,730 entries). The digestion enzyme was set to trypsin and up to two missed cleavages were allowed. The precursor tolerance was set to 10 ppm and the fragment tolerance window to

0.6 Da. Methionine oxidation and n-terminal acetylation were set as dynamic modifications, and cysteine carbamidomethylation as a static modification. The peptide false discovery rate (FDR) was calculated by the PD Percolator algorithm and peptides were filtered based on an FDR threshold of 1.0%. Peptides that were only assigned to one given protein group and not detected in any other protein group were considered unique and used for further analyses. A minimum of 2 unique peptides for each protein were required for the protein to be included in the analyses. The Feature Mapper was enabled in PD to quantify peptide precursors detected in the MS1 but may not have been sequenced in all 20 samples. Chromatographic alignment was performed with a maximum retention time shift of 10 minutes, mass tolerance of 10 ppm and signal-to-noise minimum of 5. Precursor peptide abundances were based on their chromatographic intensities and total peptide amount was used for PD normalization. Proteomics were quantified in Proteome Discoverer v2.5, normalized by total peptide amount and median abundance, and differential enrichment was assessed in Qlucore Omics Explorer v3.7 through ANOVA (mixed-effects as appropriate) at a Benjamini-Hochberg false discovery rate (FDR; q/adjusted P value)≤0.05. XINA (18) was used for clustering the protein abundance clusters using sum-normalized quantified data. **Figure 4A** represents the workflow of proteomics experiments.

***Unbiased global transcriptomics***

Four PBMC-derived macrophage conditions were analyzed: 1) IL1β+negative control ASO and 2) IL1β+*SPANXA2-OT1* ASO (n=4 PBMC donors).RNA was isolated from macrophage cell lysate using RNAeasy mini kit (Qiagen). RNA-sequencing mRNAseq (polyA enriched) library prep, single-end 75bp sequencing on NextSeq of 12-16 pooled barcoded samples, and VIPER analysis(19) was performed at the Molecular Biology Core Facilities at Dana-Farber Cancer Institute. The data were then analyzed using DESeq2. A minimum of 40 million raw reads was generated from each library. Sequenced data was processed to generate FASTQ files with

sequence bases >Q30 value and mapped to the reference human genome assembly GRCH38/hg38. **Figure 4A** represents the workflow of transcriptomics experiments.

***Western blot analysis***

Proteins were extracted from cell lysates using RIPA buffer containing protease inhibitors. 20 μg protein was separated by 6% sodium dodecyl sulfate–polyacrylamide gel electrophoresis (SDS-PAGE) and transferred to polyvinylidene difluoride (PVDF) membrane. The membranes were incubated overnight with antibodies to IL-8 (1:1000, Abcam, USA, Cat# ab110727) followed by incubation with the corresponding secondary antibody. The bands were visualized using the enhanced chemiluminescence method. Protein expression was quantified and normalized to β-actin (1:2000, Cell signaling, USA, Cat# 4967). Schematic workflow for gene expression studies in *SPANXA2-OT1* silenced macrophages is presented in **Figure 5A.**

***CRISPR/Cas9 mediated deletion of the SPANXA2-OT1 functional domain (exon 3) in human primary macrophages***

Human primary macrophages were plated on 10 cm dishes and cultured for 7 days. Cells were detached using Accutase and scraping. After viability assay cells were treated with 100 pmol Truecut Cas9 protein (Thermo Fisher) and 250 pmol of multi guide RNA against GAS6 (Synthego). Cells were electroporated using P3 primary cell 4D Nucleofector Kit (Lonza) and protocol ID– CM 137.  Cells were seeded into 24-well plates at 3X10^5 cells per well and allowed to recover for 5 days prior to experiment. Method details are depicted in the schematic of **Figure 6A. Figure 6B** depicts the target sequence and guide RNA equence for deletion of exon 3 on *SPANXA2-OT1* gene**. Supplementary Figures S7** represent the knockout score of CRISPR/Cas9 deletion on *SPANXA2-OT1*.

***48-Plex cytokine/chemokine panel***

Frozen supernatant media samples from the culture of CRISPR/Cas9 deleted *SPANXA2-OT1* (exon 3) macrophages were shipped to Eve Technologies (Calgary, Alberta, Canada) on dry ice and were profiled using the 48-Plex Discovery Assay (Human Cytokine/Chemokine Panel A 48-Plex Discovery Assay-HD48A) according to the manufacturer protocol. For the Discovery Assay, a protein standard consisting of purified cytokines at known concentrations was included in each batch run; absolute concentrations were calculated from the standard curve and reported as pg/mL. All values reported as out of range below the 4 or 5 parameter logistic standard curve (OOR<) were replaced with “0,” per the manufacturer protocol. Additionally, all instances of out of range above the 4 or 5 parameter logistic standard curve (OOR>) were replaced with the highest observed concentration from the standards, and cytokine data reported as out of range values but not specified as OOR< or OOR> were imputed using a point-to-point semi-log regression to the data, per manufacturer protocol.

***IL-8 Protein Quantification by ELISA***

IL-8 secretion from human primary macrophages was measured using a Quantikine® ELISA Kit (R&D Systems, Cat# D8000C), according to the manufacturer’s instructions. Briefly, media supernatants were collected from macrophages derived from peripheral blood mononuclear cells (PBMCs) of five individual donors following *SPANXA2-OT1*-Exon 3 deletion. Samples were run in duplicate, and absorbance was measured at 450 nm with wavelength correction at 540 nm. Standard curves were used to interpolate IL-8 concentrations. Data are expressed as mean ± SEM. Statistical significance was determined using one-way ANOVA followed by post hoc multiple comparisons.

***Chemotaxis assay and image quantification***

For real-time chemotaxis assays, we used the IncuCyte S3 System. For this assay, we used the supernatant from following group of cultured human primary macrophages: (a) complete media (RPMI1640+5% human serum), (b) complete media + IL-1β (10 ng/ml), (c) complete media + IL-1β (10 ng/ml) + Scrambled gRNA (Scr) and (d) complete media + IL-1β (10ng/ml) + SPANXA2-OT1-exon3 gRNA (E3). Incucyte clearview 96-Well chemotaxis plates (Essen Biosciences) were coated with matrigel matrix (Corning). In the bottom chamber, we added 200μL of the above mentioned attractants. In the top chamber, we added 3.3x10^4 cells resuspended in RPMI 1640 (phenol-red free) plus 0.5% human serum. We performed chemotaxis assay on following cell types: (a) human PBMCs, (b) primary human macrophages, (c) THP-1 monocytes, (d) THP-1 derived macrophages, (e) Jurkat cells (T-cell) and (f) BLaER1 cells (B-cells). Plates were placed into IncuCyte S3 and images were acquired every 30 minutes, for 12 hours. Chemotaxis quantification was performed by Incucyte chemotaxis analysis software module. Schematics for chemotaxis assay followed by CRISPR/Cas9 deletion of SPANXA2-OT1-Exon3 are presented in **Figure 7A** and **Supplementary Figure S7A**.

**Macrophage Chemotaxis Assay with IL-8 Neutralization**

Human peripheral blood-derived macrophages from five independent donors were subjected to live-cell chemotaxis assays for 12 hours in the presence of conditioned media obtained from control or *SPANXA2-OT1* exon-3–deleted cells. To assess the role of IL-8, conditioned media were pretreated with a neutralizing antibody against human IL-8/CXCL8 (Cat# MAB208-100, R&D Systems) at concentrations of 0.1, 0.5, and 1 μg/mL or with an matching concentration of isotype control antibody. Live-cell imaging was performed at 37°C using a phase-contrast microscope equipped with a time-lapse module. Migrated macrophages were tracked using time-lapse images, and quantification Chemotaxis quantification was performed by Incucyte chemotaxis analysis software module.

***Harvesting of Ldlr-/- mouse tissue***

Animal experiments were approved by the Brigham and Women's Hospital's Animal Welfare Assurance (protocol 2016N000219). 10-week-old *Ldlr-/-* mice were sacrificed and aortas were carefully dissected from surrounding tissues, harvested, and cleaned using a cold saline buffer. Subsequently, aortas were immediately snap frozen to isolate the RNA for gene expression experiments.

A

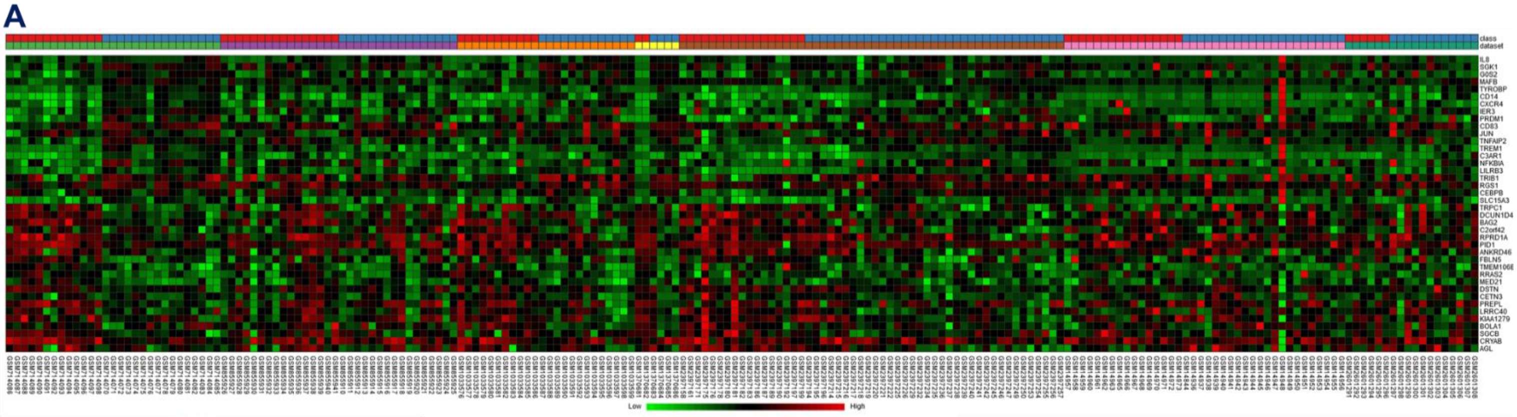


B

C

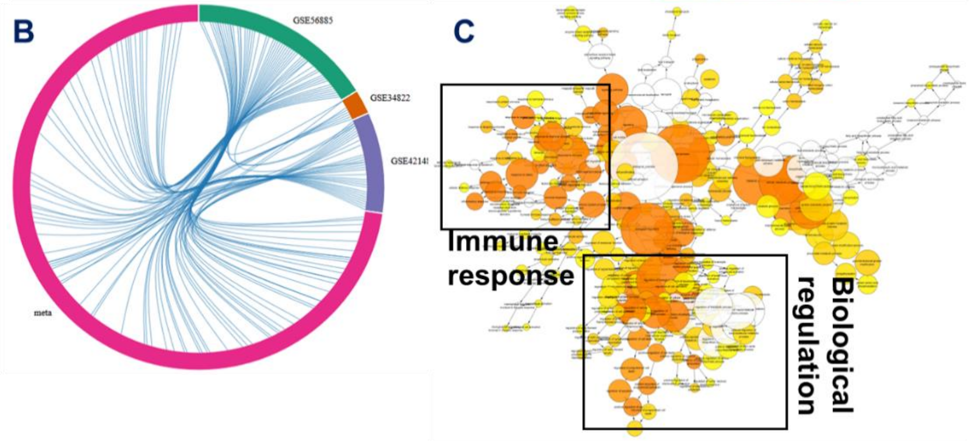


D

| Enrichment Term | Pathway/ Term ID | Overlap | AdjP-value |
|---|---|---|---|
| immune response | 6955 | 194/619 | 1.36E-25 |
| immune system process | 2376 | 257/948 | 3.49E-24 |
| response to stress | 9650 | 399/1775 | 2.69E-21 |
| defense response | 9652 | 177/621 | 1.75E-18 |
| biological regulation | 65007 | 1160/6913 | 7.81E-16 |

**Figure S1- Differentially expressed mRNA analysis in CAD.** (A) Heat-map representation of expression profiles for top 40 differentially expressed mRNA obtained from meta-analysis. (B) All datasets were included in the meta-analysis and contributed to the overall differential expression statistics, but for the purpose of the chord plot, only the three datasets (based on DEG overlap or contribution) are shown. (C) Enrichment network of shared DEGs based on biological processes. Significantly overrepresented biological processes based on GO terms were visualized in Cytoscape. The size of a node is proportional to the number of targets in the GO category. The color represents enrichment significance— the deeper the color on a color scale, the higher the enrichment significance. P-values were adjusted using a Benjamini and Hochberg False Discovery Rate (FDR) correction. The square box represents the immune related pathway cluster. (D) Table showing top five GO terms associated with the DE mRNAs. Overlap: indicates the number of hits from the meta-analysis compared to each curated gene set library; GO: gene ontology biological process.

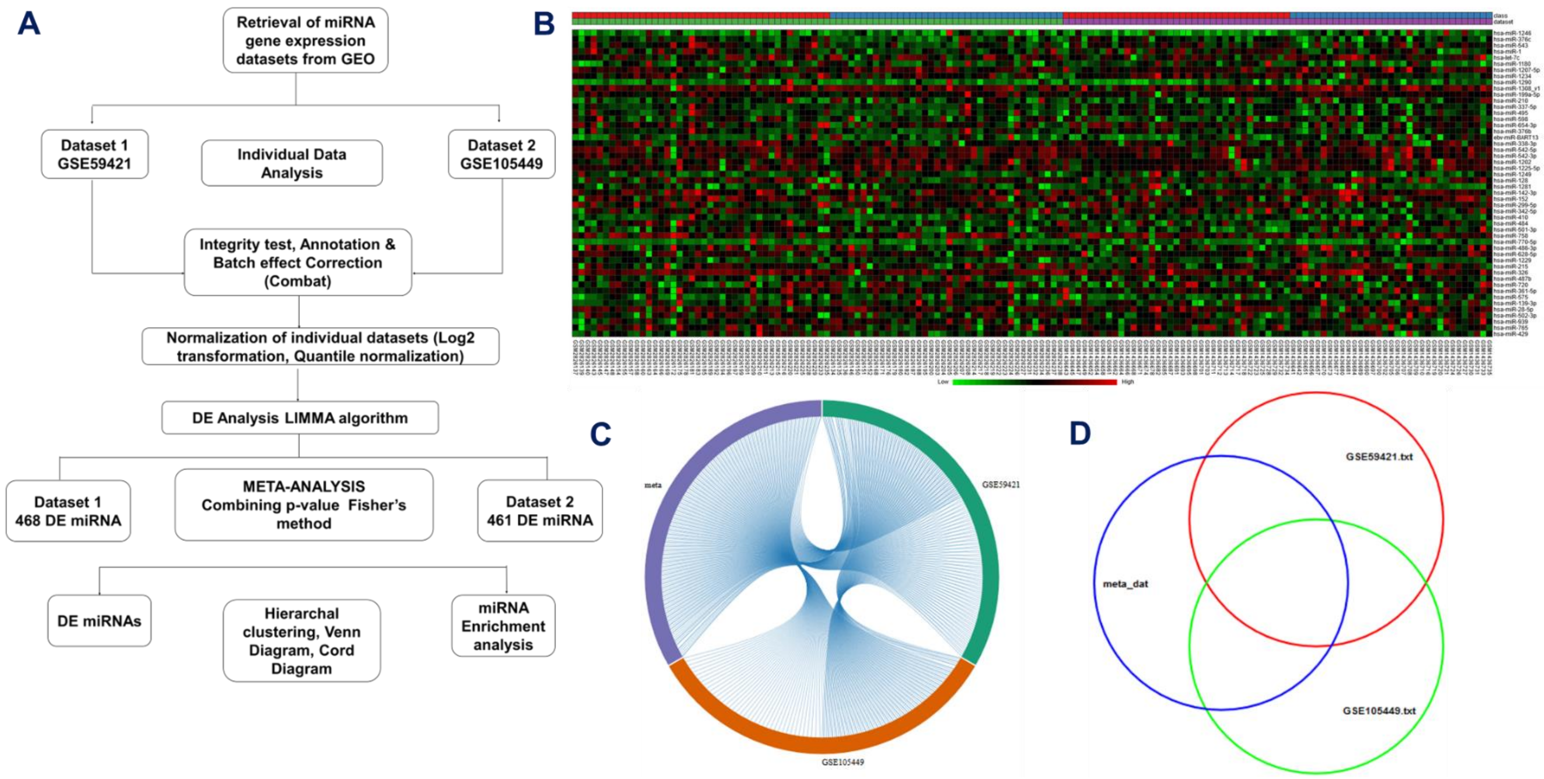


**Figure S2- Differentially expressed microRNA (miRNA) analysis in CAD**. (A) Depiction of the flow chart of the process involved in integrated meta-analysis of the selected miRNA microarray datasets from the CAD patient samples from human studies. (B) Heat-map representation of expression profiles for the differentially expressed miRNA obtained from meta-analysis. (C) Chord diagram showing the differentially expressed miRNAs connection between the individual datasets and the meta-data. (D) Venn diagram of differentially expressed miRNAs identified from the meta-analysis (Meta-DE) and those from each individual microarray analysis (Individual-DE).

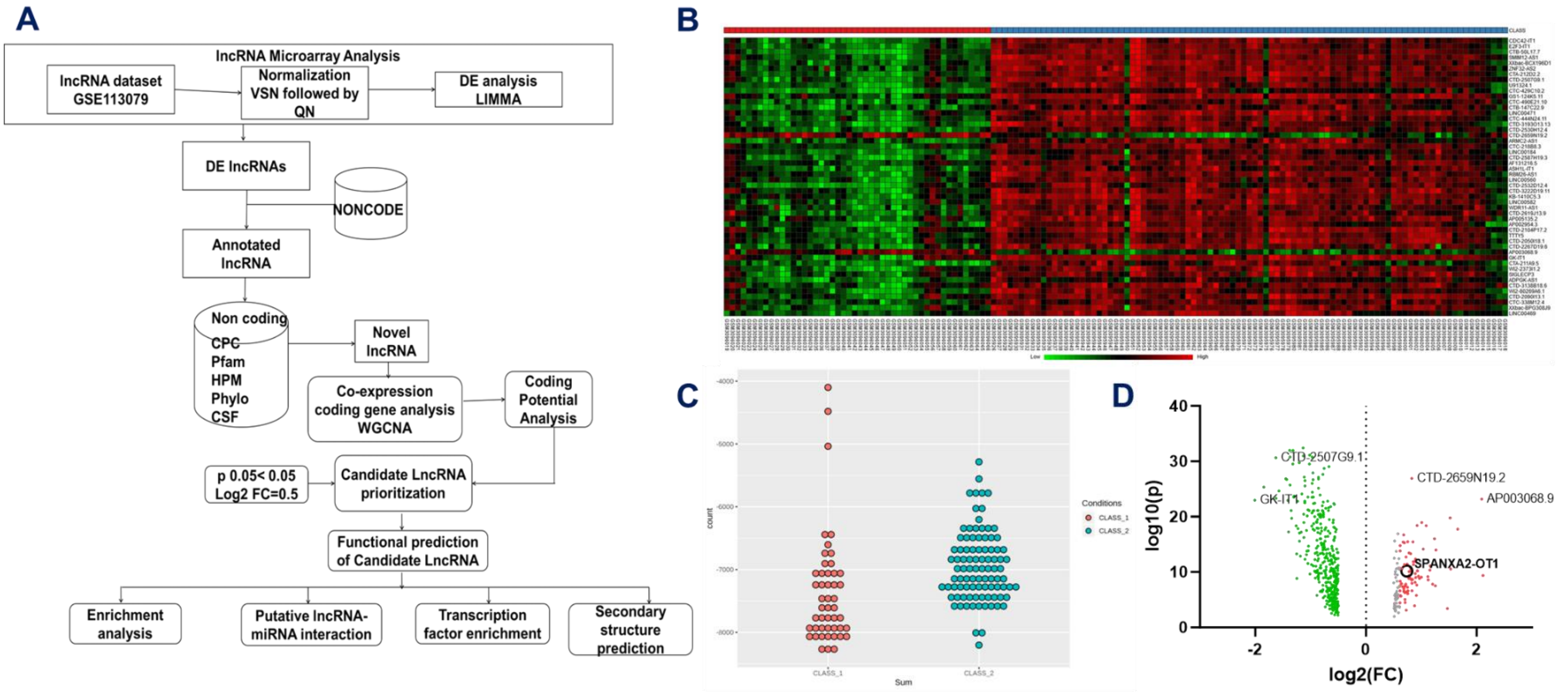


**Figure S3- Differentially expressed long noncoding RNA (lncRNA) analysis in CAD**. (A) Pipeline for the analysis of GSE113079 for identification of long non-coding landscape in CAD. (B) Heat-map representation of expression profiles for the differentially expressed lncRNAs obtained from analysis of GSE113079. Colored bar on top represents; red (control) and blue (CAD). (C) Dataset GSE113079 library size. Class_1 are normal samples and Class_2 are CAD patient samples. (D) Volcano plot: Red dots denote the statistically significant overexpressed (adjusted p-value and log-fold change); green dots denote the statistically significant underexpressed (adjusted p-value and log-fold change), while the grey dots denote statistically non-significant lncRNAs. SPANXA2-OT1 is highlighted as one of the overexpressed lncRNAs in our analysis.

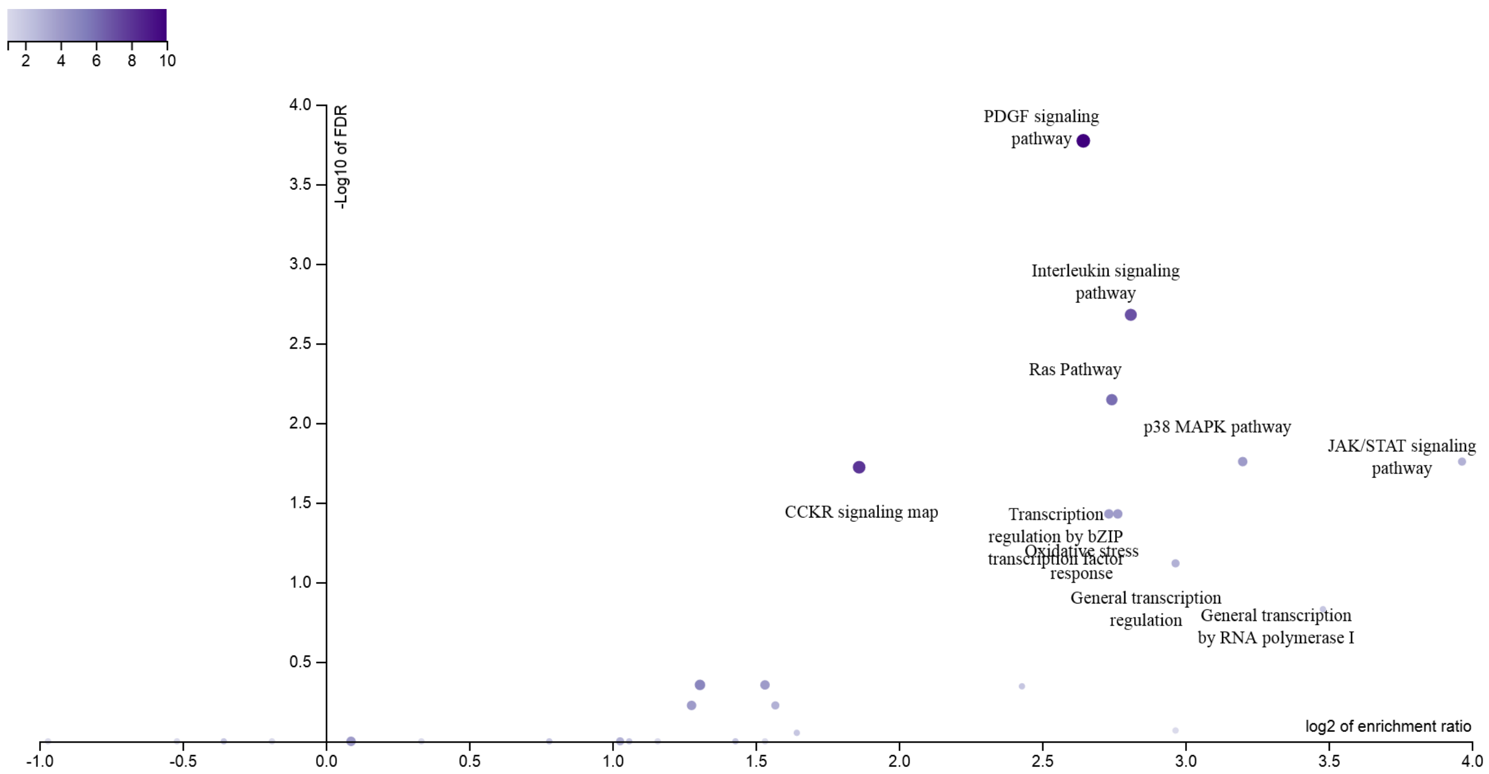


**Figure S4- Enrichment analysis of the candidate lncRNA associated transcription factors.** Darker blue color represents increasingly enriched pathways using panther database. Several pathways related to chemotaxis signaling were enriched in our analysis. Following list of transcription factor was used for enrichment analysis: AP-2alpha, AP-2gamma, ARID3A, ATF1, ATF2, ATF3, BAF155, BATF, BCL11A, BCL3, BCLAF1, BHLHE40, BRCA1, CBX3, CCNT2, CEBPB, c-Fos, CHD1, CHD2, c-Jun, c-Myc, COREST, CTCFL, E2F1, E2F4, E2F6, EBF1, Egr-1, ETS1, FOS, FOSL1, FOSL2, FOXA1, FOXA2, FOXM1, FOXP2, GABP, GATA2, GATA-2, GTF2B, GTF2F1, HA-E2F1, HDAC1, HDAC2, HMGN3, HNF4G, IRF4, JunB, JunD, MafK, Max, MAZ, MEF2A, MTA3, Mxi1, NFATC1, NFIC, NF-YA, NF-YB, NR2F2, NRSF, p300, PAX5, PHF8, PLU1, PML, Pol2, Pol2-4H8, Pol2(phosphoS2), POU2F2, PU.1, RBBP5, RFX5, RUNX3, SAP30, SIN3A, Sin3Ak-20, SMC3, SP1, SRF, STAT3, STAT5A, TAF1, TAF7, TAL1, TBLR1, TBP, TCF7L2, TCF7L2_C9B9, TEAD4, TR4, UBF, UBTF, USF1, USF-1, USF2, WHIP, YY1, ZBTB7A, ZEB1, Znf143, ZNF263, Brg1, CtBP2, EZH2, Ini1, SUZ12, TCF12, Bach1, CEBPD, CTCF, ELF1, ELK1, GATA-1, HDAC6, JARID1A, MEF2C, NELFe, Pbx3, Rad21, RXRA, Sin3Ak, SIX5, SP2, TCF3, THAP1, TRIM28, ZBTB33, ZKSCAN1, ELK4, KAP1, MYBL2, Nrf1, RPC155, SP4, TFIIIC-110, HNF4A, GATA3, MafF, MBD4, SETDB1, ZNF217, BAF170, IRF3, PRDM1, STAT1, ZNF274, ZZZ3.

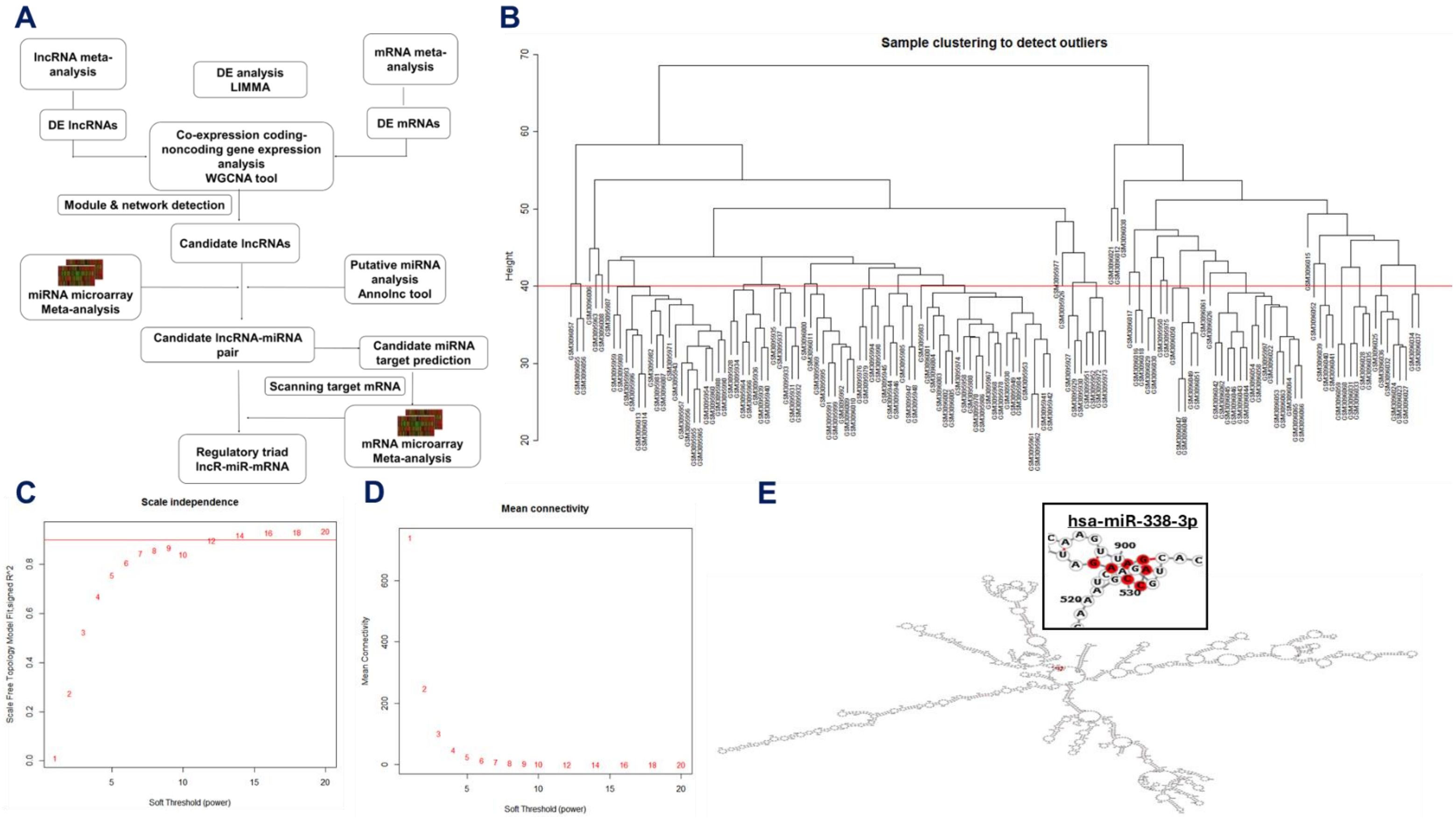


**Figure S5- Weighted gene correlation network analysis (WGCNA) for coding-noncoding module detection**. (A) Pipeline for the analysis of lncRNA-miRNA-mRNA regulatory triad in CAD. (B) Clustering dendrogram of samples based on their Euclidean distance using WGCNA algorithm. (C) The scale independence of WGCNA analysis. (D) The mean connectivity of WGCNA analysis. (E) The secondary structures of candidate lncRNAs-SPANXA2-OT1 were computed using RNAfold (minimum free energy= -247.1Kcal/mol). Complementary seed matching sites for predicted miR-338 is marked in red and enlarged.

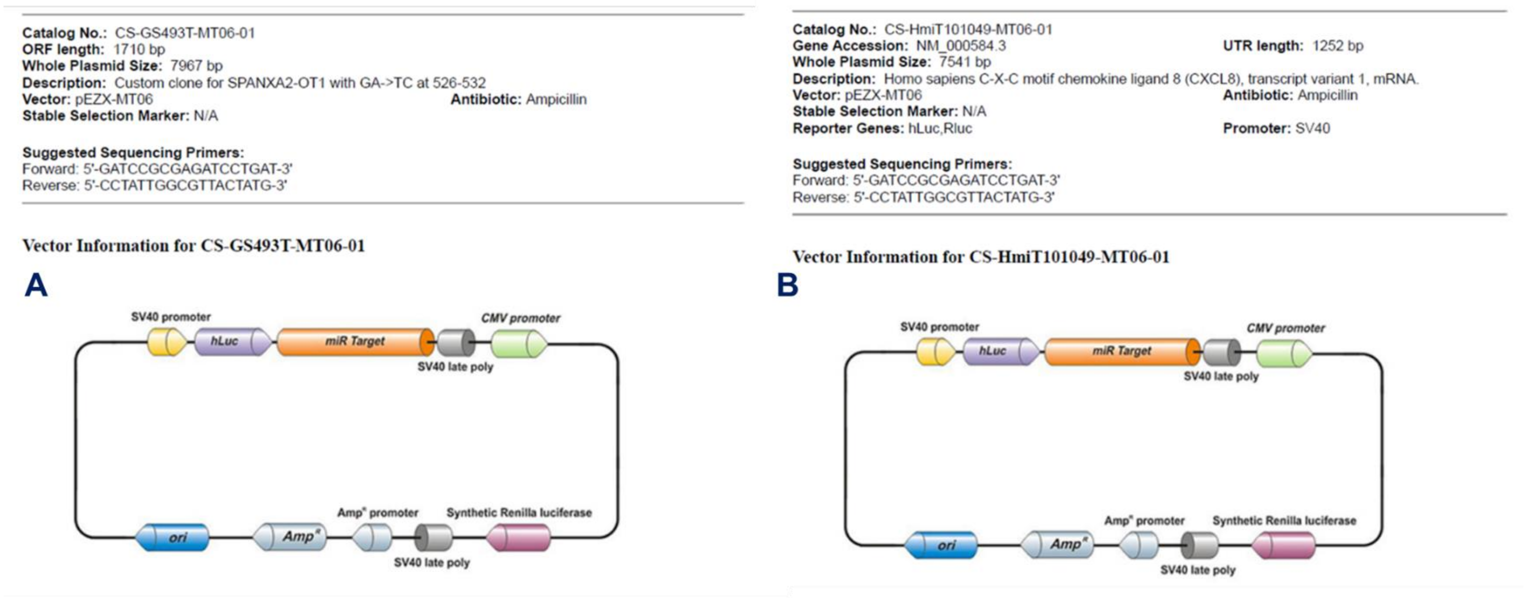


**Figure S6- Vector construct for luciferase assay**. (A) Mutant construct for SPANXA2-OT1 and (B) IL-8.

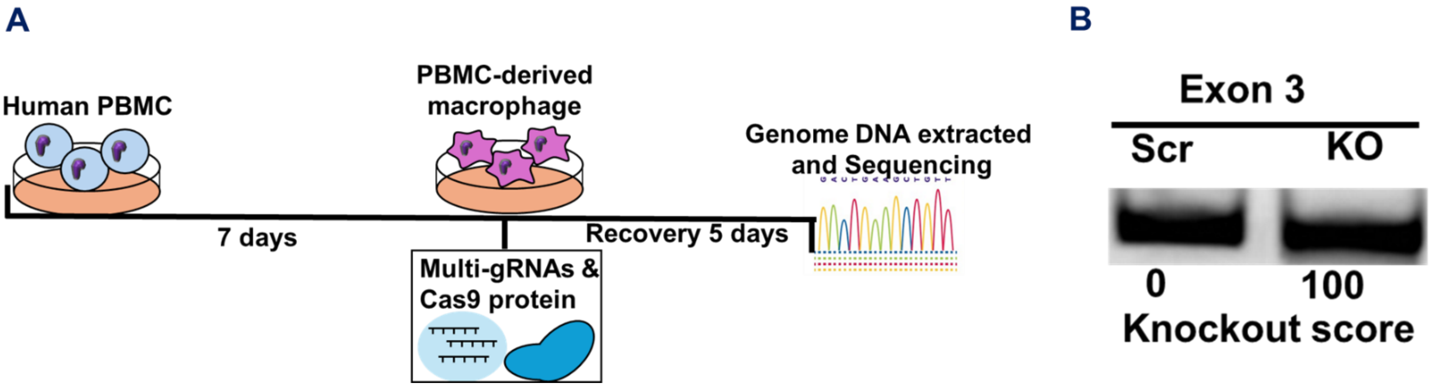


**Figure S7- CRISPR/Cas9 editing of SPANXA2-OT1 functional domain-** (A) Schematic depicting the CRISPR/Cas9 strategy for creating SPANXA2-OT1 exon 3 knockout human primary macrophage. (B) Knockout score of SPANXA2-OT1 (Exon 3); slight downward shift of band in the knockout group (KO) compared to scramble gRNA (Scr) highlights the exon 3 deletion.

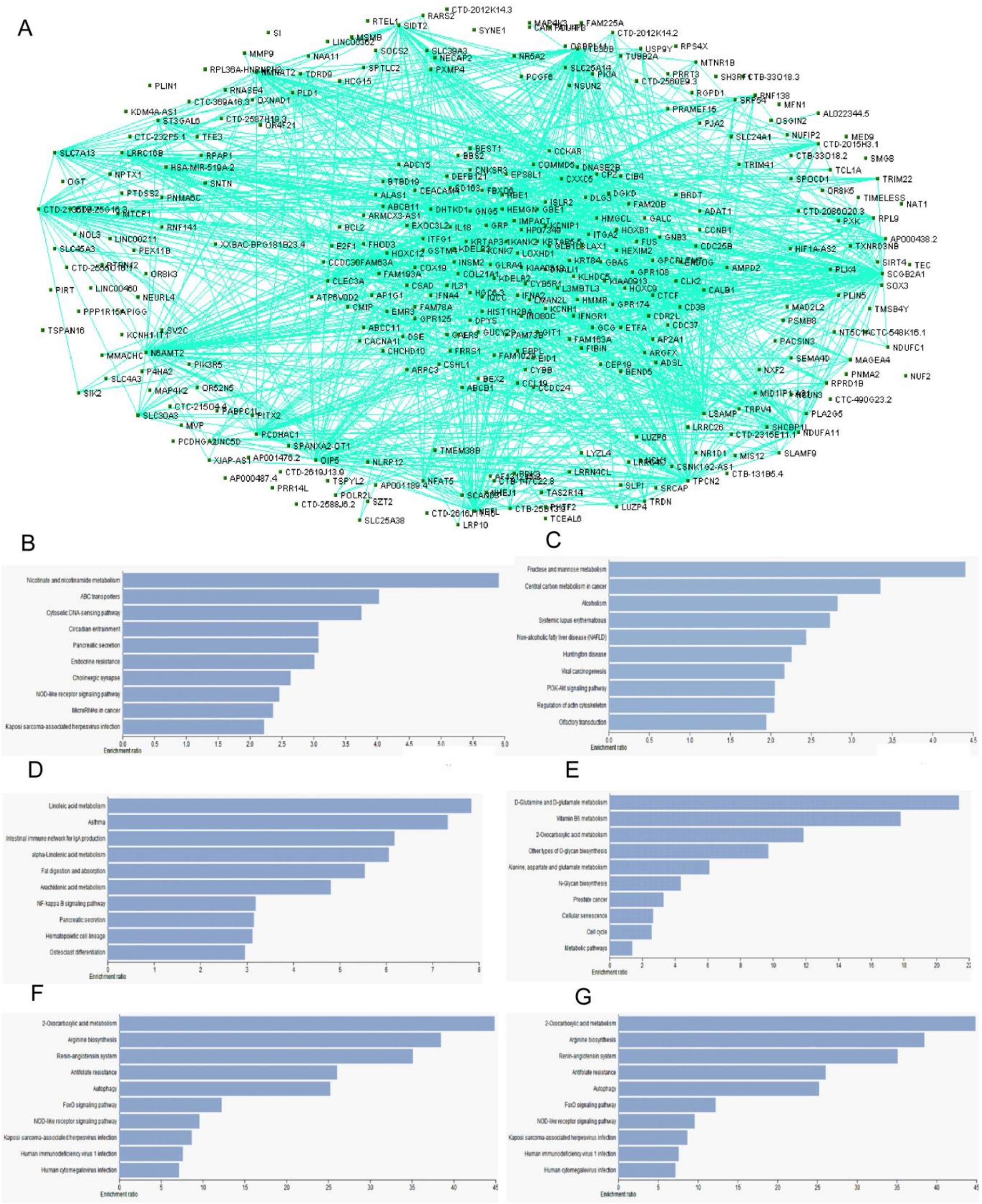


**Figure S8- Result of CAD related lncRNA-mRNA co-expression networks and their enrichment analysis.** (A) Construction of module 1 (Blue) lncRNA-mRNA co-expression networks based on WCGNA analysis. (B–G) Function annotation of CAD related lncRNAs. KEGG pathway analysis shows lncRNAs associated mRNA in module 1-Blue (B), module 2-Brown (C), module 3- Yellow (D), module 4- Red (E), module 5- Green (F) and module 6- Cyan (G) regulate multiple biological processes.

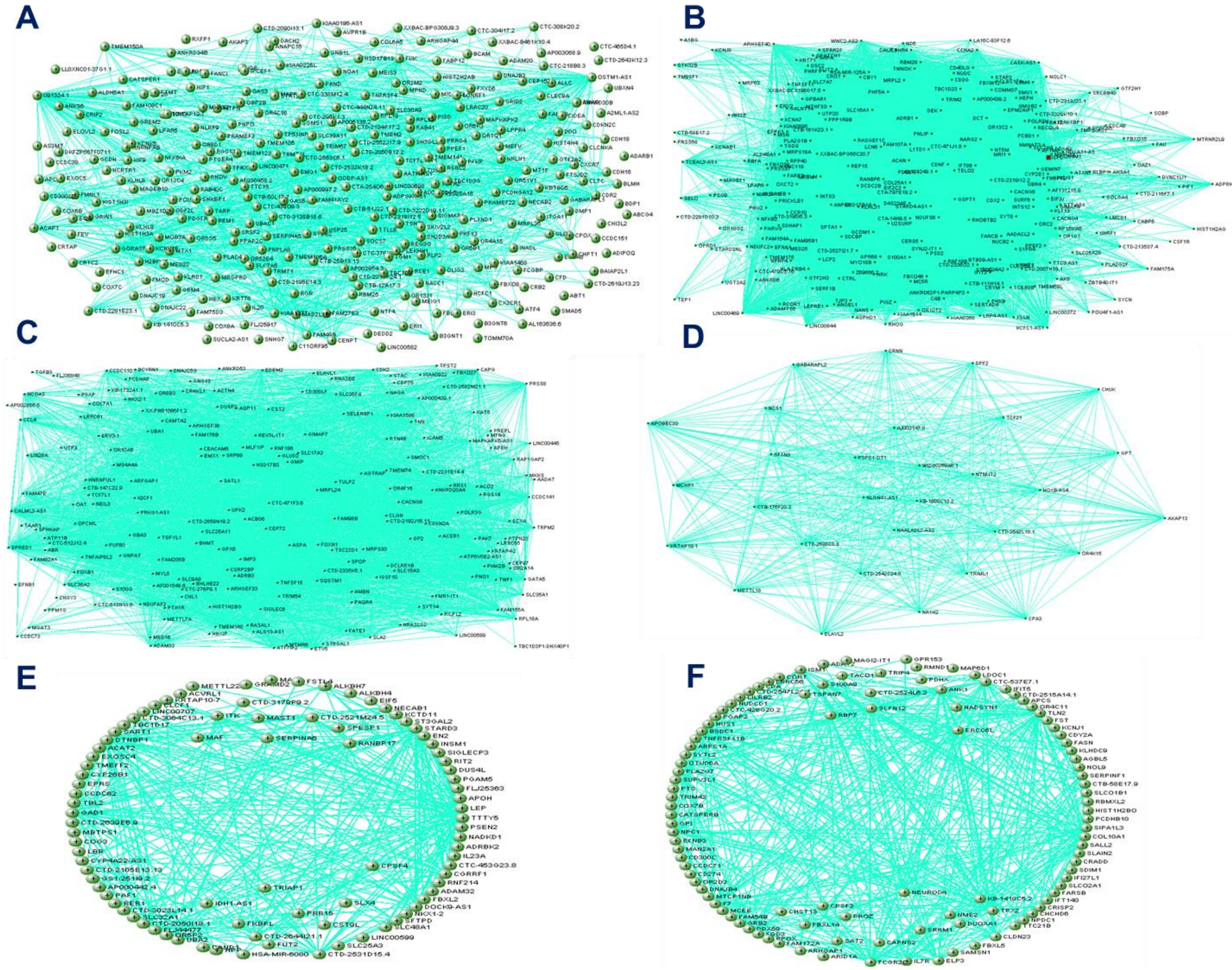


**Figure S9- Coding-noncoding network modules from WGCNA analysis.** (A) Brown; (B) Yellow; (C) Red; (D) Green; (E) Cyan and (F) Purple.

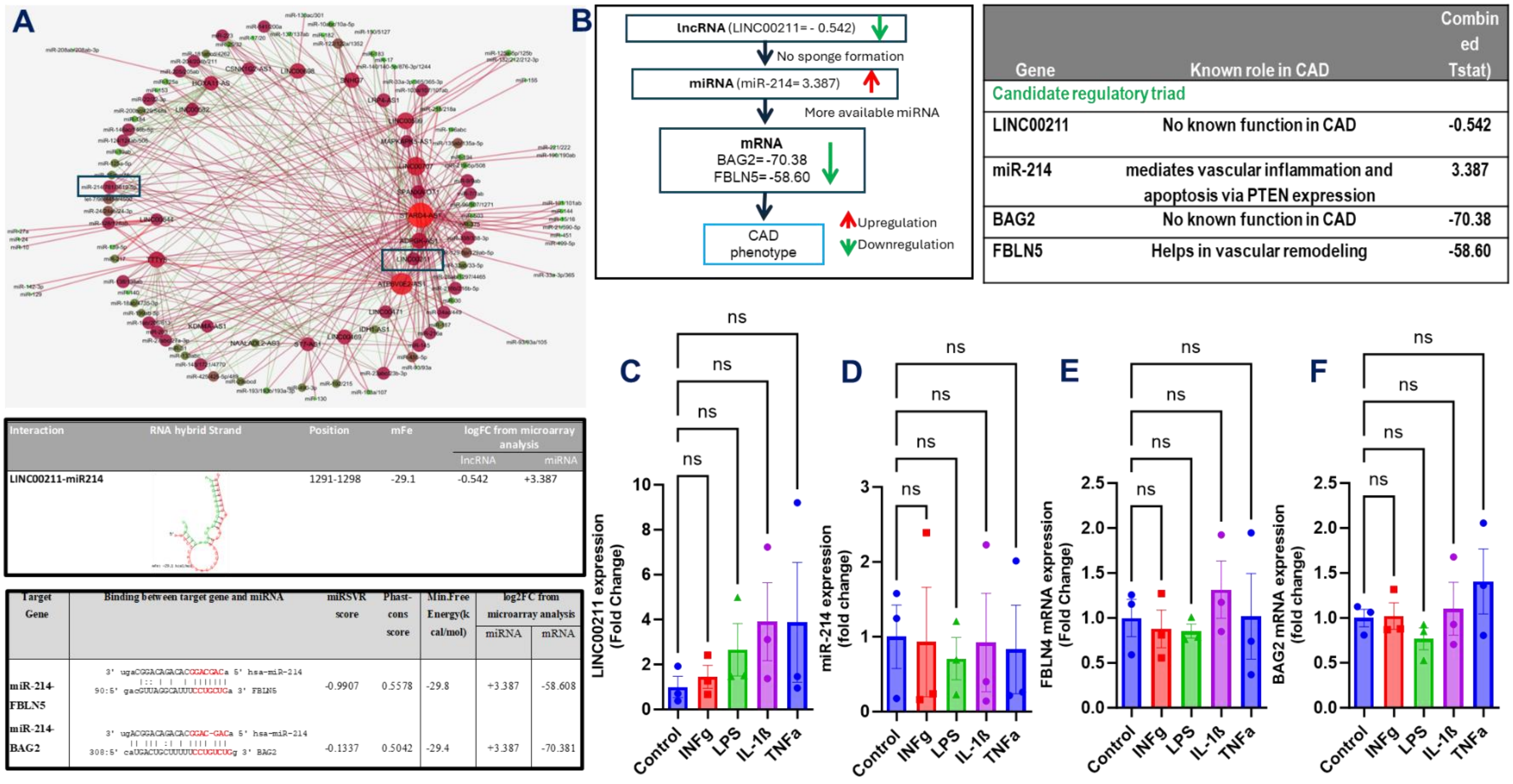


**Figure S10- Other triad validation data**. WGCNA and integrated analysis identified LINC00211-miR214-BAG2/FBLN5 triad as another potential regulatory triad in CAD. (A) Interaction network between lncRNAs and miRNAs involved in CAD which was obtained from annolnc analysis was constructed and analyzed using Cytoscape. The source nodes here are the lncRNAs and target nodes are miRNAs, while the edges represent the interaction between them. The node size is based on the degree of network with bigger node having greater degree. RNAfold analysis of miRNA-target genes interaction. Table depicting miR binding site on BAG2 and FBLN5, miRVR score was calculated by miRanda target prediction tool, binding energy and secondary binding structure were calculated using RNA HYBRID tool. (B) Schematic and table depicting the expression and function of triad component from our meta-analysis. (C-F) RNA expression of the triad components did not change in human primary macrophages after inflammatory stimuli (INFγ, IL1β, TNFα, LPS- all in the concentration of 10 ng/ml) for 6 hours. N=3/group. Statistical analysis was performed using one-way ANOVA with Tukey's multiple comparisons test. p-Values are denoted as: ***p <0.0001, **p <0.001, and *p <0.05.

**A**

| Sample | M_GAPDH (cT) | H_SPANXA (cT) |
|---|---|---|
| Control Aorta 1 | 19.97258614 | Undetermined |
| Control Aorta 2 | 21.12305672 | Undetermined |
| Control Aorta 3 | 22.23353732 | Undetermined |
| Control Aorta 4 | 20.6384025 | Undetermined |
| Control Aorta 5 | 20.90754684 | Undetermined |
| Control Aorta 6 | 19.91313932 | Undetermined |

**B**

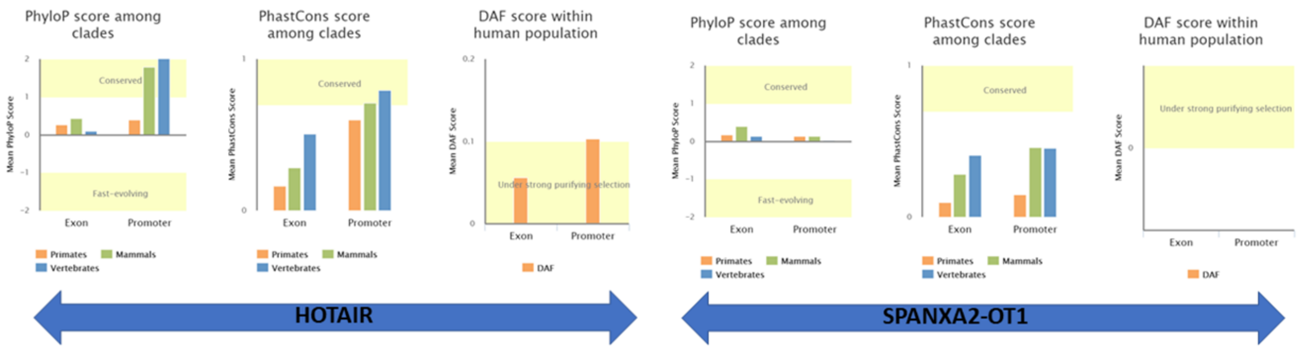


**Figure S11- SPANXA2-OT1 is not conserved across species.** (A) SPANXA2-OT1 is not expressed in mice aorta- qPCR using human SPANXA2-OT1 primers. We used RNA extracted from aorta of n= 6 mice. The table represents expression cT value of mice GAPDH and human SPANXA2-OT1 primers. (B) PhyloP score for conservation analysis of the SPANXA2-OT1 in comparison to conserved lncRNA-HOTAIR. PhyloP is a measure of conservation for every base. The chart here shows the mean phyloP scores of exons and promoter regions (upstream 1kb) in mammals. Positive phyloP means conservation and negative means fast-evolving. The results show SPANXA2-OT1 is poorly conserved across the species as compared to HOTAIR.

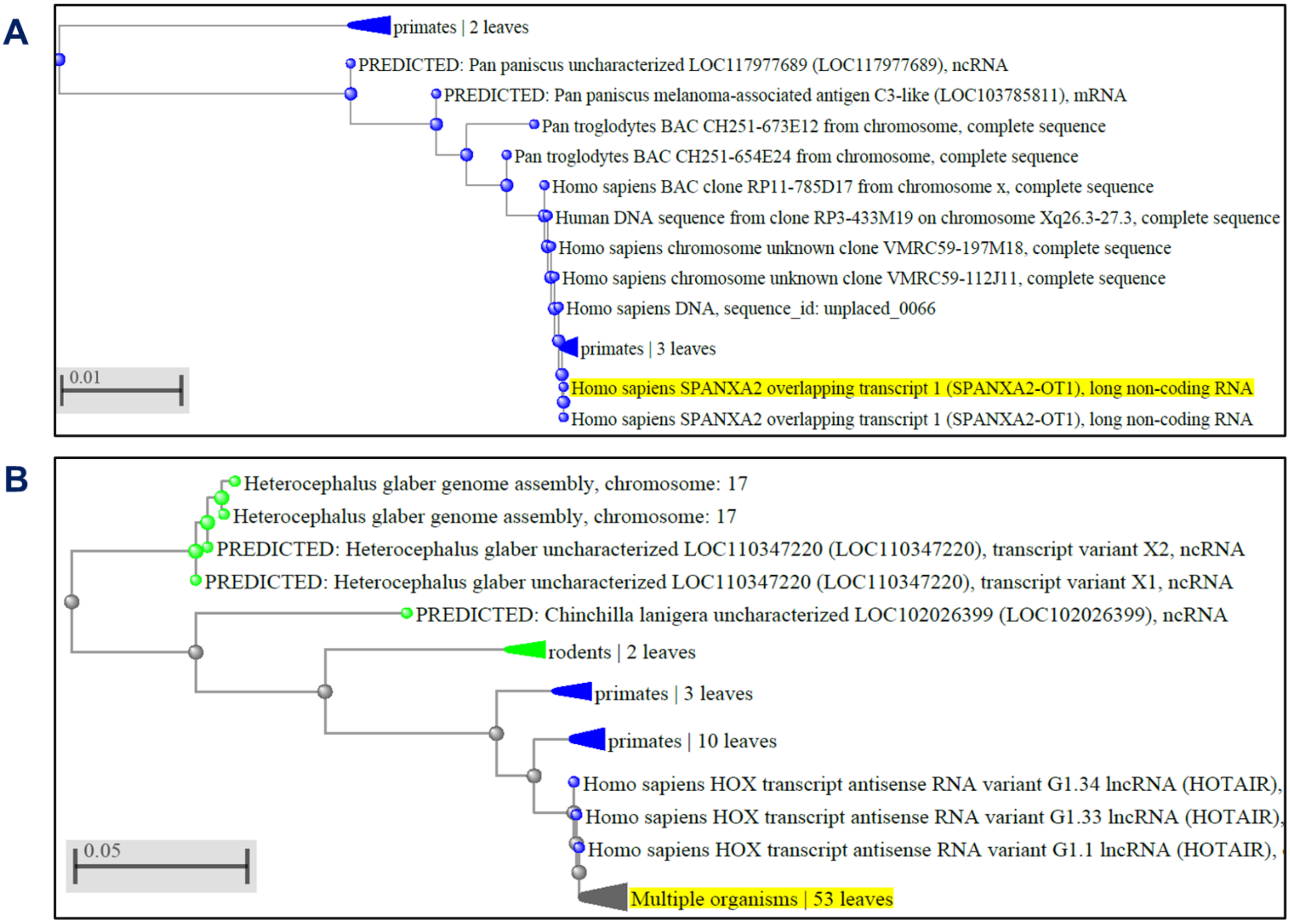


**Figure S12- Multiple BLAST alignment for conservation analysis.** (A) Demonstrates that SPANXA2-OT1 is only conserved in primates. (B) lncRNA-HOTAIR, a well-studied lncRNA is highly conserved across species.

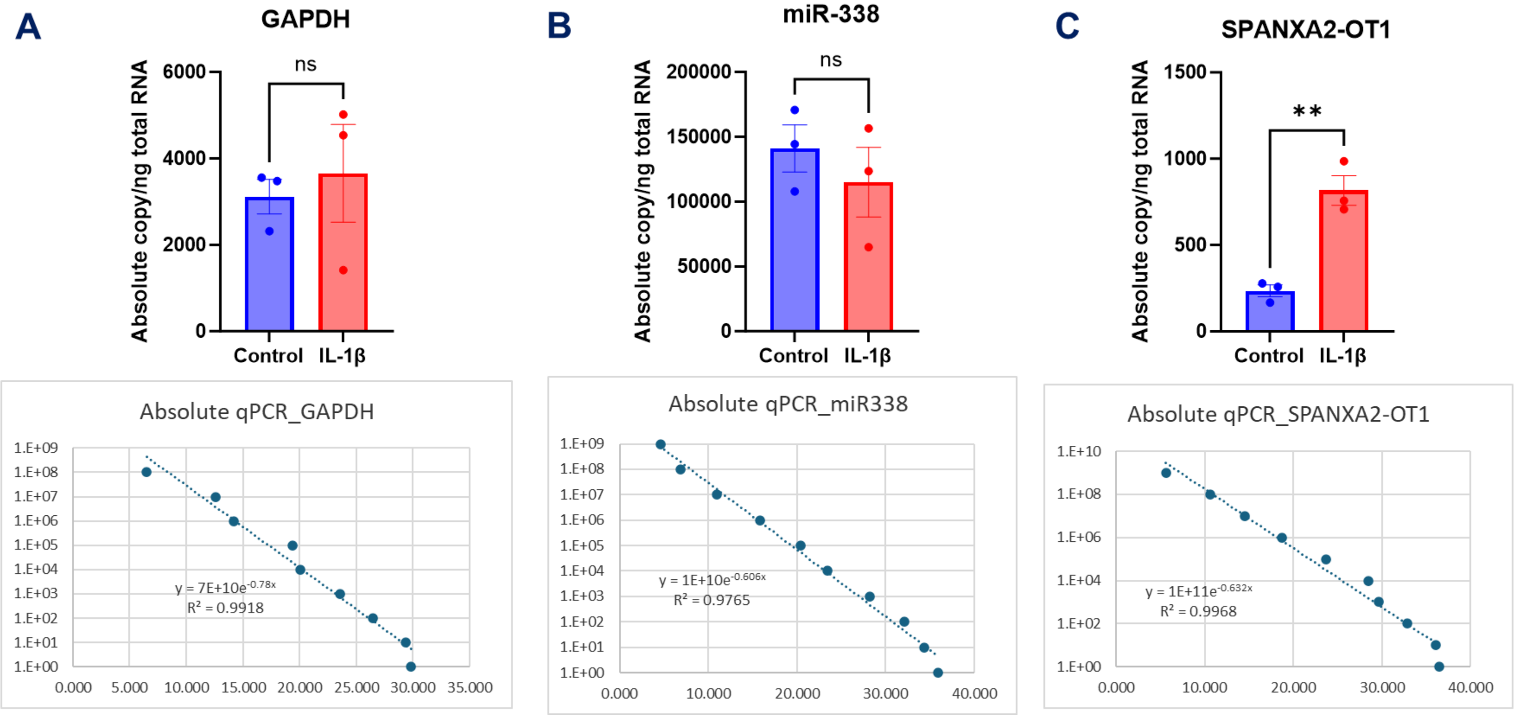


**Figure S13- Absolute copy number analysis-** Real-time PCR analysis for the absolute quantification (copy number, copies/ng total RNA) of GAPDH, miR-338 and SPANXA2-OT1 gene in human primary macrophages (n=3 donors). Serial diluted cDNAs of each standard sample and unknown samples were amplified and detected by SYBR green and primers of gene. The quantity (copy number, copies/ng) of each gene of each sample was calculated according to the corresponding standard curve, which was generated by plotting the Ct value against the logarithm of the quantities of the standard samples. We also compared the changes in absolute copy numbers of gene of interest and found that SPANXA2-OT1 copy number significantly increased after IL1β treatment. Statistical analysis was performed using unpaired student's T-test. p-Values are denoted as: ***p <0.0001, **p <0.001, and *p <0.05.

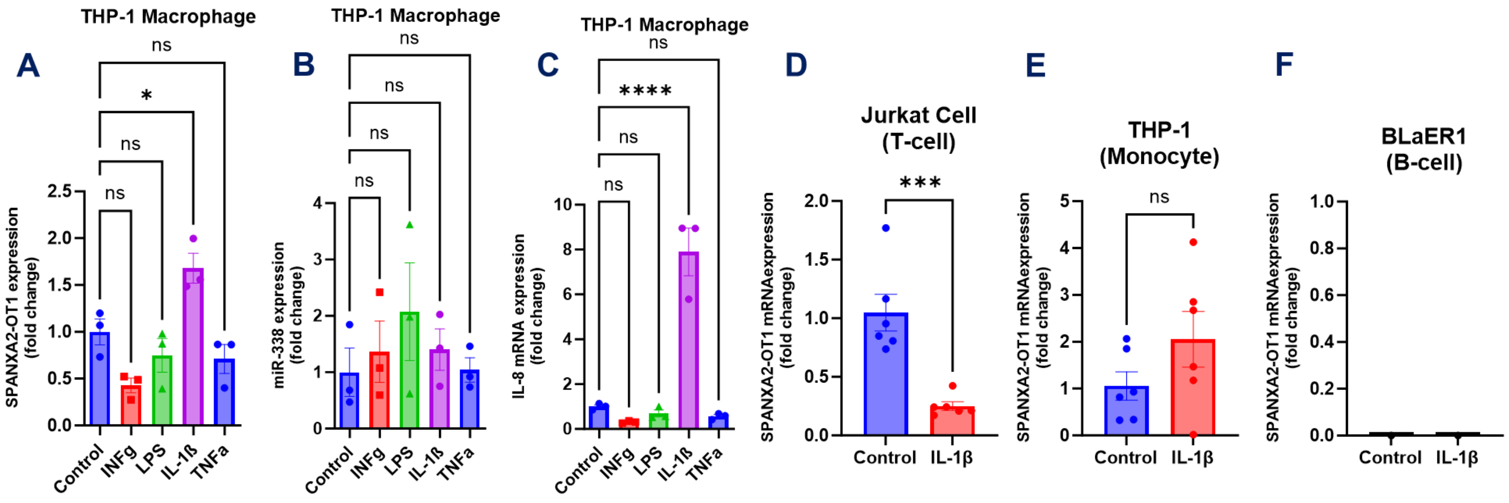


**Figure S14- SPANXA2-OT1 expression in other cell types-** (A) RNA expression of SPANXA2-OT1 after stimulating THP-1 derived macrophages with several inflammatory stimuli (INFγ, IL1β, TNFα, LPS- all in the concentration of 10 ng/ml) for 6 hours. IL1β stimulation significantly increased the expression of SPANXA2-OT1. n=3/group. (B) miRNA-338 expression in THP-1 derived macrophage. N=3/group. (C) mRNA expression of IL-8 in THP-1 derived macrophage. IL1β stimulation significantly increased the expression of IL-8. n=3/group. (D) RNA expression of SPANXA2-OT1 in Jurkat cell (T-cell line) was significantly reduced after IL1β treatment. n=6/group. (E) RNA expression of SPANXA2-OT1 in THP-1 monocyte remained unchanged after IL1β treatment. n=6/group. (F) RNA expression of SPANXA2-OT1 in BLAeR1 cell (B-cell line) was not detected in control or IL1β treatment. n=6/group. Statistical analysis was performed using one-way ANOVA with Tukey's multiple comparisons test (A-C) and unpaired student's T-test (D-E). p-Values are denoted as: ***p <0.0001, **p <0.001, and *p <0.05.

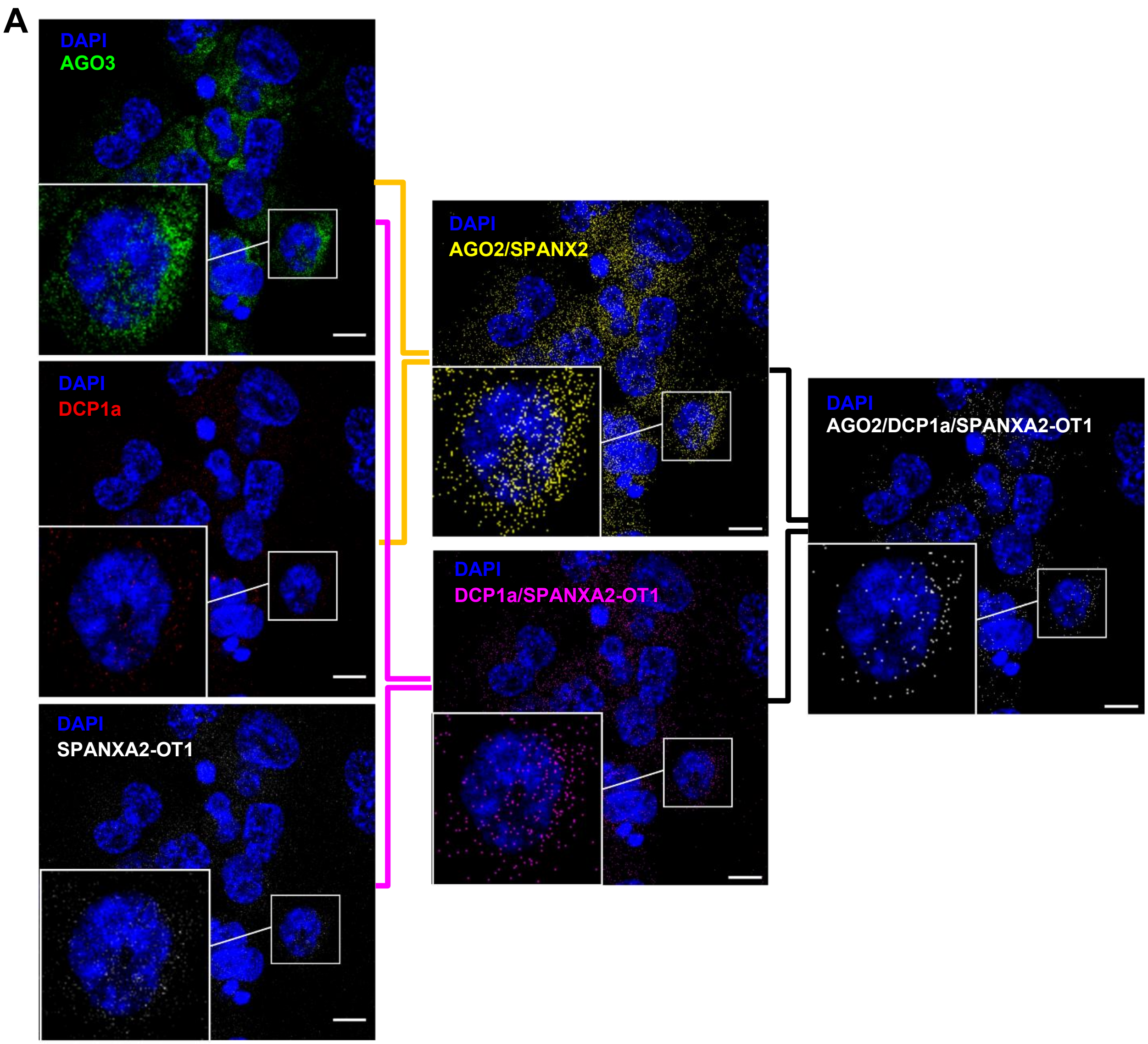
A
DAPI
AGO3
DAPI
DCP1a
DAPI
SPANXA2-OT1
DAPI
AGO2/SPANX2
DAPI
DCP1a/SPANXA2-OT1
DAPI
AGO2/DCP1a/SPANXA2-OT1

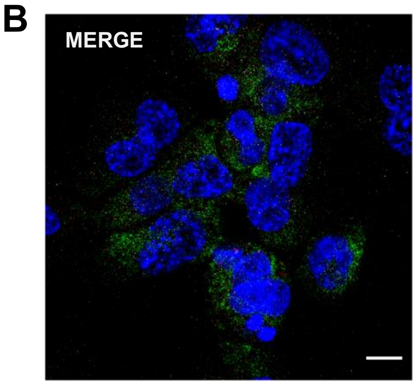
B
MERGE

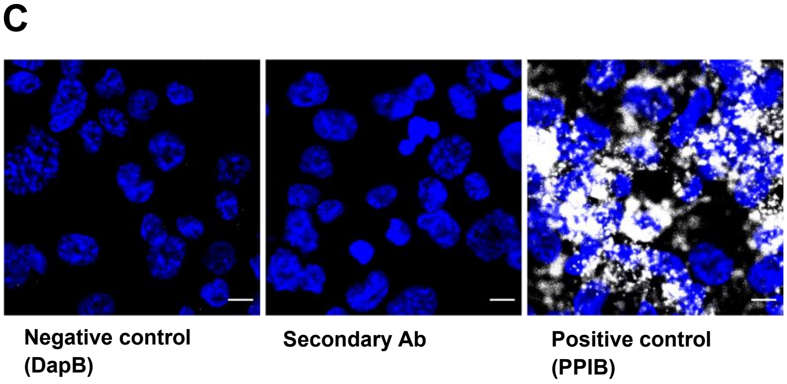
C
Negative control
(DapB)
Secondary Ab
Positive control
(PPIB)

**Figure S15:** Colocalization of SPANXA2-OT1 with AGO2 and DCP1a in THP-1–derived macrophages. (A) Representative confocal images from RNA-protein co-detection using the RNAscope™ assay in THP-1–derived macrophages show cytoplasmic localization of SPANXA2-OT1 (white) along with DCP1a (red), AGO2 (green), and DAPI (blue). Merged panels show colocalization of SPANXA2-OT1 with DCP1a and AGO2. (B) Zoomed-in merged panel from (A) showing overlap of SPANXA2-OT1 with AGO2 and DCP1a. (C) Control images for immunofluorescence using IgG and ISH using negative control (DapB) and positive control (PPIB) probes. Scale bars: 10 μm.

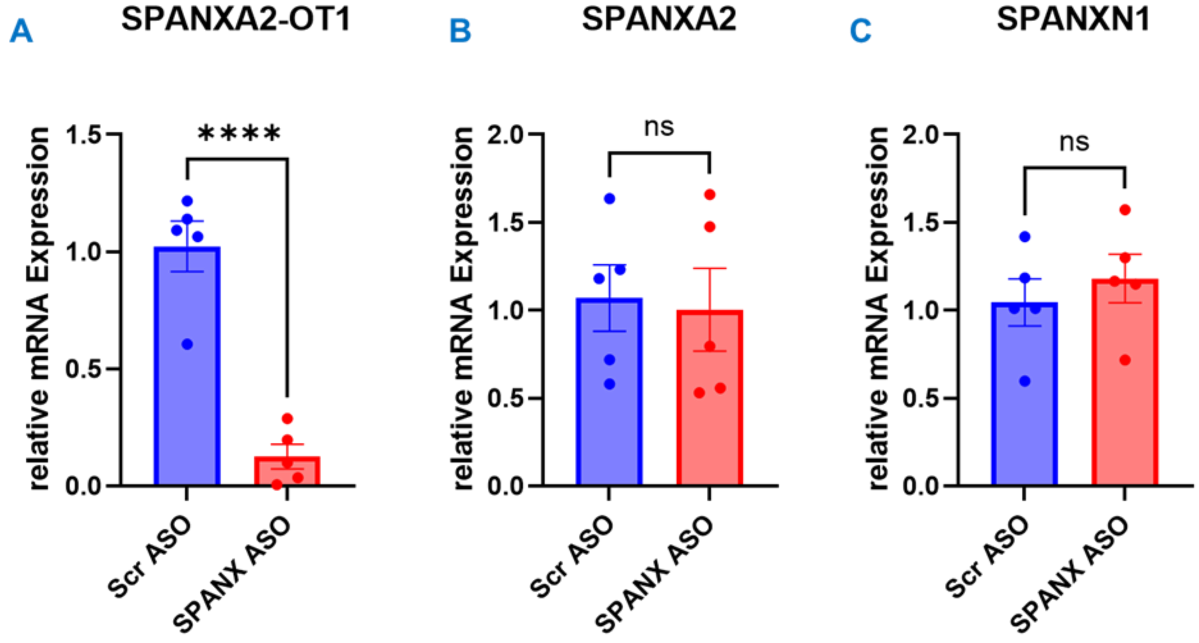


**Figure S16**: Silencing of SPANXA2-OT1 in human primary macrophages using ASO resulted in significantly decreased expression of SPANXA2-OT1 but there were no changes in the expression of its overlapping gene, SPANXA2 and neighboring genes SPANXN1, n=5 PBMC donors. Statistical analysis was performed using unpaired student's T-test. p-Values are denoted as: ***p <0.0001, **p <0.001, and *p <0.05.

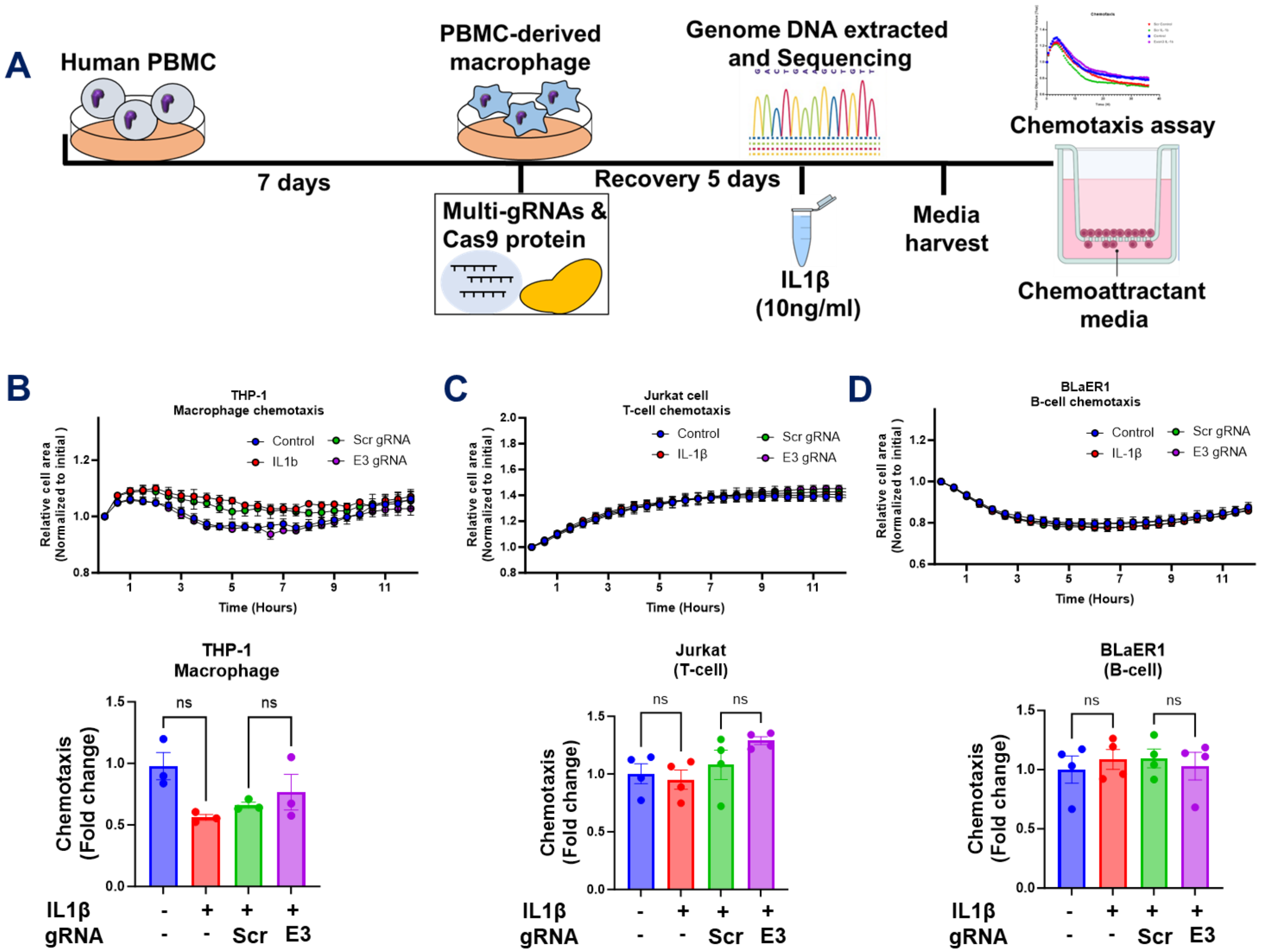


**Figure S17- Chemotaxis assay of immune cells with SPANXA2-OT1 (Exon 3) knockout macrophage supernatant media as chemoattractant.** (A) Schematic of CRISPR/Cas9 editing of SPANXA2-OT1-exon 3 experiment. (B-D) Live cell imaging of cell chemotaxis for 12-hours. THP-1 macrophage (B), n=3/group. Jurkat cell (C), n=4/group. BlaER1 cell (D), n=4/group. Statistical analysis was performed using one-way ANOVA with Tukey's multiple comparisons test. p-Values are denoted as: ***p <0.0001, **p <0.001, and *p <0.05.

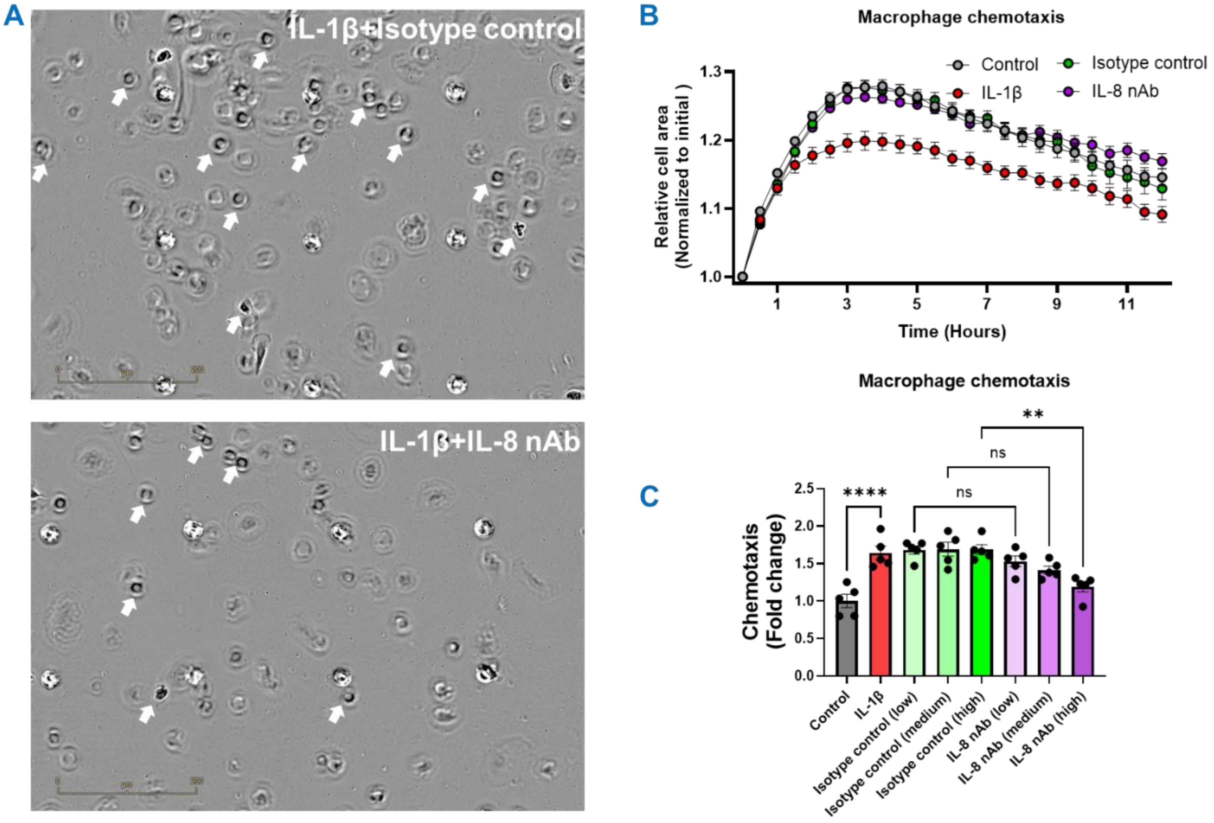


**Figure S18**- Live cell imaging of primary macrophage chemotaxis for 12 hours. IL-8 mediates macrophage chemotaxis downstream of SPANXA2-OT1. (A) Representative live-cell images at 6 hours from a 12-hour chemotaxis assay using human primary macrophages. Arrows indicate macrophages that migrated to the bottom of the matrix gel. (B) Real-time tracking of chemotaxis over 12 hours, showing reduced migration in IL-8–neutralized and SPANXA2-OT1–deleted conditioned media compared to controls. (C) Quantification of chemotaxis in response to conditioned media with IL-8 neutralizing antibody (0.1–1 μg/mL) or isotype controls. Chemotaxis progressively decreased with increasing IL-8 antibody concentration, becoming significant at 1 μg/mL. Data represent mean ± SEM from 5 PBMC donors. Statistical analysis was performed using one-way ANOVA with Tukey's multiple comparisons test. p-Values are denoted as: ***p <0.0001, **p <0.001, and *p <0.05.

| S. No. | GEO accession no. | Platform of dataset | Samples( Control/patients) | Organism | Platform | Model of generating expression summaries | Reference |
|---|---|---|---|---|---|---|---|
| ***mRNA DATASETS*** | | | | | | | |
| **1** | [GSE113079] | GPL20115 | (n=141) 48/93 | Homo Sapiens | Agilent-067406 Human CBC lncRNA + mRNA microarray V4.0 | log2 transformed and quantile normalized | (1) |
| **2** | [GSE98583] | GPL571 | (n=18) 06/12 | Homo Sapiens | [HG-U133A_2] Affymetrix Human Genome U133A 2.0 Array | log2 transformed and quantile normalized | (2) |
| **3** | [GSE56885] | GPL15207 | (n=06) 02/04 | Homo Sapiens | Affymetrix Human Gene Expression Array | log2 transformed and quantile normalized | (--) |
| **4** | [GSE42148] | GPL13607 | (n=24) 11/13 | Homo Sapiens | Agilent-028004 SurePrint G3 Human GE 8x60K Microarray | log2 transformed and quantile normalized | (3) |
| **5** | [GSE34822] | GPL6480 | (n=32) 16/16 | Homo Sapiens | Agilent-014850 Whole Human Genome Microarray 4x44K G4112F | log2 transformed and quantile normalized | (5) |
| **6** | [GSE90074] | GPL6480 | (n=108) 36/72 | Homo Sapiens | Agilent-014850 Whole Human Genome Microarray 4x44K G4112F | log2 transformed and quantile normalized | (7) |
| **7** | [GSE974] | GPL96 | (n=38) 16/22 | Homo Sapiens | [HG-U133A] Affymetrix Human Genome U133A Array | log2 transformed and quantile normalized | (8) |
| **8** | [GSE28829] | GPL570 | (n=29) 13/16 | Homo Sapiens | [HG-U133_Plus_2] Affymetrix Human Genome U133 Plus 2.0 Array | log2 transformed and quantile normalized | (13) |
| ***microRNA (miRNA) DATASETS*** | | | | | | | |
| **1** | [GSE59421] | GPL10850 | (n=70) 37/33 | Homo Sapiens | Agilent-021827 Human miRNA Microarray (V3) (miRBase release 12.0 miRNA ID version) | log2 transformed and quantile normalized | (9) |
| **2** | [GSE105449] | GPL22949 | (n=80) 42/38 | Homo Sapiens | Agilent-021827 Human miRNA Microarray G4470C (miRNA ID version, miRBase v18) | log2 transformed and quantile normalized | (10) |
| ***Long non-coding RNA (lncRNA) DATASET*** | | | | | | | |
| **1** | [GSE113079] | GPL20115 | (n=141) 48/93 | Homo Sapiens | Agilent-067406 Human CBC lncRNA + mRNA microarray V4.0 | log2 transformed and quantile normalized | (1) |

**Table S1-** Details of datasets used for analysis of lncRNA-miRNA-mRNA regulatory triad in CAD

| Entrez ID | Gene symbols | Gene name | CombinedTstat | Adj p-values |
|---|---|---|---|---|
| **Increased DEGs** | | | | |
| **3576** | IL8 | C-X-C Motif Chemokine Ligand 8 | 102.16 | 2.06E-11 |
| **6446** | SGK1 | Serum/Glucocorticoid Regulated Kinase 1 | 96.152 | 1.49E-10 |
| **50486** | G0S2 | G0/G1 Switch 2 | 94.717 | 1.87E-10 |
| **9935** | MAFB | MAF BZIP Transcription Factor B | 85.448 | 7.92E-09 |
| **7305** | TYROBP | TYRO Protein Tyrosine Kinase Binding Protein | 84.608 | 9.10E-09 |
| **929** | CD14 | CD14 Molecule | 83.398 | 1.28E-08 |
| **7852** | CXCR4 | C-X-C Motif Chemokine Receptor 4 | 81.332 | 2.65E-08 |
| **8870** | IER3 | Immediate Early Response 3 | 79.473 | 5.14E-08 |
| **639** | PRDM1 | PR/SET Domain 1 | 76.617 | 1.39E-07 |
| **9308** | CD83 | CD83 Molecule | 75.49 | 2.03E-07 |
| **Decreased DEGs** | | | | |
| **7220** | TRPC1 | Transient Receptor Potential Cation Channel Subfamily C Member 1 | -77.24 | 1.18E-07 |
| **23142** | DCUN1D4 | Defective In Cullin Neddylation 1 Domain Containing 4 | -73.953 | 3.07E-07 |
| **9532** | BAG2 | BCL2 Associated Athanogene 2 | -70.381 | 1.11E-06 |
| **54980** | C2orf42 | Chromosome 2 Open Reading Frame 42 | -62.793 | 1.12E-05 |
| **55197** | RPRD1A | Regulation Of Nuclear Pre-MRNA Domain Containing 1A | -60.496 | 2.10E-05 |
| **55022** | PID1 | Phosphotyrosine Interaction Domain Containing 1 | -59.378 | 2.82E-05 |
| **157567** | ANKRD46 | Ankyrin Repeat Domain 46 | -59.226 | 2.95E-05 |
| **10516** | FBLN5 | Fibulin 5 | -58.618 | 3.49E-05 |
| **54664** | TMEM106B | Transmembrane Protein 106B | -57.549 | 4.98E-05 |
| **22800** | RRAS2 | RAS Related 2 | -57.393 | 5.04E-05 |

**Table S2-** Top 20 differentially expressed mRNAs in CAD

| miRNAs | Individual dataset fold change | | Meta-analysis results | |
|---|---|---|---|---|
| | GSE105449 | GSE59421 | CombinedTstat | CombinedPval |
| **Increased** | | | | |
| **hsa-miR-1246** | 0.13302 | 0.61803 | 21.269 | 0.044534 |
| **hsa-miR-598** | -0.038391 | 0.21536 | 14.509 | 0.017183 |
| **hsa-miR-199a-5p** | 0.013767 | 0.29428 | 12.89 | 0.017183 |
| **hsa-miR-1207-5p** | 0.045664 | 0.22446 | 12.793 | 0.017183 |
| **hsa-let-7c** | 0.030737 | 0.11991 | 12.677 | 0.017183 |
| **hsa-miR-1308** | 0.026287 | 0.20955 | 12.59 | 0.017183 |
| **hsa-miR-210** | 0.059158 | 0.21013 | 12.164 | 0.017183 |
| **hsa-miR-1290** | -0.0096654 | 0.091572 | 12.134 | 0.017183 |
| **hsa-miR-542-5p** | 0.11531 | 0.24229 | 11.411 | 0.017736 |
| **hsa-miR-542-3p** | 0.18116 | 0.16346 | 11.281 | 0.017855 |
| **Decreased** | | | | |
| **hsa-miR-376c** | -0.29712 | -0.35747 | -18.435 | 0.07333 |
| **hsa-miR-543** | -0.17642 | -0.022534 | -17.746 | 0.07333 |
| **hsa-miR-1** | -0.1954 | -0.28134 | -15.749 | 0.013413 |
| **hsa-miR-495** | -0.14219 | -0.16698 | -13.3 | 0.017183 |
| **hsa-miR-337-5p** | -0.034278 | -0.19495 | -12.262 | 0.017183 |
| **hsa-miR-654-3p** | -0.16898 | -0.25956 | -12.134 | 0.017183 |
| **hsa-miR-1234** | -0.064874 | -0.2679 | -12.073 | 0.017183 |
| **hsa-miR-1180** | 0.044608 | -0.17974 | -12.008 | 0.017183 |
| **hsa-miR-376b** | -0.096494 | -0.13146 | -11.794 | 0.017725 |
| **hsa-miR-338-3p** | -0.13715 | -0.055041 | -11.516 | 0.017736 |

**Table S3-** Top 20 differentially expressed miRNAs in CAD

| | Term/ID | p-value | Enrichment library | miRNAs/precursors |
|---|---|---|---|---|
| **Enriched terms** | | | | |
| Eicosanoid Synthesis | WP167 | 0.014391 | Pathways (miRWalk) | hsa-miR-28-3p; hsa-miR-146b-5p; hsa-miR-320c; hsa-miR-320a; hsa-miR-423-5p; hsa-miR-361-5p; hsa-miR-484; hsa-miR-199a-5p |
| blood vessel remodeling | GO0001974 | 0.030576 | Gene Ontology (miRWalk) | hsa-miR-125a-5p; hsa-miR-199b-5p; hsa-miR-940; hsa-miR-324-5p; hsa-miR-296-5p; hsa-miR-320a; hsa-miR-140-3p; hsa-miR-142-3p; hsa-miR-484; hsa-miR-199a-5p |
| lipid binding | GO0008289 | 0.011378 | Gene Ontology (miRWalk) | hsa-miR-28-3p; hsa-miR-125a-5p; hsa-miR-146b-5p; hsa-miR-330-3p; hsa-miR-423-3p; hsa-miR-331-3p; hsa-miR-320b; hsa-miR-107; hsa-miR-324-3p; hsa-miR-361-3p; hsa-miR-548d-5p; hsa-miR-320c; hsa-miR-296-5p; hsa-miR-320a; hsa-miR-423-5p; hsa-miR-140-3p; hsa-miR-361-5p; hsa-miR-142-3p; hsa-miR-484 |
| lipid storage | GO0019915 | 0.029816 | Gene Ontology (miRWalk) | hsa-miR-324-3p; hsa-miR-484; hsa-miR-199a-5p |
| macrophage activation | GO0042116 | 0.042035 | Gene Ontology (miRWalk) | hsa-miR-423-5p; hsa-miR-484 |
| positive regulation of vasoconstriction | GO0045907 | 0.040205 | Gene Ontology (miRWalk) | hsa-miR-429; hsa-miR-28-3p; hsa-miR-146b-5p; hsa-miR-320c; hsa-miR-320a; hsa-miR-423-5p; hsa-miR-361-5p; hsa-miR-484; hsa-miR-199a-5p |
| positive regulation of natural killer cell mediated cytotoxicity | GO0045954 | 0.009493 | Gene Ontology (miRWalk) | hsa-miR-324-3p; hsa-miR-320a; hsa-miR-142-3p; hsa-miR-484 |
| positive regulation of smooth muscle contraction | GO0045987 | 0.0298 | Gene Ontology (miRWalk) | hsa-miR-28-3p; hsa-miR-146b-5p; hsa-miR-331-3p; hsa-miR-324-3p; hsa-miR-320c; hsa-miR-320a; hsa-miR-423-5p; hsa-miR-361-5p; hsa-miR-484; hsa-miR-199a-5p |
| calcium ion homeostasis | GO0055074 | 0.00621 | Gene Ontology (miRWalk) | hsa-miR-484; hsa-miR-199a-5p |
| lipid homeostasis | GO0055088 | 0.023907 | Gene Ontology (miRWalk) | has-miR-765; has-miR-107; has-miR-324-3p; has-miR-362-3p; has-miR-320a; has-miR-484 |
| **Associated enriched diseases** | | | | |
| dilated cardiomyopathy deregulated | - | 0.040378 | Diseases (published studies about miRNA profiles from peripheral blood) | hsa-miR-770-5p; hsa-miR-28-5p; hsa-miR-501-5p; hsa-miR-188-3p; hsa-miR-595; hsa-miR-628-3p; hsa-miR-582-5p; hsa-miR-421; hsa-miR-485-3p; hsa-miR-940; hsa-miR-362-5p; hsa-miR-324-5p; hsa-miR-520d-3p; hsa-miR-1294; hsa-miR-142-5p; hsa-miR-296-5p; hsa-miR-320a; hsa-miR-1305; hsa-miR-140-3p; hsa-miR-139-3p; hsa-miR-486-3p; hsa-miR-1225-5p; hsa-miR-484; hsa-miR-1207-5p; hsa-miR-1246 |
| Neoplasms | - | 0.014303 | Diseases (miRWalk) | hsa-miR-654-3p; hsa-miR-299-5p; hsa-miR-326; hsa-miR-28-5p; hsa-miR-429; hsa-miR-483-5p; hsa-miR-885-5p; hsa-miR-125a-5p; hsa-miR-409-3p; hsa-miR-491-5p; hsa-miR-127-3p; hsa-miR-140-5p; hsa-miR-342-3p; hsa-miR-146b-5p; hsa-miR-193a-5p; hsa-miR-199b-5p; hsa-miR-330-3p; hsa-miR-582-5p; hsa-miR-423-3p; hsa-miR-483-3p; hsa-miR-193a-3p; hsa-miR-199a-3p; hsa-miR-572; hsa-miR-331-3p; hsa-miR-491-3p; hsa-miR-324-5p; hsa-miR-107; hsa-miR-362-3p; hsa-miR-296-5p; hsa-miR-337-3p |

**Table S4-** DE miRNA enrichment analysis using miEAA tool.

| lncRNA ID | Sequence Name | Location | Length | Strand | Exone number | Type | log2Fold Change | Adj p-val |
|---|---|---|---|---|---|---|---|---|
| **Increased** | | | | | | | | |
| **GS1-204I12.1** | NR_110793.1 | chr1: 185558379-185628488 | 604 | (-) | 4 | lincRNA | 1.6586 | 1.79E-18 |
| **FAM155A-IT1** | NR_046848.1 | chr13:107787361-107835458 | 1807 | (-) | 2 | Intronic lncRNA | 1.5207 | 1.64E-20 |
| **CTD-2231E14.4** | NR_146442.1 | chrX:72744111-72782921 | 446 | (-) | 3 | lincRNA | 1.2651 | 1.03E-14 |
| **GS1-18A18.1** | NR_038994.1 | chr7: 54556970-54571726 | 3031 | (+) | 4 | Overlapping lncRNA | 1.2041 | 1.54E-08 |
| **DIAPH3-AS2** | NR_046540.1 | chr13: 60144698-60153505 | 577 | (+) | 3 | Antisense LncRNA | 1.1603 | 7.84E-12 |
| **Decreased** | | | | | | | | |
| **CTD-2532D12.4** | NR_164140.1 | chr17: 73737854-73756557 | 1140 | (-) | 6 | Uncharacterized LncRNA | -1.8462 | 4.49E-26 |
| **ARMC2-AS1** | NR_104137.1 | chr6: 108908140-108924103 | 717 | (-) | 3 | Antisense LncRNA | -1.4485 | 1.38E-27 |
| **LINC00184** | NR_033927.1 | chr1: 234629311-234634780 | 2298 | (+) | 2 | lincRNA | -1.4332 | 1.92E-27 |
| **RBM26-AS1** | NR_038991.1 | chr13: 79406309-79424336 | 2484 | (+) | 7 | Antisense LncRNA | -1.3715 | 1.95E-26 |
| **ZNF32-AS2** | NR_047558.1 | chr10: 43645942-43648019 | 905 | (+) | 3 | Antisense LncRNA | -1.3700 | 1.67E-31 |

**Table S5-** Top ten differentially expressed lncRNAs in CAD

| lncRNA gene ID | NONCODE lncRNA ID | Locus | Length | Strand | Exon number | CNCI score | log2FoldChange | padj |
|---|---|---|---|---|---|---|---|---|
| **Module 1 (Blue)** | | | | | | | | |
| **HIF1A-AS2** | NONHSAT037281.2 | chr14: 61747371-61748565 | 1194 | (-) | 1 | -0.0004096 | -0.51259 | 6.77E-05 |
| **SPANXA2-OT1** | NONHSAT138833.2 | chrX: 141502848-141649939 | 1698 | (+) | 4 | -0.0659456 | 0.73967 | 6.73E-11 |
| **LINC00211** | NONHSAT070144.2 | chr2: 37820464-37829398 | 2202 | (-) | 3 | -0.0577536 | -0.5427 | 2.96E-08 |
| **CSNK1G2-AS1** | NONHSAT060381.2 | chr19: 1952526-1954549 | 1313 | (-) | 3 | -0.0552960 | 0.59499 | 3.81E-13 |
| **KDM4A-AS1** | NONHSAT002817.2 | chr1: 43699723-43707341 | 1574 | (-) | 5 | -0.05406 | -0.50696 | 5.15E-11 |
| **FAM225A** | NONHSAT134170.2 | chr9: 113112895-113119846 | 5802 | (+) | 3 | -0.0368640 | 0.70369 | 8.67E-06 |
| **Module 2 (Brown)** | | | | | | | | |
| **GAS6-AS1** | NONHSAT035379.2 | chr13: 113815609-113842841 | 895 | (+) | 5 | -0.0409600 | -0.94649 | 1.74E-19 |
| **ADPGK-AS1** | NONHSAT047125.2 | chr15: 72782834-72798199 | 2730 | (+) | 3 | -0.1101824 | -1.2352 | 1.65E-23 |
| **RXFP1** | NONHSAT098999.2 | chr4: 158521713-158653369 | 3812 | (+) | 16 | -0.0327680 | N/A | N/A |
| **SIGMAR1** | NONHSAT220636.1 | chr9: 34634721-34637826 | 1598 | (-) | 4 | -0.1040 | N/A | N/A |
| **LINC00471** | NONHSAT077373.2 | chr2: 231508425-231514377 | 1320 | (-) | 3 | -0.0274432 | -0.95934 | 1.25E-28 |
| **LINC00582** | NONHSAT010185.2 | chr1: 231591291-231612090 | 597 | (-) | 2 | -0.0778240 | -0.96841 | 3.74E-25 |

| | | | | | | | | |
|---|---|---|---|---|---|---|---|---|
| **LINC00698** | NONHSAT090200.2 | chr3: 63102687-63125061 | 1835 | (+) | 7 | -0.0274432 | -0.89527 | 8.81E-20 |
| **DNAJC19** | NONHSAT093459.2 | chr3: 180983709-180989774 | 1247 | (-) | 3 | -0.0204800 | N/A | N/A |
| **SNHG7** | NONHSAT135577.2 | chr9: 136724593-136728184 | 853 | (-) | 5 | -0.0921600 | -0.5781 | 6.67E-10 |
| **FAM27E3** | NONHSAT220772.1 | chr9: 67717497-67719179 | 1507 | (-) | 2 | -0.0380928 | N/A | N/A |
| **CTC-338M12.4** | NONHSAT105938.2 | chr5: 181246540-181257586 | 1013 | (+) | 3 | -0.0430080 | -0.58216 | 4.02E-23 |
| **RSRC2** | NONHSAT164132.1 | chr12: 122504642-122527013 | 2371 | (-) | 10 | -0.0794624 | N/A | N/A |
| **Module 3 (Yellow)** | | | | | | | | |
| **CACNG6** | NONHSAT067856.2 | chr19: 53991148-54012666 | 840 | (+) | 3 | -0.0778240 | N/A | N/A |
| **ST7-AS1** | NONHSAT122914.2 | chr7: 116952445-116954334 | 1889 | (-) | 1 | -0.0651264 | 0.54483 | 4.77E-06 |
| **SYNJ2-IT1** | NONHSAT115729.2 | chr6: 158001106-158002383 | 599 | (+) | 3 | -0.0274432 | -0.61994 | 0.000106 |
| **FAM95B1** | NONHSAT131301.2 | chr9: 40323570-40329220 | 1543 | (+) | 5 | -0.0290816 | N/A | N/A |
| **LINC00469** | NONHSAT055739.2 | chr17: 73749269-73828537 | 1549 | (-) | 4 | -0.0643072 | -0.57188 | 6.02E-23 |
| **COL25A1** | NONHSAT200278.1 | chr4: 108810720-109302367 | 4630 | (-) | 36 | -0.0487424 | N/A | N/A |
| **STARD4-AS1** | NONHSAT103194.2 | chr5: 111512225-111739726 | 5525 | (+) | 7 | -0.0897024 | -0.50831 | 2.11E-05 |
| **TMPO-AS1** | NONHSAT030138.2 | chr12: 98512972-98516454 | 3389 | (-) | 2 | -0.0897024 | -0.54251 | 1.00E-08 |
| **LINC00844** | NONHSAT013506.2 | chr10: 58999517-59001617 | 659 | (+) | 2 | -0.0004096 | 0.88421 | 1.22E-12 |
| **SURF6** | NONHSAT135301.2 | chr9: 133330706-133336210 | 2273 | (-) | 5 | -0.2363392 | N/A | N/A |

| | | | | | | | | |
|---|---|---|---|---|---|---|---|---|
| **HOXA11-AS** | NONHSAT119704.2 | chr7: 27185396-27189293 | 1560 | (+) | 2 | -0.0557056 | -0.54507 | 8.19E-07 |
| **TGDS** | NONHSAT167098.1 | chr13: 94574053-94596275 | 1829 | (-) | 11 | -0.0147456 | N/A | N/A |
| **LRP4-AS1** | NONHSAT021205.2 | chr11: 46846410-46874416 | 1540 | (+) | 3 | -0.0110592 | -0.50803 | 5.48E-05 |
| **HCFC1-AS1** | NONHSAT223128.1 | chrX: 153968764-153970091 | 786 | (+) | 3 | -0.0561152 | -0.67167 | 1.50E-09 |
| **Module 4 (Red)** | | | | | | | | |
| **MAPKAPK5-AS1** | NONHSAT030758.2 | chr12: 111839763-111842902 | 2290 | (-) | 2 | -0.1306624 | 0.75507 | 3.19E-16 |
| **ATP6V0E2-AS1** | NONHSAT124073.2 | chr7: 149863710-149873869 | 7059 | (-) | 4 | -0.1019904 | 0.93141 | 6.30E-11 |
| **LINC00445** | NONHSAT033186.2 | chr13: 35697531-35699203 | 570 | (+) | 2 | -0.0212992 | 0.83073 | 7.25E-11 |
| **DNAJC5G** | NONHSAT181470.1 | chr2: 27275420-27281431 | 1367 | (+) | 4 | -0.1802240 | N/A | N/A |
| **CALML3-AS1** | NONHSAT011157.2 | chr10: 5514243-5526246 | 3709 | (-) | 4 | -0.0180224 | 0.53012 | 8.15E-11 |
| **Module 5 (Cyan)** | | | | | | | | |
| **DOCK9-AS1** | NONHSAT034894.2 | chr13: 98832083-98834629 | 392 | (+) | 3 | -0.1544192 | -0.5204 | 1.26E-07 |
| **IDH1-AS1** | NONHSAT076636.2 | chr2: 208255232-208256194 | 491 | (+) | 2 | -0.0053248 | 0.54883 | 1.82E-14 |
| **LINC00599** | NONHSAT124969.2 | chr8: 9899720-9903378 | 3314 | (-) | 3 | -0.0606208 | -0.53051 | 4.04E-10 |
| **TTTY5** | NONHSAT139679.2 | chrY: 22296797-22298876 | 1562 | (-) | 2 | -0.0004096 | -1.0473 | 2.83E-24 |
| **LINC00707** | NONHSAT011260.2 | chr10: 6779578-6842901 | 3084 | (+) | 5 | -0.0122880 | -0.8724 | 6.71E-10 |
| **Module 6 (Green)** | | | | | | | | |
| **NAALADL2-AS3** | NONHSAT093297.2 | chr3: 175079306-175115242 | 413 | (-) | 4 | -0.0004096 | -0.57411 | 1.27E-06 |

| | | | | | | | | |
|---|---|---|---|---|---|---|---|---|
| **NLGN4Y-AS1** | NONHSAT139520.2 | chrY: 14793641-14804033 | 532 | (-) | 4 | -0.3604480 | -0.57092 | 0.000154 |
| **Module 7 (Purple)** | | | | | | | | |
| **KLHDC9** | NONHSAT007100.2 | chr1: 161098360-161100348 | 1107 | (+) | 5 | -0.0966656 | N/A | N/A |

**Table S6-** Candidate lncRNAs prioritized from modules selected from WGCNA algorithm. Coding-Non-Coding Index (CNCI).

| LncRNA ID | CP SCore | PFam | SMART | SUPFAM | C/NC |
|---|---|---|---|---|---|
| **HIF1A-AS2** | 0.0162251 | No Domain | No Domain | No Domain | NC |
| **SPANXA2-OT1** | 0.241365 | No Domain | No Domain | No Domain | NC |
| **LINC00211** | 0.0534184 | No Domain | No Domain | No Domain | NC |
| **CSNK1G2-AS1** | 0.168779 | No Domain | No Domain | No Domain | NC |
| **KDM4A-AS1** | 0.0449509 | No Domain | No Domain | No Domain | NC |
| **FAM225A** | 0.662405 | No Domain | No Domain | No Domain | C |

**Table S7-** Coding potential analysis of lncRNAs from the top (blue) module 1.

| Label | Strand | Frame | Start | Stop | Length (nt \| aa) | Nucleotide Sequence | Coding potential score (C/NC) |
|---|---|---|---|---|---|---|---|
| **ORF1** | + | 1 | 784 | 861 | 78 \| 25 | >lcl\|ORF1 CDS<br>ATGTATTTCCTGAGTATAGATACCAACTCTCCTGATTTCCTCAGGACTAA<br>AGGAGTTTCTGGGACAAGAAAATTATAG | 0.008974 (NC) |
| **ORF2** | + | 1 | 910 | 987 | 78 \| 25 | >lcl\|ORF2 CDS<br>ATGCATGAGGATGTGTATATATGCATATTTTTATGTATGCTAATTCATAT<br>TATAATTTACATACCATATTTATACTAG | 0.006647 (NC) |
| **ORF3** | + | 2 | 542 | 643 | 102 \| 33 | >lcl\|ORF3 CDS<br>ATGCTGAAAATGAAAGTCTCTTCTGATATGTTCACCAAAGAAAGCGATCC<br>CTTAGCCCAGATCTTGGCAAGAACCAGTGTCAAAGGGATGTTCATCCAGT<br>AA | 0.01736 (NC) |
| **ORF4** | + | 3 | 99 | 497 | 399 \| 132 | >lcl\|ORF4 CDS<br>ATGGCGAGCCCCTTTGGGCGCCTCACTGATCAGAAGGGCAGAGGACACCC<br>TGCTGGATCTGGAGGAGTGGAAGTCAATGGCGGGTCTGCGAGGGCGGCGT<br>TCAGCGGTGGTGGACGGAGGGTGTTGAGCGGTGGTGGACGGTGGTGGACG<br>ACGGCCTTCGGCGGTGGTGGACGGACGGCCTTCGGCGTTGGTGGACGGAC<br>GGCCTTCGGCGGTGGTGGACGGACGGCCTTCGGCGGTGGTGGACGGACGG<br>CCTTCGGCGTTGGTGGACGGACGGCTTTCGGCGGTGGTGAACGAGTCTCA<br>CTCTTGTCACCAGGCTGGAGTGCATTGGCGCGATCTTGGCTCACTGCATC<br>CTCTGCCTCCCGGGTTCAAGCGATTCTCCTGCCTCAGCCTCCCGAGTAG | 0.454271 (NC) |
| **ORF5** | + | 3 | 930 | 1289 | 360 \| 119 | >lcl\|ORF5 CDS<br>ATGCATATTTTTATGTATGCTAATTCATATTATAATTTACATACCATATT<br>TATACTAGGAATCAGGTGTGTTAAATCCCATGATACACATATGTTTATAC<br>TCCAAAATCTATGTAATTACCATCTATTTCTAAAAAACATTCTAGAAACA<br>AAAGTAATTATGAAAGGCTATTTTACTATATTTTGGACCATATATCATCA<br>TACCATATTTAGGACCTCATTTGTTAAGGCTAACTACCTGAATATCTTGA<br>GCAGCCACAAGTTTAGCCCATTTCATGGGACTCATTATAGCAAGTGTTCA<br>TTTCTCCCACCCTCTGTTCATGTCCCCAGTTATATGAACAGGAATTTTTT<br>GTTAGAATAG | 0.533221 (C) |
| **ORF6** | + | 3 | 1311 | 1391 | 81 \| 26 | >lcl\|ORF6 CDS<br>ATGTTACAGATAATGGAAAAGCTTGCATACATGGCCACTTTGGAGGATAG<br>ACACTTTTCCTCATTTAACAAAATAGTGTGA | 0.007853 (NC) |
| **ORF7** | - | 1 | 636 | 439 | 198 \| 65 | >lcl\|ORF7 CDS<br>ATGAACATCCCTTTGACACTGGTTCTTGCCAAGATCTGGGCTAAGGGATC<br>GCTTTCTTTGGTGAACATATCAGAAGAGACTTTCATTTTCAGCATTCCTC<br>ACATCTGGTCTGATCTTTGTCCAGCACCTGTAGTTCCAGCTACTCGGGAG<br>GCTGAGGCAGGAGAATCGCTTGAACCCGGGAGGCAGAGGATGCAGTGA | 0.150086 (NC) |
| **ORF8** | - | 1 | 423 | 199 | 225 \| 74 | >lcl\|ORF8 CDS<br>ATGCACTCCAGCCTGGTGACAAGAGTGAGACTCGTTCACCACCGCCGAAA<br>GCCGTCCGTCCACCAACGCCGAAGGCCGTCCGTCCACCACCGCCGAAGGC<br>CGTCCGTCCACCACCGCCGAAGGCCGTCCGTCCACCAACGCCGAAGGCCG<br>TCCGTCCACCACCGCCGAAGGCCGTCGTCCACCACCGTCCACCACCGCTC<br>AACACCCTCCGTCCACCACCGCTGA | 0.037489 (NC) |
| **ORF9** | - | 3 | 1405 | 1259 | 147 \| 48 | >lcl\|ORF9 CDS<br>ATGGTCTTATGTATTCACACTATTTTGTTAAATGAGGAAAAGTGTCTATC<br>CTCCAAAGTGGCCATGTATGCAAGCTTTTCCATTATCTGTAACATAGCAA<br>AAATAAAGAATTTGTTCTATTCTAACAAAAAATTCCTGTTCATATAA | 0.027698 (NC) |

**Table S8**- Potential ORFs on SPANXA2-OT1 and their coding potential score. C denotes coding and NC denotes noncoding.

## References


1. Moher D, Liberati A, Tetzlaff J, Altman DG, and Group P. Preferred reporting items for systematic reviews and meta-analyses: the PRISMA statement. *BMJ.* 2009;339:b2535.
2. Xia J, Fjell CD, Mayer ML, Pena OM, Wishart DS, and Hancock RE. INMEX--a web-based tool for integrative meta-analysis of expression data. *Nucleic Acids Res.* 2013;41(Web Server issue):W63-70.

3. Johnson WE, Li C, and Rabinovic A. Adjusting batch effects in microarray expression data using empirical Bayes methods. *Biostatistics.* 2007;8(1):118-27.
4. Chen EY, Tan CM, Kou Y, Duan Q, Wang Z, Meirelles GV, et al. Enrichr: interactive and collaborative HTML5 gene list enrichment analysis tool. *BMC Bioinformatics.* 2013;14:128.
5. Vijay A, Jha PK, Garg I, Sharma M, Ashraf MZ, and Kumar B. micro-RNAs dependent regulation of DNMT and HIF1alpha gene expression in thrombotic disorders. *Sci Rep.* 2019;9(1):4815.
6. Backes C, Khaleeq QT, Meese E, and Keller A. miEAA: microRNA enrichment analysis and annotation. *Nucleic Acids Res.* 2016;44(W1):W110-6.
7. Ritchie ME, Phipson B, Wu D, Hu Y, Law CW, Shi W, et al. limma powers differential expression analyses for RNA-sequencing and microarray studies. *Nucleic Acids Res.* 2015;43(7):e47.
8. Zhao Y, Li H, Fang S, Kang Y, Wu W, Hao Y, et al. NONCODE 2016: an informative and valuable data source of long non-coding RNAs. *Nucleic Acids Res.* 2016;44(D1):D203-8.
9. Langfelder P, and Horvath S. WGCNA: an R package for weighted correlation network analysis. *BMC Bioinformatics.* 2008;9:559.
10. Hu Z, Snitkin ES, and DeLisi C. VisANT: an integrative framework for networks in systems biology. *Brief Bioinform.* 2008;9(4):317-25.
11. Ke L, Yang DC, Wang Y, Ding Y, and Gao G. AnnoLnc2: the one-stop portal to systematically annotate novel lncRNAs for human and mouse. *Nucleic Acids Res.* 2020;48(W1):W230-w8.
12. Agarwal V, Bell GW, Nam JW, and Bartel DP. Predicting effective microRNA target sites in mammalian mRNAs. *Elife.* 2015;4.
13. Lorenz R, Bernhart SH, Honer Zu Siederdissen C, Tafer H, Flamm C, Stadler PF, et al. ViennaRNA Package 2.0. *Algorithms Mol Biol.* 2011;6:26.
14. Kruger J, and Rehmsmeier M. RNAhybrid: microRNA target prediction easy, fast and flexible. *Nucleic Acids Res.* 2006;34(Web Server issue):W451-4.
15. Kong L, Zhang Y, Ye ZQ, Liu XQ, Zhao SQ, Wei L, et al. CPC: assess the protein-coding potential of transcripts using sequence features and support vector machine. *Nucleic Acids Res.* 2007;35(Web Server issue):W345-9.
16. Finn RD, Mistry J, Tate J, Coggill P, Heger A, Pollington JE, et al. The Pfam protein families database. *Nucleic Acids Res.* 2010;38(Database issue):D211-22.
17. Nakano T, Katsuki S, Chen M, Decano JL, Halu A, Lee LH, et al. Uremic Toxin Indoxyl Sulfate Promotes Proinflammatory Macrophage Activation Via the Interplay of OATP2B1 and Dll4-Notch Signaling. *Circulation.* 2019;139(1):78-96.
18. Lee LH, Halu A, Morgan S, Iwata H, Aikawa M, and Singh SA. XINA: A Workflow for the Integration of Multiplexed Proteomics Kinetics Data with Network Analysis. *J Proteome Res.* 2019;18(2):775-81.
19. Cornwell M, Vangala M, Taing L, Herbert Z, Köster J, Li B, et al. VIPER: Visualization Pipeline for RNA-seq, a Snakemake workflow for efficient and complete RNA-seq analysis. *BMC Bioinformatics.* 2018;19(1):135.
