# Supplementary material for: A systems approach to target discovery identifies the role of lncRNA-SPANXA2-OT1 in macrophage chemotaxis
Source: JCI Insight. 2025 Oct 9;10(21):e191274. doi: 10.1172/jci.insight.191274 (PMC12643516; doi:10.1172/jci.insight.191274)

Figure 2E

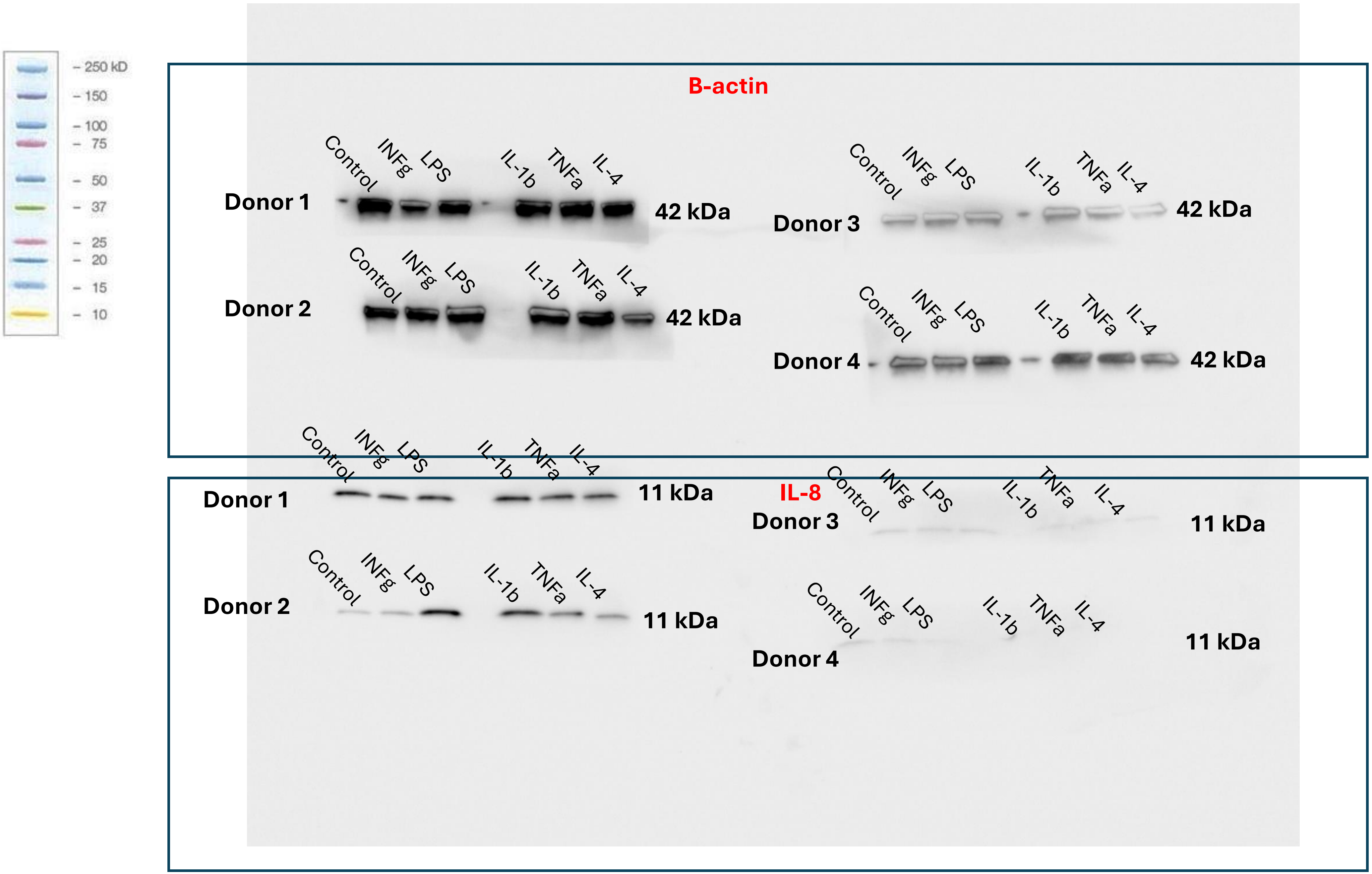
250 kD
150
100
75
50
37
25
20
15
10
B-actin
Donor 1
Control
INFg
LPS
IL-1b
TNFa
IL-4
42 kDa
Donor 2
42 kDa
Donor 3
42 kDa
Donor 4
42 kDa
IL-8
Donor 1
11 kDa
Donor 2
11 kDa
Donor 3
11 kDa
Donor 4
11 kDa

Figure 3H & 5D

A

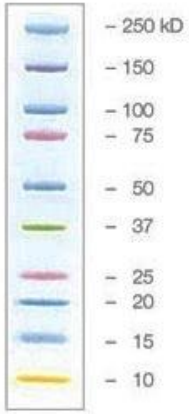

B

Well#	Treatment
1	Control
2	IL1β
3	IL1β+NC-miR
4	IL1β+miR-338 mimic
5	Control
6	IL1β
7	IL1β+NC-ASO
8	IL1β+SPANXA2-OT1 ASO

Uncropped membranes used for western blot analysis. **A.** Protein ladder scale (kaleidoscope, BioRad). **B.** Sample description for each well, each condition included three independent donors. **C.** Chemiluminescence blot for IL-8 and beta-actin blot. **D.** Colorimetric blot.

C

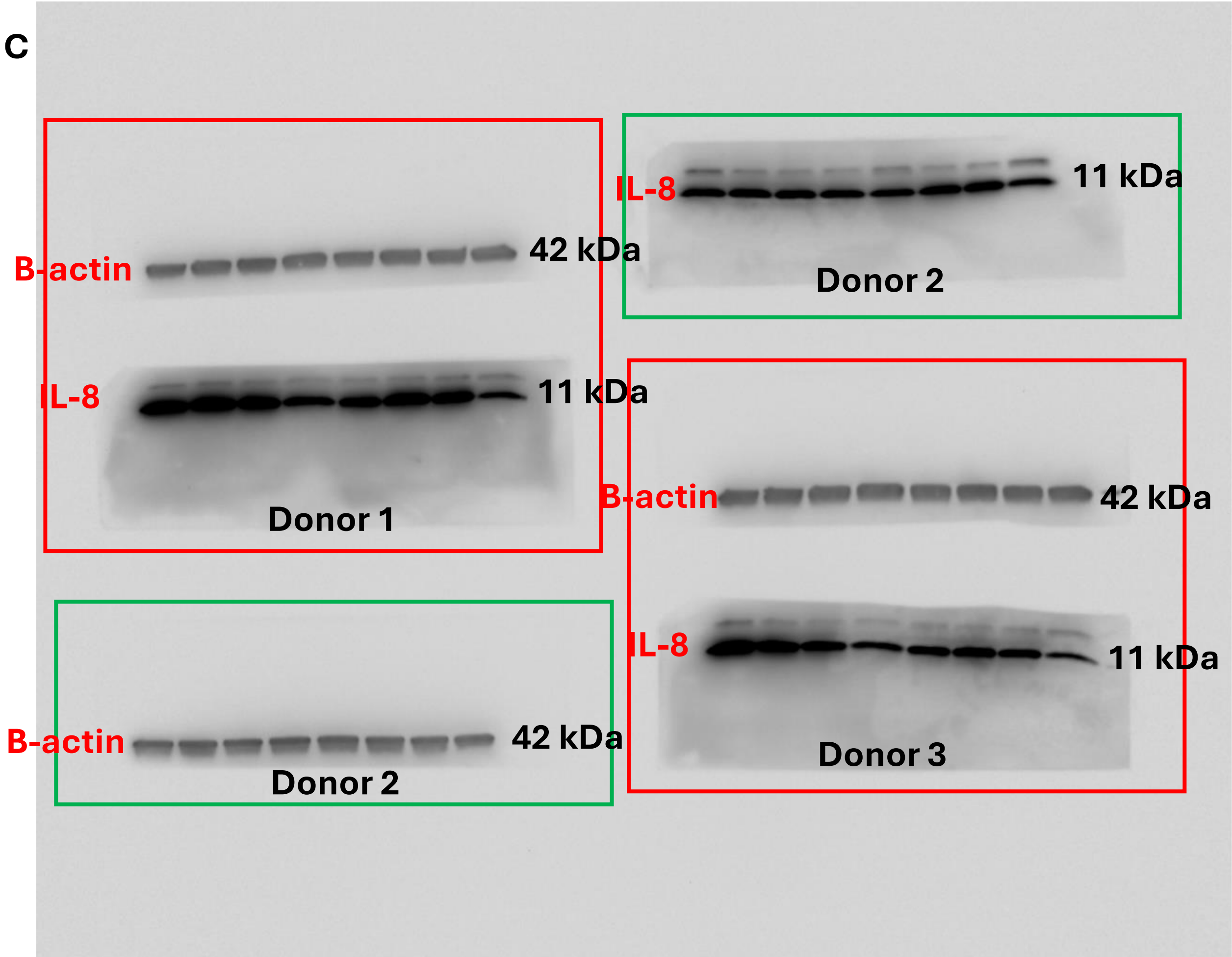

D

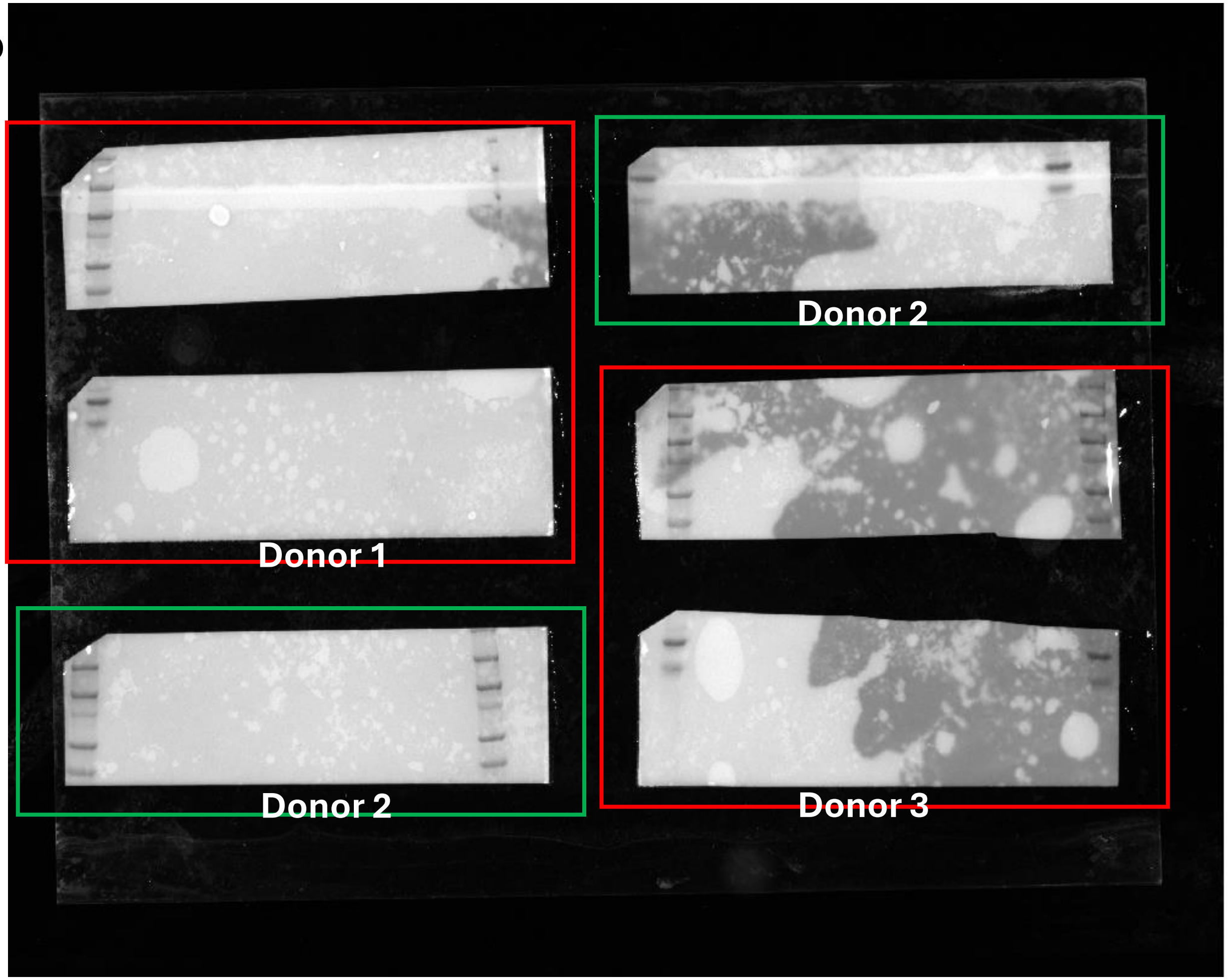

Supplement: Unedited blot and gel images [file jciinsight-10-191274-s235.pdf]
